# SCF Ubiquitin Ligase F-box Protein Fbx15 Controls Nuclear Co-repressor Localization, Stress Response and Virulence of the Human Pathogen *Aspergillus fumigatus*

**DOI:** 10.1371/journal.ppat.1005899

**Published:** 2016-09-20

**Authors:** Bastian Jöhnk, Özgür Bayram, Anja Abelmann, Thorsten Heinekamp, Derek J. Mattern, Axel A. Brakhage, Ilse D. Jacobsen, Oliver Valerius, Gerhard H. Braus

**Affiliations:** 1 Department of Molecular Microbiology and Genetics and Göttingen Center for Molecular Biosciences (GZMB), Georg-August-University, Göttingen, Germany; 2 Department of Biology, Maynooth University, National University of Ireland, Maynooth, County Kildare, Ireland; 3 Department of Molecular and Applied Microbiology, Leibniz Institute for Natural Product Research and Infection Biology (HKI), Friedrich Schiller University, Jena, Germany; 4 Research Group Microbial Immunology, Leibniz Institute for Natural Product Research and Infection Biology (HKI), Friedrich Schiller University, Jena, Germany; Texas A&M University, UNITED STATES

## Abstract

F-box proteins share the F-box domain to connect substrates of E3 SCF ubiquitin RING ligases through the adaptor Skp1/A to Cul1/A scaffolds. F-box protein Fbx15 is part of the general stress response of the human pathogenic mold *Aspergillus fumigatus*. Oxidative stress induces a transient peak of *fbx15* expression, resulting in 3x elevated Fbx15 protein levels. During non-stress conditions Fbx15 is phosphorylated and F-box mediated interaction with SkpA preferentially happens in smaller subpopulations in the cytoplasm. The F-box of Fbx15 is required for an appropriate oxidative stress response, which results in rapid dephosphorylation of Fbx15 and a shift of the cellular interaction with SkpA to the nucleus. Fbx15 binds SsnF/Ssn6 as part of the RcoA/Tup1-SsnF/Ssn6 co-repressor and is required for its correct nuclear localization. Dephosphorylated Fbx15 prevents SsnF/Ssn6 nuclear localization and results in the derepression of gliotoxin gene expression. *fbx15* deletion mutants are unable to infect immunocompromised mice in a model for invasive aspergillosis. Fbx15 has a novel dual molecular function by controlling transcriptional repression and being part of SCF E3 ubiquitin ligases, which is essential for stress response, gliotoxin production and virulence in the opportunistic human pathogen *A*. *fumigatus*.

## Author Summary

The opportunistic human fungal pathogen *Aspergillus fumigatus* is the most prevalent cause for severe fungal infections in immunocompromised hosts. A major virulence factor of *A*. *fumigatus* is its ability to rapidly adapt to host conditions during infection. The rapid response to environmental changes underlies a well-balanced system of production and degradation of proteins. The degradation of specific target proteins is mediated by ubiquitin-protein ligases (E3), which mark their target proteins with ubiquitin for proteasomal degradation. Multisubunit SCF Cullin1 Ring ligases (CRL) are E3 ligases where the F-box subunit functions as a substrate-specificity determining adaptor. A comprehensive control of protein production includes global co-repressors as the conserved Ssn6(SsnF)-Tup1(RcoA) complex, which reduces transcription on multiple levels. We have identified a novel connection between protein degradation and synthesis through an F-box protein. Fbx15 can be incorporated into SCF E3 ubiquitin ligases and controls upon stress the nuclear localization of the SsnF. Fbx15 plays a critical role for *A*. *fumigatus* adaptation and is essential for virulence in a murine infection model. Fbx15 is a fungal-specific protein and therefore a potential target for future drug development.

## Introduction

The ubiquitin 26S proteasome system (UPS) controls the life span of specific regulatory proteins, which are required for coordinated development, signal transduction and DNA maintenance. Target proteins are linked to ubiquitin by the sequential action of E1, E2 and E3 enzymes. A crucial step during this enzymatic cascade is carried out by E3 ubiquitin ligases, which recognize their specific substrate and catalyze the transfer of ubiquitin. SCF-complexes are multi-subunit E3 enzymes consisting of three major subunits (Cul1, Skp1 and Rbx1), which form the core enzyme and an exchangeable set of substrate-specific adaptors called F-box proteins [1,2]. The F-box domain of these adaptors is an N-terminal binding site of approximately 45 amino acids. It binds to the Skp1 linker to connect to Cul1. The human genome encodes 69 F-box proteins and defects in F-box mediated ubiquitination are associated with various diseases like diabetes, Parkinson or cancer [3–5]. Only little is known about the role of F-box proteins in virulence of fungal pathogens, though fungal F-box proteins play important roles for cellular development, transcription, signal transduction and nutrient sensing [6–8].

*Aspergillus fumigatus* is a soil borne, ubiquitously distributed filamentous fungus, growing on organic matter [3,9]. Besides its saprophytic lifestyle *A*. *fumigatus* also acts as an opportunistic human pathogen and causes life-threatening invasive pulmonary aspergillosis (IPA) in immunocompromised hosts. High mortality rates of up to 90% among infected patients are linked to azole resistance, the lack of new antifungals and increasing numbers of immunosuppressive therapies [9–12]. Great efforts have been conducted to identify virulence factors, which discriminate *A*. *fumigatus* from its closely related but significantly less pathogenic relative *Aspergillus nidulans* [13–19]. Virulence of *A*. *fumigatus* is presumably the result of a complex multifactorial network, rather than unique and sophisticated virulence factors. *A*. *fumigatus* pathogenicity is based on small infectious conidia and its ability to rapidly adapt to constantly changing conditions including high temperature, nutritional changes, hypoxia or high pH [4,20]. This is further supported by the production of secondary metabolites (SM) such as melanins, which protect from UV radiation or the immunosuppressive mycotoxin gliotoxin [21–23].

The rapid responses of *A*. *fumigatus* to environmental stressors are linked to distinct evolutionary conserved molecular mechanisms, which are often part of development regulating processes [1,2]. A recently identified important developmental regulator in *A*. *nidulans* is Fbx15, which is required for sexual and asexual development. Furthermore, Fbx15 accumulates in SCF^Fbx15^ complexes in *csn*-deficient mutants of *A*. *nidulans* [3–5]. The COP9 signalosome (CSN) multi-subunit complex plays a crucial role in fruiting body formation, oxidative stress tolerance and SM production in *A*. *nidulans* [6,7,24,25]. CSN acts as deneddylase by removing the isopeptide bond of the ubiquitin-like protein Nedd8 from a lysine residue of cullin scaffolds of those E3 ligases, which are not interacting with substrate molecules for ubqituitination [3,9]. Repetitive cycles of cullin neddylation/deneddylation are especially important for development because they promote the exchange of F-box adaptors from SCF E3 ligases [9–12]. The genomes of *A*. *nidulans* or its pathogenic counterpart *A*. *fumigatus* comprise approximately 70 F-box protein encoding genes, of which three (*fbx15*, *fbx23*, *grrA*) have been reported to influence developmental steps in *A*. *nidulans* [4,13–18,26].

In this study we characterized the molecular function of the Fbx15 counterpart of the pathogen *A*. *fumigatus*. We were originally interested whether F-box proteins, which play a crucial role for fungal development in *A*. *nidulans* are connected to *A*. *fumigatus* pathogenicity. We found a novel dual function for F-box proteins because Fbx15 is not only part of nuclear SCF complexes but also controls the nuclear localization of the SsnF/Ssn6 component of the highly conserved eukaryotic transcriptional co-repressor complex Ssn6/Tup1. Cellular Fbx15 function is controlled by posttranslational phosphorylation and dephosphorylation during stress. Fbx15 including the F-box domain is required for cellular stress responses, the control of gliotoxin production and for virulence in a mouse model. Fbx15 is fungal-specific and might therefore be an interesting novel target for new drugs to treat invasive aspergillosis.

## Results

### Fbx15 is essential for a general stress response in *Aspergillus fumigatus*

**Fig 1:**
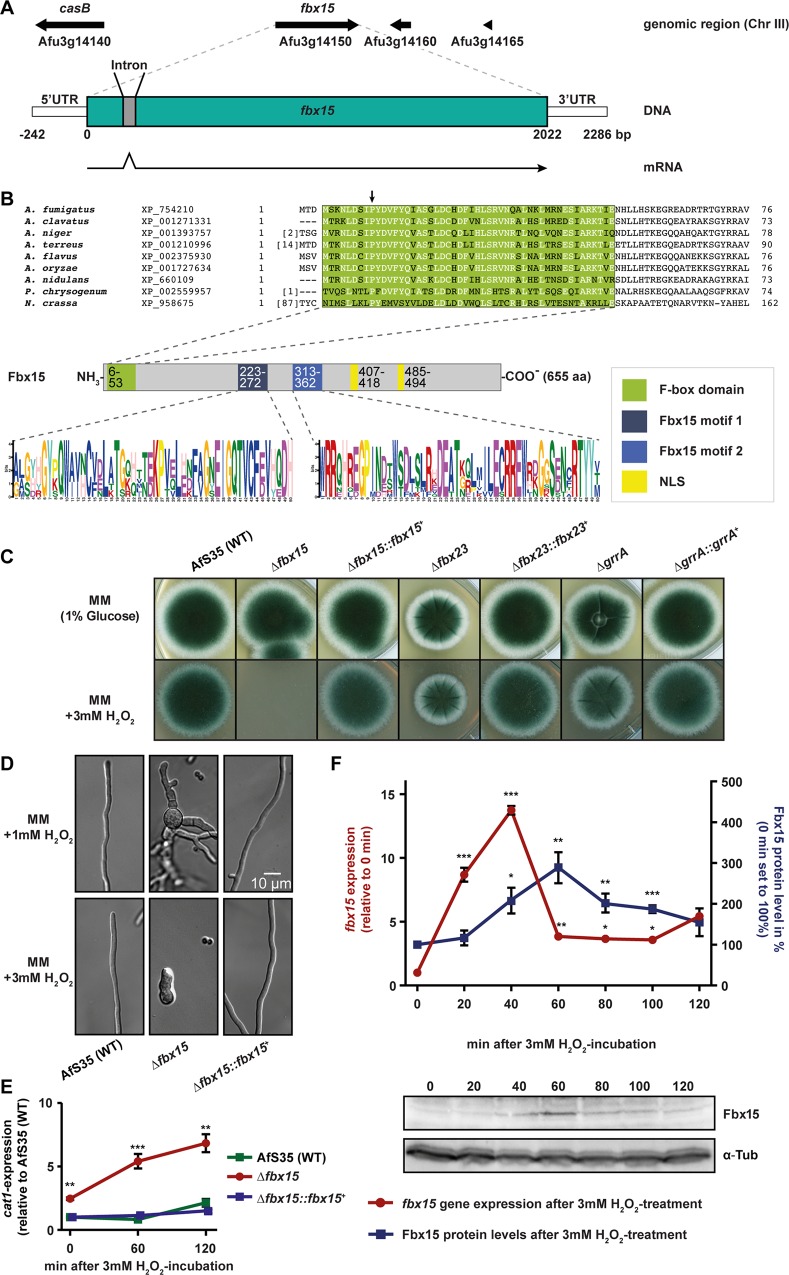
*Aspergillus fumigatus* F-box domain protein Fbx15 is indispensible for oxidative stress response. (A) *fbx15* genomic locus, structure and transcripts. (B) The primary sequence of *A*. *fumigatus* Fbx15 F-box domain was compared with fungal homologues by ClustalW alignment. Highly conserved amino acids are marked in white; an arrow indicates the characteristic proline residue at position seven of the F-box domain. (C) *fbx* deletion mutants Δ*fbx15* and to less extent Δ*fbx23* and Δ*grrA* showed increased oxidative stress sensitivity, which could be complemented by reintroduction of the wild type *fbx*-genes. (D) Δ*fbx15* mutant showed hyphal defects at low H_2_O_2_ concentrations. (E) Expression levels of *cat1* encoding a mycelial catalase were increased in the Δ*fbx15* mutant, whereas wild type and complemented strain showed only slightly elevated *cat1* mRNA levels after H_2_O_2_ exposure. Mean values ± s.d. of *N* = 3 independent experiments are shown. *P*-values were calculated using two-sample *t*-test (**P<0.01; ***P<0.001). (F) *fbx15* transcript expression in AfS35 (WT) showed a rapid increase in the first 20 min upon oxidative stress and with a short delay increased protein levels, which are detected by an Fbx15 specific antibody. Mean values ± s.d. of *N* = 3 independent experiments are shown. *P*-values were calculated using two-sample *t*-test (*P<0.05; **P<0.01; ***P<0.001).

*A*. *fumigatus fbx15* (Afu3g14150) corresponds to the gene of *A*. *nidulans* encoding F-box protein 15, which is required for development [4,20]. Several cDNAs of this gene locus were sequenced and revealed that the *A*. *fumigatus fbx15* gene structure consists of two exons and one intron resulting in a deduced open reading frame of 655 codons for a protein with a predicted molecular mass of 75 kDa (Fig 1A). Alignments of the *A*. *fumigatus* Fbx15 primary sequence revealed that high similarities are restricted to the Aspergilli counterparts with similarities between 72.8% and 59.8%, whereas Fbx15-like proteins of other filamentous fungi like *Penicillium chrysogenum* or *Neurospora crassa* share significant lower similarities of 43.6% and 24.7% respectively (Fig 1B, S1 Table). Two signature motifs are located almost in the middle of Fbx15 and include motif 1, which is specific for the genus Aspergillus and motif 2 that is also present in other fungi as *P*. *chrysogenum* or *N*. *crassa* (Fig 1B, S2 Table). Bioinformatics analysis predicts two nuclear localization signals (NLS), which suggest that Fbx15 might exhibit its function in the nucleus (Fig 1B).

The function of *fbx15* in *A*. *fumigatus* was genetically addressed by creating an *fbx15* deletion. The mutant strain formed normal colonies on minimal medium (MM), but oxidative stress caused by 3 mM H_2_O_2_ abolished growth, whereas the wild type could still grow (Fig 1C). 1 mM H_2_O_2_, which had no impact on wild type, resulted in hyperbranched, swollen hyphae in the Δ*fbx15* strain (Fig 1D). On media containing superoxides-producing menadione or thiol-oxidizing diamide the Δ*fbx15* mutant displayed defects in growth and sporulation, which implies a general Fbx15 mediated resistance mechanism against oxidative stress, upstream of distinct stress response pathways (S1B Fig). Severe growth and sporulation defects were also observed when the Δ*fbx15* mutant was exposed to other stress conditions including elevated temperature, amino acid starvation, microtubule stress, osmotic stress or mutagenic stresses (S1C Fig).

The importance of Fbx15 during stress was compared to other F-box proteins, which have been previously shown to be involved in fungal development. Fbx23 is important to initiate *A*. *nidulans* asexual development [4,21–23], whereas GrrA is an F-box protein that is required for the maturation of sexual spores [26–29]. These two F-box proteins are conserved from fungi to higher eukaryotes (S1 Table). Characterization of both deletion mutants in *A*. *fumigatus* showed more distinct and less prominent phenotypes compared to the Δ*fbx15* mutant strain. In contrast to the wild type, deletion of *fbx23* led to a decreased colony size, whereas the colony size of the Δ*grrA* mutant was not affected under normal growth conditions (Fig 1C).

Growth defects of Δ*fbx23* and Δ*grrA* in comparison to wild type were observed under amino acid starvation or microtubule stress. Both deletion strains were sensitive to topoisomerase I inhibitor CPT, whereas DNA-methylating MMS had a similar impact on wild type and mutant growth (S1C Fig). These results indicated that Fbx23 as well as GrrA are involved in the DNA replication process, whereas Fbx15 is additionally required for the repair of DNA-damage caused by methylation. Growth under increased temperature or oxidative stress conditions are important virulence factors of *A*. *fumigatus* [30]. The deletion of *fbx23* led to a reduced colony size under high temperature (42°C) or oxidative stress, when compared to the wild type, whereas the Δ*grrA* strain was only slightly affected by H_2_O_2_ (Fig 1C, S1C Fig).

These data suggest that the three F-box proteins Fbx23, GrrA and Fbx15 are part of a general stress response in *A*. *fumigatus* caused by multiple environmental stressors. The impact of the strain lacking Fbx15 suggests that this protein plays a key role in the fungal stress response.

### Oxidative stress transiently induces *fbx15* expression

Oxidative stress caused drastic effects on the Δ*fbx15* mutant, whereas *fbx15* overexpression resulted in a wild type like phenotype (S1A Fig). Oxidative stress, in particular by peroxides like H_2_O_2_, leads to an elevated expression of catalases, which function as potent ROS scavenger [31,32]. *A*. *fumigatus* produces one conidial and two mycelial catalases. The expression of *cat1*, which encodes one of the mycelial catalases, was regulated in an Fbx15 dependent manner. The lack of *fbx15* led to an approximately 2.5x derepressed basal *cat1* expression, compared to wild type or a complemented strain during non-stress conditions (Fig 1E). After exposure to H_2_O_2_, the expression of *cat1* in the Δ*fbx15* mutant was further increased, whereas wild type or complemented strain displayed only slight upregulation. Increased *cat1* levels were not sufficient to protect the Δ*fbx15* mutant from oxidative stress. Fbx15 presumably is involved in additional molecular mechanisms, which protect against oxidative stress. The exposure to oxidative stress was analyzed as a possible external signal, which triggers changes in *fbx15* expression in *A*. *fumigatus*. Fungal cultures were exposed to H_2_O_2_ and harvested at different time points within a 120 min period. *fbx15* transcript levels were determined with real-time PCR (RT-PCR). A rapid increase of *fbx15* expression was observed in the first 20 min reaching its maximum peak at 40 min with a 14-fold increased gene expression. Afterwards the expression decreased to a basal level, which is approximately 4-fold increased compared to the non-induced expression. Proteins from the same samples were extracted to test whether the changes in *fbx15* transcript levels are also reflected on protein level. Fbx15 was visualized after immunoblotting by incubation with a polyclonal Fbx15 specific antibody. The general abundance of Fbx15 was very low. A three-fold increase of Fbx15 protein amounts was measured, starting after 40 min of H_2_O_2_ exposure, which was similar in the complemented strain and absent in the Δ*fbx15* mutant, mirroring the increased gene expression on the protein level with a delay of 20 min (Fig 1F, S1D Fig). These data suggest that the Fbx15 protein levels are increased as part of a stress adaption response towards H_2_O_2_ mediated ROS.

### Fbx15 is predominantly localized in the nucleus

Two putative nuclear localization sites of Fbx15 suggest a nuclear function. A similar situation is found for the conserved *A*. *fumigatus* F-box protein SconB, which also possesses two nuclear localization signals, and was used as control (S1 Table). Similar to *sconB* of *A*. *nidulans*, *sconB* is essential for *A*. *fumigatus* as we could show with heterokaryon rescue assay (S2A and S2B Fig). Constitutively expressed GFP fusions of Fbx15 and SconB were compared for their subcellular localizations. Both F-box GFP fusions were functional during normal and oxidative stress growth conditions (S2C Fig). Fbx15 and SconB are primarily co-localized in fluorescence microscopy with DAPI stained nuclei, though small subpopulations of both proteins remain in the cytoplasm (S2D Fig). These data suggest a predominant nuclear function for both *A*. *fumigatus* F-box proteins.

### Fbx15 is phosphorylated during vegetative growth under non-stress conditions

**Fig 2:**
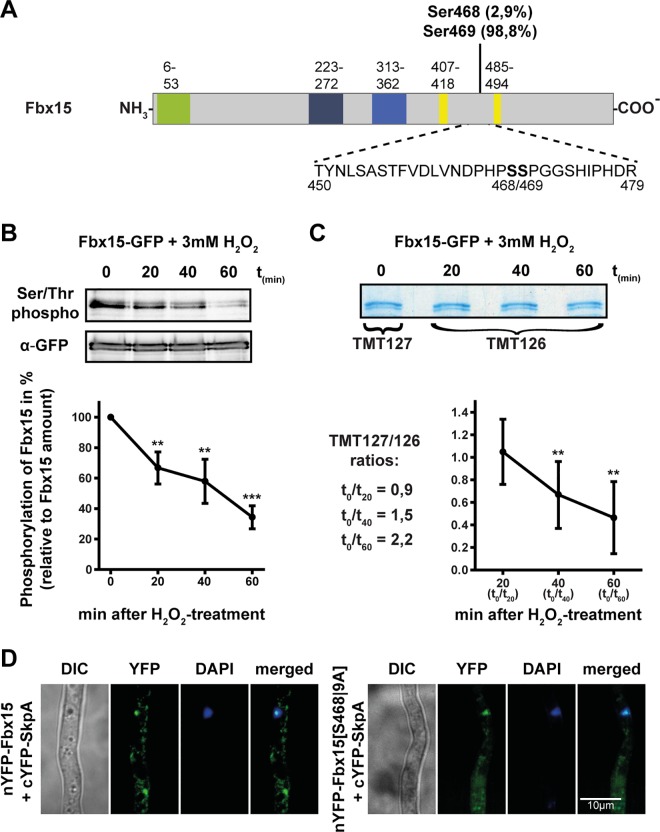
Fbx15 phosphorylation decreases during oxidative stress treatment and shifts the cellular interaction site with SkpA to the nucleus. (A) Phosphorylated peptide of Fbx15 identified by LC-MS/MS after TAP-purification. Phosphorylation probabilities are based on NetPhos 2.0. (B) Immunoblotting of purified Fbx15-GFP before and after 3 mM H_2_O_2_ treatment with an S/T-phospho-specific antibody revealed rapid dephosphorylation of Fbx15. The graph represents mean values ± s.d. of *N* = 3 independent experiments, with S/T-phospho-specific signal intensities normalized to total Fbx15 amount detected by GFP-specific antibody. Normalized Signal intensities were analyzed using two-sample *t*-test (**P<0.01; ***P<0.001). (C) Peptides of purified Fbx15-GFP before and after incubation with 3 mM H_2_O_2_ were differentially labeled with TMT isobaric mass labeling reagent. LC-MS/MS determined ratios of the TMT reporter-ion intensities for the MS2-spectra of the phosphopeptide are given. The graph on the right represents the reciprocal values, which confirmed specific dephosphorylation of S468/469 upon oxidative stress. Ratios of the TMT reporter-ion intensities from normalized phosphopeptides of *N* = 2 independent experiments were analyzed by one-way ANOVA followed by Tukey’s HSD test (**P<0.01). (D) BiFC signals of Fbx15 and SkpA occurred mostly in the cytoplasm, whereas unphosphorylated Fbx15[S468|9A] interacted with SkpA primarily in the nucleus.

Fbx15 is primarily nuclear but has a significant cytoplasmic subpopulation. Different localizations of Fbx15 could be a result of controlled posttranslational phosphorylation. A bioinformatic analysis of the deduced amino acid sequence of Fbx15 with NetPhos 2.0 (http://www.cbs.dtu.dk/services/NetPhos) [33–35] predicts in total 15 serine, 11 threonine and 4 tyrosine residues as putative phosphorylation sites (score value between 0 and 1; cutoff value >0.5: S3A Fig). An Fbx15 phosphopeptide with a single phosphorylation was identified with mass-spectrometry of purified functional Fbx15-TAP fusions from non-stressed cultures. Analysis of the MS2-spectra of this phosphopeptide with the phosphoRS software [36] revealed serine residues 468 and 469 as potential phosphorylation sites with probabilities of 45.5% each, whereas Ser473 only showed a low probability of 8.9% (Fig 2A). Theoretical analysis for Fbx15 phosphosites showed a high score value of 0.988 for Ser469, whereas Ser468 had a low score value of 0.029 and therefore is unlikely to be phosphorylated (S3A Fig). In contrast no phosphopeptides were identified for purified Fbx15-GFP fusions when the cells were grown under oxidative stress. Fbx15 is presumably phosphorylated during vegetative growth under non-stress conditions at Ser468 or Ser469 (Ser468/469), whereas it is unphosphorylated when H_2_O_2_-mediated oxidative stress is applied.

### Phosphorylated Fbx15 interacts with the GlcA/BimG phosphatase and is dephosphorylated during oxidative stress

Phosphorylation of Fbx15 under normal growth conditions and dephosphorylation during H_2_O_2_-mediated oxidative stress suggests the presence of a phosphatase that might be specifically activated. GFP-traps with Fbx15-GFP recruited GlcA as the only phosphatase that interacts in cultures with or without stress (S3B Fig). A direct interaction of Fbx15 with GlcA could be observed using bimolecular fluorescence complementation (BiFC), which was primarily visible after induction with H_2_O_2_ (S3C Fig). The *A*. *nidulans* GlcA homolog BimG has been characterized as essential major protein phosphatase 1, which is associated with thermo tolerance and hyphal morphology, features that were impaired in the Δ*fbx15* mutant [37,38]. Heterokaryon rescue experiments with a *glcA* deletion cassette and accompanying Southern-hybridizations verified that the situation is similar and *glcA* is essential for *A*. *fumigatus* as well (S3C and S3D Fig).

The dephosphorylation rate of Fbx15 in response to H_2_O_2_ was quantified. Fbx15-GFP expressing strains were grown in liquid cultures and subjected to oxidative stress by adding 3 mM H_2_O_2_. Fbx15-GFP from these cultures was purified and treated with an antibody against phosphorylated Ser/Thr residues. The rate of dephosphorylation was quantified against the overall amount of Fbx15-GFP, determined by an anti-GFP antibody (Fig 2B). 40% dephosphorylation upon H_2_O_2_-treatment indicated that Fbx15 becomes dephosphorylated in an oxidative stress-dependent manner. The Fbx15 dephosphorylation sites were localized by TMT isobaric mass tag labeling and LC-MS/MS [39]. Fbx15-GFP was enrichment from cultures before and after H_2_O_2_ treatment and separated on a coomassie-stained SDS-PAGE. After tryptic digestion, peptides from the untreated culture were labeled with TMT127 (heavy) whereas TMT126 (light) was used for all time points of the H_2_O_2_ treated cultures. The sample of time point zero was individually mixed with all other time points and analyzed by LC-MS/MS. The parameters for fragmentation during mass spectrometry were set to identify only the phosphorylated peptide of Fbx15. Specific ratios of the heavy labeled phosphopeptide were obtained from time point zero against the light labeled phosphopeptides from the other conditions. These values were quantified against the ratios of two unmodified reference peptides of Fbx15, which represented the overall amount of purified Fbx15. The reciprocal values of these ratios are decreasing, thus reflecting a specific dephosphorylation on Ser468/469 (Fig 2C). Fbx15, which becomes phosphorylated at Ser468/469 under non-stress conditions, is presumably dephosphorylated when cells encounter H_2_O_2_-mediated oxidative stress by the Fbx15 interacting phosphatase GlcA/BimG.

### Fbx15 dephosphorylation shifts the interaction with SkpA from the cytoplasm into the nucleus

The canonical function of F-box proteins is their ability to form ubiquitinating SCF ligase complexes by binding to the SkpA adaptor. *A*. *fumigatus* Fbx15 and SconB interactions to the SkpA SCF adaptor were compared by using BiFC. Both Fbx15 and SconB protein fusions produced an YFP-signal, indicating F-box protein-SkpA interactions. SconB interacted with SkpA almost exclusively in the nucleus (>90%), whereas 74% of the Fbx15-SkpA interaction was cytoplasmic (Fig 2D and S4A Fig). The Fbx15 serine codons of wild type positions S468 and S469 were replaced to alanine residues to mimic a constantly dephosphorylated Fbx15[S468|9A] to analyze whether Fbx15 phosphorylation is relevant for the location of the Fbx15-SkpA interaction. Interaction of Fbx15[S468|9A] variant with SkpA resulted in a nuclear signal (Fig 2D). Dephosphorylated Fbx15 primarily interacts with SkpA in the nucleus to form SCF-complexes. Growth without stress rather results in phosphorylated Fbx15 with presumably only limited amounts of dephosphorylated Fbx15 in the nucleus.

### Phosphorylated and unphosphorylated Fbx15-SkpA heterodimers can interact with CulA

**Fig 3:**
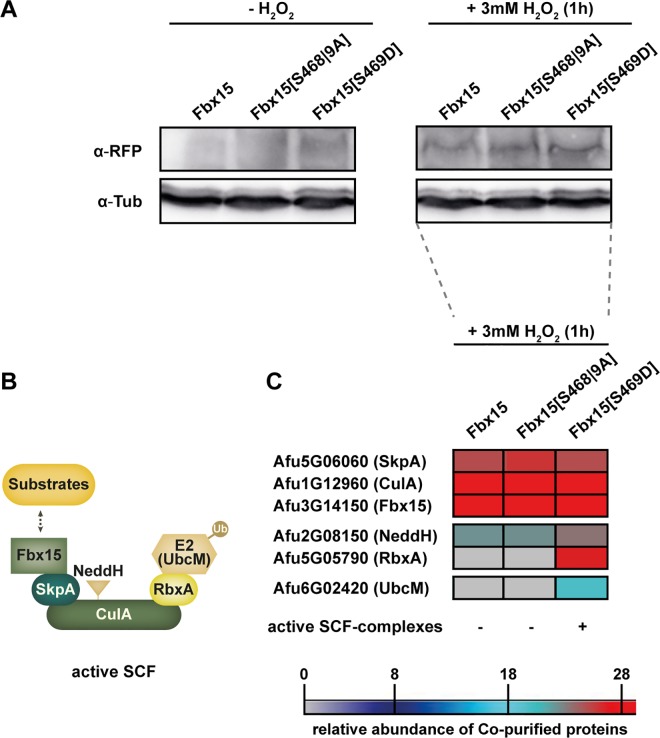
Phosphorylated or unphosphorylated Fbx15 can interact with SkpA and CulA. (A) Immunohybridization of Fbx15-RFP and phosphomutant versions of Fbx15, which mimic either an unphosphorylated Fbx15[S468|9A]-RFP or a constant phosphorylation Fbx15[S469D]-RFP before and after H_2_O_2_-mediated oxidative stress. (B) Scheme of an active SCF-complex. (C) Heatmap of co-purified SCF subunits for RFP-tagged Fbx15 and phosphomutant versions after oxidative stress.

Dephosphorylation of Fbx15 is triggered by H_2_O_2_-mediated ROS. The impact of the phosphorylation state of Fbx15 on its ability to interact with SCF-complexes was analyzed by replacing the serine residue S469 in Fbx15 with aspartate to mimic a constant phosphorylation Fbx15[S469D] or S468 and S469 with alanine to mimic unphosphorylated Fbx15[S468|9A]. Both constructs and the wild type gene were expressed under the native promoter as Fbx15-RFP fusions. Immunoblotting confirmed that all Fbx15 versions were more abundant after H_2_O_2_ exposure (Fig 3A).

RFP-trap co-purifications followed by LC-MS/MS with wild type Fbx15 or phosphomutant variants after oxidative stress induction with H_2_O_2_ were performed to identify which subunits of the SCF-ligase machinery interact with Fbx15. Further analysis included MaxQuant quantitative proteomic software in conjunction with Perseus software for statistical analysis, with a focus on the subunits of the SCF-ligase machinery. SCFs are activated by the RING protein RbxA-mediated interaction with E2 ubiquitin-conjugating enzyme and covalent cullin modification by ubiquitin like NeddH (Fig 3B). SCF core components SkpA or CulA interacted with Fbx15, independently of the phosphorylation-state. Native Fbx15, which is presumably dephosphorylated at S468/S469 after oxidative stress or unphosphorylated Fbx15[S468|9A] could co-purify NeddH, but not RbxA or an E2 enzyme. In contrast the Fbx15, mimicking a constant phosphorylation, co-purified all subunits for an active SCF complex. NeddH was more abundant in co-purifications of the constantly phosphorylated Fbx15[S469D] than of unphosphorylated versions of Fbx15. RbxA and the E2 enzyme UbcM were only purified with negatively charged Fbx15[S469D], indicating an improved assembly of functional SCF-ligase complexes when Fbx15 is phosphorylated at Ser469 (Fig 3C). Active SCF^Fbx15^ complexes contain therefore more likely phosphorylated than unphosphorylated Fbx15.

Since Fbx15 interacted within SCF complexes the cellular ubiquitination pattern of the Δ*fbx15* mutant, wild type and the *fbx15* overexpression strain were compared before and after induction with H_2_O_2_. Neither the ubiquitination-pattern nor the general protein composition of the Δ*fbx15*-strain was significantly altered in comparison to the wild type or the *fbx15* overexpression strain, suggesting that the ubiquitination targets of SCF^Fbx15^ complexes are limited (S4B and S4C Fig).

### Fbx15 recruits 38 putative interacting proteins during TAP-enrichment

Additional potential interacting proteins for Fbx15 were identified by tandem-affinity-purification (TAP) and compared to co-purifications of SconB-TAP fusions. Variants of *fbx15* and *sconB* were created where the conserved proline of the F-box domain was exchanged to a serine. This should weaken the F-box-SkpA binding and enable recruitment of SCF-independent interaction partners. The exchanged proline in SconB led to significantly increased protein stability, whereas stability of Fbx15 was not altered (S5 Fig). This reflects a possible autocatalytic mechanism for SCF^SconB^, where SconB is ubiquitinated within its own SCF ligase and eventually degraded [40,41], whereas Fbx15 stability seems to be independent of SCF^Fbx15^. SconB-TAP recruited less proteins (22) than Fbx15 (38). SconB interactors include transcriptional activator MetR as known SCF^SconB^ target [1] and 11 proteins that were identified for both F-box proteins including SCF subunits CulA and SkpA. Only Fbx15 was able to co-purify three subunits of the CSN deneddylase, which acts on neddylated cullin complexes, which do not interact with substrates [3]. This might reflect a highly dynamic assembly/disassembly of SCF^Fbx15^ complexes (S3 Table).

**Fig 4:**
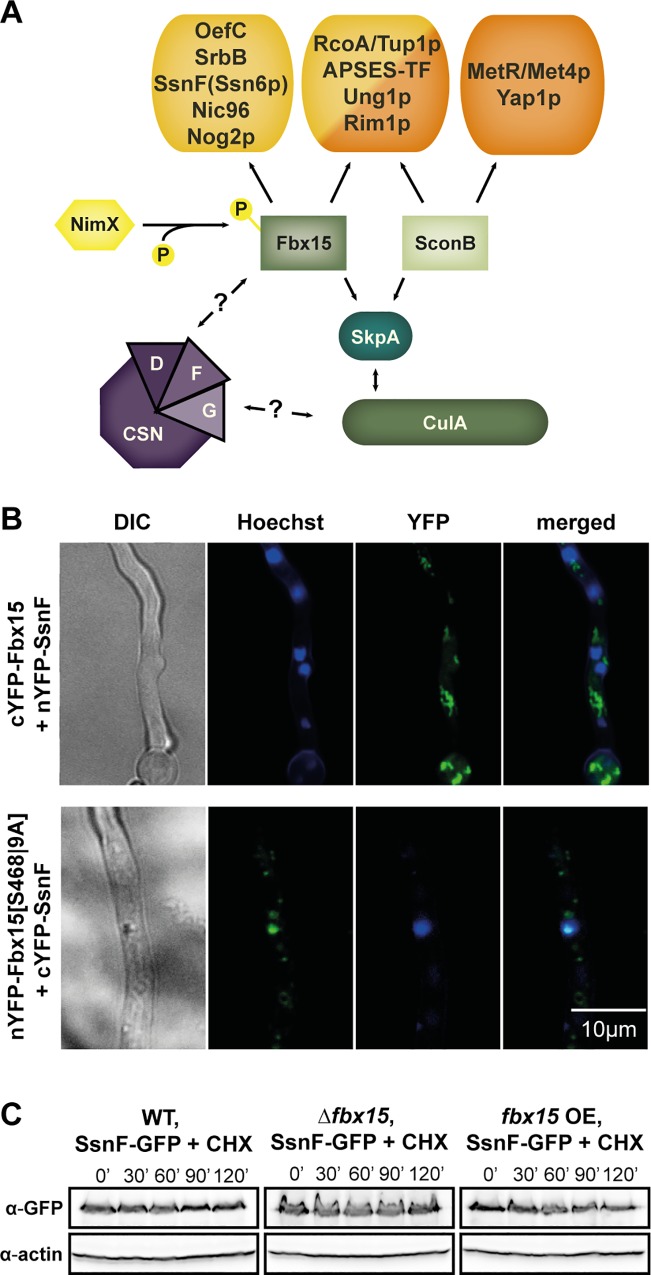
Fbx15 interacts with the transcriptional repressor subunit SsnF without affecting its stability. (A) Scheme of a subset of interacting proteins of Fbx15 and SconB based on LC-MS/MS identifications. Shown are presumably nuclear proteins, which are either exclusively found for each F-box protein or which were found for both. Further Fbx15-TAP co-purified proteins included three CSN subunits and the cyclin-dependent kinase NimX. (B) BiFC of Fbx15 and SsnF showed a predominantly cytoplasmic YFP-signal, whereas unphosphorylated Fbx15[S468|9A] interacted with SsnF primarily in the nucleus. (C) Protein stability assays of SsnF-GFP in wild type, Δ*fbx15* or *fbx15* overexpression backgrounds with cultures exposed to cycloheximide (CHX) showed no Fbx15-dependent SsnF stability changes.

The predominant nuclear localization of Fbx15 and SconB is consistent with nuclear interaction partners, which were identified during TAP co-purifications. These included two transcriptional regulators (RcoA/Tup1 and a putative APSES transcription factor), a DNA repair enzyme (AFUA_2G06140) and a single-stranded DNA binding protein (AFUA_5G07890/Rim1p) (Fig 4A). The fact that Fbx15 and SconB recruited these proteins might reflect a tight stability control by more than one F-box protein. In addition Fbx15 recruited specifically three transcriptional regulators (OefC, SrbB and SsnF/Ssn6), a nuclear GTPase (AFUA_4G8930/Nog2p) and the nuclear pore protein Nic96. (Fig 4A). A potential candidate, responsible for Fbx15 phosphorylation is the interacting cyclin-dependent serine/threonine kinase NimX/Cdc28p, which is required for cell cycle control and conidiophore morphology in *A*. *nidulans* [6]. A direct interaction of Fbx15 and NimX could be verified by BiFC assay, where the interaction signal from the reconstituted YFP was observed predominantly in the cytoplasm (S6A Fig). However, the NimX homolog in *A*. *fumigatus* is presumably encoded by an essential gene as we could show by heterokaryon assay and Southern hybridization (S6B and S6C Fig).

Fbx15 and SconB interaction partners include possible targets for SCF mediated ubiquitination. Three Fbx15 interacting CSN subunits suggest a more dynamic assembly/disassembly of SCF^Fbx15^ in comparison to SCF^SconB^. An interesting finding is that both F-box proteins co-purified RcoA/Tup1 as part of the conserved transcriptional co-repressor complex RcoA/Tup1-SsnF/Ssn6, but only Fbx15 recruited the SsnF/Ssn6 subunit of this complex.

### Fbx15 interaction with SsnF does not change the stability of the co-repressor subunit

The yeast Ssn6-Tup1 co-repressor complex affects the expression of 7% of all genes with emphasis on stress responses [9]. A homo-tetramer of RcoA/Tup1 repressor subunits is connected to one SsnF/Ssn6 adaptor protein, which binds to a DNA-binding protein, escorting the repressor complex to the target genes. The corresponding SsnF encoding gene of the model *A*. *nidulans* is essential for growth, whereas the yeast counterpart Ssn6 is dispensable [9,42]. With heterokaryon rescue and subsequent Southern analysis we could show that *ssnF* is not only essential for *A*. *nidulans* but also for *A*. *fumigatus* (S6D and S6E Fig). A BiFC signal verified the direct interaction of Fbx15 and SsnF in *A*. *fumigatus* under non-stress conditions in the cytoplasm, often located close to nuclei. Unphosphorylated Fbx15[S468|9A] which reflected H_2_O_2_-mediated oxidative stress conditions interacted with SsnF predominantly in the nucleus, similar to the Fbx15-SkpA interaction (Fig 4B). Fbx15 levels in different strain backgrounds did not influence SsnF stability in cycloheximide (CHX) protein-stability assays (Fig 4C). The amount of SsnF-GFP in wild type or *fbx15* deletion mutant did not change in the absence or presence of 3 mM H_2_O_2_ nor did SsnF exhibit an Fbx15 dependent ubiquitination pattern (S6F and S6G Fig). This suggests that SsnF is not a significant substrate of an active SCF^Fbx15^ E3 complex but acts as additional physical Fbx15 interaction partner.

### Fbx15 is required for correct nuclear localization of SsnF

**Fig 5:**
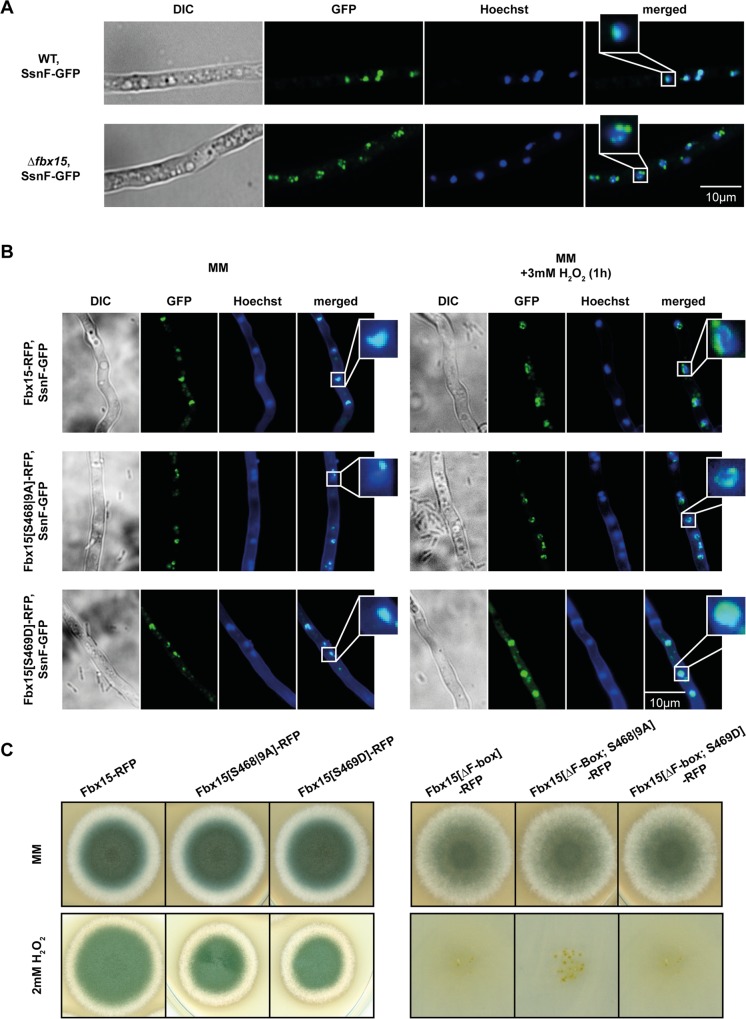
Fbx15 is required for nuclear localization of SsnF. (A) Fluorescence microscopy of SsnF-GFP in wild type or Δ*fbx15* background revealed that SsnF-GFP was localized to the periphery of the nuclei in Δ*fbx15*, whereas it was inside the nuclei in wild type. (B) SsnF-GFP localization in wild type Fbx15-RFP, unphosphorylated Fbx15[S468|9A]-RFP or phosphorylated Fbx15[S469D]-RFP in backgrounds before and after H_2_O_2_-treatment. Under normal growth conditions SsnF is nuclear in the presence of either the wild type or the phosphate mimicking Fbx15[S469D] variant, whereas SsnF-GFP accumulates at the nuclear envelope in presence of the non-phosphorylatable Fbx15[S468|9A]. Upon oxidative stress, the dephosphorylation of wild type Fbx15 leads to similar subpopulations of SsnF like the unphosphorylated Fbx15[S468|9A]. In phosphorylated Fbx15[S469D] SsnF enters the nucleus even after H_2_O_2_-treatment. (C) Growth tests of *A*. *fumigatus* strains expressing either wild type *fbx15*::*rfp* (AfGB98), *rfp* tagged phosphovariants of *fbx15* (AfGB101 and AfGB102) or corresponding *rfp*-tagged versions of *fbx15* that lack the F-box domain (AfGB125, AfGB126 and AfGB127). 5 x 10^3^ conidia were spotted on minimal medium (MM) plate, supplemented with 2 mM H_2_O_2_ for providing oxidative stress, and grown for four days at 37°C.

SsnF-GFP is localized in the nucleus in the presence of Fbx15, but accumulates at the nuclear envelope presumably enriched at nuclear pore complexes in the absence of Fbx15. This suggests that SsnF import is impaired without Fbx15 (Fig 5A). It was analyzed whether dephosphorylation of Fbx15 is involved in SsnF nuclear localization. In *fbx15* wild type as well as in the *fbx15* variant strain (Fbx15[S469D]), that mimics constant phosphorylation, SsnF-GFP was localized in the nucleus. In the *fbx15* codon replacement mutant expressing the Fbx15[S468|9A] variant, which cannot be phosphorylated, SsnF accumulated at the nuclear envelope similar to the Δ*fbx15* strain. Correct nuclear SsnF-GFP localization could be either abolished after Fbx15 wild type dephosphorylation due to H_2_O_2_ or in the presence of the Fbx15[S468|9A] variant, which cannot be phosphorylated. In contrast, oxidative stress did not interfere with SsnF-GFP nuclear localization, when only the negatively charged Fbx15[S469D] was present, which mimics constant phosphorylation (Fig 5B).

These data support that phosphorylation of Fbx15 under non-stress conditions favors nuclear localization of SsnF, whereas Fbx15 dephosphorylation during H_2_O_2_-mediated oxidative stress leads to an accumulation of SsnF at the nuclear envelope.

### The F-box of Fbx15 is essential and the phosphorylation status, which controls SsnF nuclear localization, contributes to the fungal stress response

The contribution of the Fbx15[S468|9A] variant, which cannot be phosphorylated and interferes with nuclear SsnF, to the fungal oxidative stress response was analyzed. Growth tests on different oxidative stress providing media showed that mutant strains expressing the non-phosphorylable Fbx15[S468|9A] as well as the phosphate mimicking Fbx15[S469D] showed mild colony growth reductions in comparison to the wild type version of Fbx15 (Fig 5C). Similar effects where observed on medium containing superoxide-producing menadione, where the phosphomutant strains showed a slight growth reduction compared to the RFP-tagged wild type Fbx15. Growth on thiol oxidizing media was not affected (S7 Fig). These data support that Fbx15 phosphorylation and dephosphorylation and the control of cellular SsnF localization contribute to an appropriate oxidative stress response.

The function for oxidative stress resistance of the F-box domain of Fbx15 as link to E3 ubiquitin ligases was examined. A gene for an RFP-tagged Fbx15 variant, that lacks the N-terminal F-box domain, and the two additional combinations with the alternative phosphovariants were constructed and the resistance against oxidative stress was tested. Loss of the F-box in all three strains completely abolished growth on media containing H_2_O_2_, similar to the Δ*fbx15* mutant (Fig 5C, S7 Fig).

These results indicate that the F-box, which is required to assemble Fbx15 into SCF ubiquitin ligases, is essential for the fungal oxidative stress response. The function of the oxidative stress controlled phosphorylation status of Fbx15, which channels SsnF nuclear localization, is presumably part of an additional fine-tuning of the appropriate cellular oxidative stress response.

### Fbx15 is required for the repression of gliotoxin biosynthesis

A group of genes, which are usually repressed during normal fungal growth, are secondary metabolite genes. Defects in the CSN-regulated ubiquitination machinery result in a drastic misregulation of secondary metabolite formation, such as mycotoxins [13]. A potent immunosuppressive mycotoxin in *A*. *fumigatus* is gliotoxin, which is considered as one of multiple virulence factors [20].

**Fig 6:**
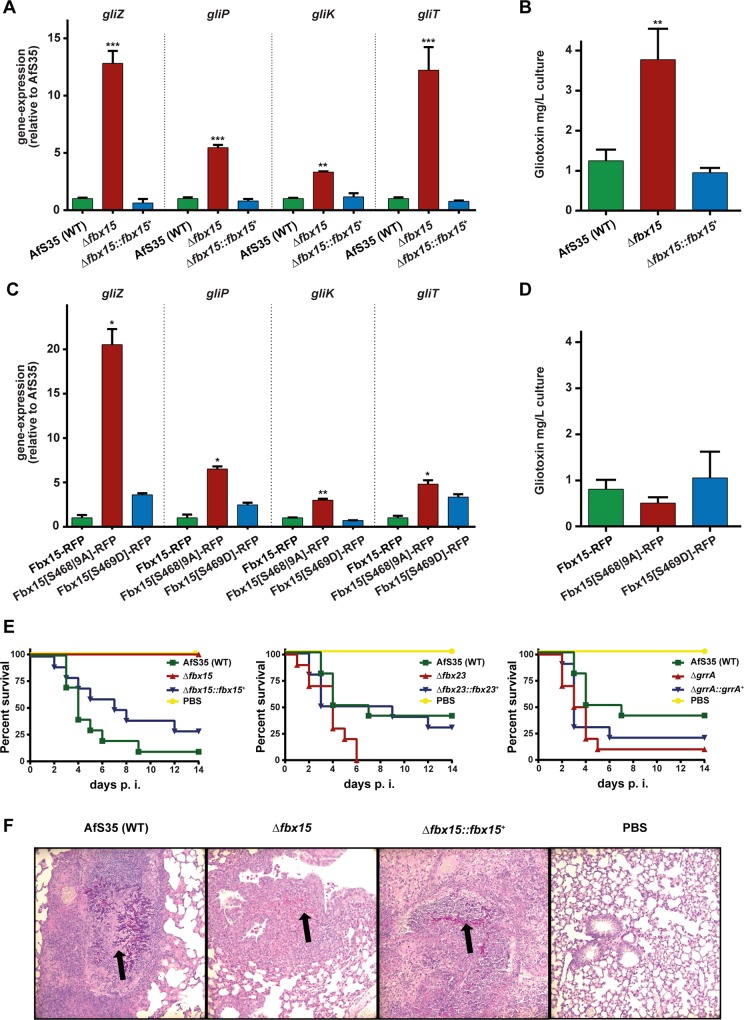
Deletion of *fbx15* results in elevated gliotoxin levels but a loss of virulence in a murine model of invasive pulmonary aspergillosis. (A) The Δ*fbx15* mutant showed an increased *gli* gene expression compared to wild type and complemented strain. Mean values ± s.d. of *N* = 3 independent experiments are shown. *P*-values were calculated using two-sample *t*-test (**P<0.01; ***P<0.001). (B) Gliotoxin levels in the Δ*fbx15* strain were 3x increased compared to wild type and complemented strain. Mean values ± s.d. of *N* = 3 independent experiments are shown. *P*-values were calculated using two-sample *t*-test (**P<0.01). (C) The RFP-tagged, constantly unphosphorylated Fbx15[S468|9A] version caused increased *gli* gene expression similar to the Δ*fbx15* mutant and in contrast to the RFP-tagged wild type or Fbx15[S469D] mimicking constant phosphorylation. Mean values ± s.d. of *N* = 3 independent experiments are shown. *P*-values were calculated using two-sample *t*-test (*P<0.05; **P<0.01). (D) Increased transcription of the gene encoding dephosphorylated Fbx15[S468|9A] is independent of gliotoxin production levels, which is similar in Fbx15 wild type and both phosphomutant variants of Fbx15. Mean values ± s.d. of *N* = 3 independent experiments are shown. *P*-values were calculated using two-sample *t*-test and showed no significant difference (*P*>0.1). (E) Survival rates of immunosuppressed mice (n = 10) infected with conidia of the respective Δ*fbx* strains in comparison to mock-infected control group with PBS. Δ*fbx15*-infected mice did not show any symptoms compared to wild type and complemented strain (*P*<0.0001). The Δ*fbx23* displayed slightly increased virulence (*P* = 0.0338), whereas Δ*grrA* mutants did not show significant difference to wild type (*P*>0.05). *P*-values were determined by the Mantel-Cox log-rank test with pairwise comparison. (F) Histopathology of lung tissue from mice infected with AfS35 (WT), Δ*fbx15*, Δ*fbx15*::*fbx15* ^*+*^ and PBS-control using PAS staining. Black arrows indicate infection sites. The infection site of Δ*fbx15* was already cleared; the lung tissue of AfS35 (WT) and Δ*fbx15*::*fbx15* ^*+*^ infected mice displayed normal fungal persistence.

It was analyzed, whether Fbx15 is part of the transcriptional repression of gliotoxin synthesis genes, because SsnF, which represents a part of a general conserved transcriptional repression mechanism, is accumulated at the nuclear periphery in the Δ*fbx15* mutant. We analyzed the expression of *gliZ*, which encodes a transcriptional activator that has been shown to drive the expression of *gli*-genes encoded in the gliotoxin gene cluster and *gliP*, encoding the non-ribosomal peptide synthetase GliP with a key role in gliotoxin biosynthesis [21,23]. The expression of *gliZ* and *gliP* in the Δ*fbx15* mutant increased by almost thirteen and five times respectively in comparison to wild type or complemented strain. Gliotoxin is a toxic metabolite for *A*. *fumigatus* itself. Expression patterns of *gli* genes, which are important for detoxification mechanisms, were examined. These include *gliK*, which is required for gliotoxin biosynthesis and secretion or *gliT* encoding a oxidoreductase with the ability to reversibly form the toxic disulphide bond of gliotoxin [27–29]. Compared to wild type the *gliK* and *gliT* mRNA levels increased significantly in the Δ*fbx15* mutant by three and twelve times, respectively (Fig 6A).

Gliotoxin production of the Δ*fbx15* mutant was determined to analyze whether increased transcription of genes involved in gliotoxin production and detoxification correlates to a change in secondary metabolism. High-performance liquid chromatography (HPLC) revealed that the gliotoxin production of Δ*fbx15* was increased by 3-fold compared to wild type (Fig 6B).

The phosphomutant versions of Fbx15, which mediate different localization of SsnF in or outside the nucleus, were included into the analysis of the transcription of gliotoxin biosynthetic and protecting genes. The *fbx15* variant that produces the non-phosphorylatable Fbx15[S468|9A], which leads to cytoplasmic accumulation of SsnF, showed similarly increased *gli* gene transcript levels as the Δ*fbx15* deletion strain for *gliZ* and *gliP* and a moderately increased expression of *gliK* and *gliT*. In contrast, the Fbx15[S469D] variant mimicking constantly phosphorylation of Fbx15 and supporting nuclear localization of SsnF, resulted in a significantly lower expression of *gli* gene transcript levels as the non-phosphorylatable Fbx15[S468|9A] (Fig 6C).

Gliotoxin production levels of strains expressing either variant of unphosphorylated Fbx15[S468|9A] or phosphorylation mimicking Fbx15[S469D] were in a similar range as wild type and significantly lower than the Δ*fbx15* deletion strain (Fig 6D).

These results suggest that Fbx15 is required for repression of *gli* gene expression. The transcription of *gli* genes is derepressed and in addition the gliotoxin production is increased in the absence of Fbx15, when SsnF accumulates at the nuclear periphery. The transcription of *gli* genes is also increased in the presence of an unphosphorylatable Fbx15, which excludes SsnF from the nucleus, but gliotoxin production is not increased. This suggests an additional function of Fbx15 at a posttranscriptional layer of control for gliotoxin synthesis, which is independent of its phosphorylation status.

### Fbx15 is essential for virulence in a mouse model of aspergillosis

An established murine model of invasive pulmonary aspergillosis (IPA) was used to analyze whether Fbx15 mediated stress response and gliotoxin production control affect fungal virulence in comparison to wild type, Δ*fbx23* and Δ*grrA* strains. Immunosuppressed mice infected with wild type, Δ*fbx23*, Δ*grrA* or complemented strains displayed normal mortality rates within 14 days, although the Δ*fbx23* strain displayed a slightly increased virulence (p = 0.03) in direct comparison to the wild type. In contrast, the Δ*fbx15* mutant completely lost its virulence (Fig 6E). The Δ*fbx15*-infected mice did not show any symptoms and had the same clinical appearance as the mock infected control group, treated with phosphate buffered saline (PBS). Histopathology analyses of infected lung tissue were consistent with survival rates. Mice infected with either wild type or complemented strains showed fungal hyphae surrounded by tissue necrosis and extensive immune cell infiltration (Fig 6F). Moderate immune cell infiltrates were found in the lungs of mice infected with the Δ*fbx15* mutant, but no fungal hyphae could be detected. The fungus was cleared at an early stage of infection, probably by innate immune responses and increased temperature and elevated oxidative stress. Our data suggests that Fbx15, which is not required for growth without stress, plays a crucial role during infection because it enables *A*. *fumigatus* to adapt to innate immune response conditions of the host including limiting nutrition, fever or oxidative stress.

## Discussion

*Aspergillus fumigatus* is the most prevalent cause for pulmonary infections in immunocompromised patients. High thermo- and oxidative stress tolerance, toxic metabolites and a versatile metabolism allow *A*. *fumigatus* to colonize host tissue [30]. We identified the fungal-specific F-box protein Fbx15, which is not required for vegetative growth in the absence of stress, as key determinant for stress response, controlled gliotoxin production and virulence. A novel dual molecular function was discovered for Fbx15. Fbx15 can be part of an SCF E3 ubiquitin ligase complex and in addition controls nuclear localization of SsnF as transcriptional repressor subunit. Fbx15 levels are transcriptionally regulated and Fbx15 location in either the nucleus or the cytoplasm is determined by phosphorylation and dephosphorylation, respectively. Fbx15 is a potential target for antifungal drugs, because it is essential for *A*. *fumigatus* virulence.

**Fig 7:**
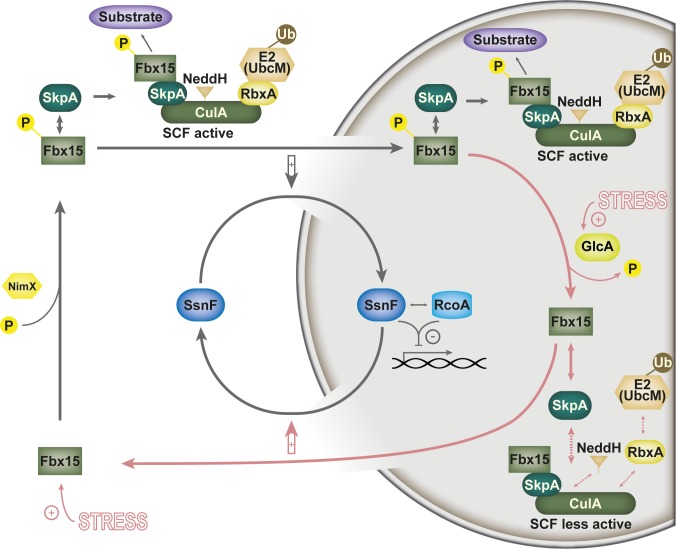
Model for Fbx15 function and localization during vegetative growth and stress response in *A*. *fumigatus*. During vegetative growth a significant part of Fbx15 is phosphorylated, potentially by cyclin-dependent kinase NimX. Phosphorylated Fbx15 is transported together with the transcriptional repressor adaptor SsnF into the nucleus. Nuclear SsnF forms a functional co-repressor complex with RcoA tetramer and interacts with DNA binding proteins for the repression of stress or secondary metabolite target genes. The small cytoplasmic and the larger nuclear fraction of phosphorylated Fbx15 can be integrated into active SCF^Fbx15^ E3 ubiquitin ligases (black arrows). Fbx15 is produced in higher levels and interacts with the phosphatase GlcA during stress conditions, resulting in dephosphorylated Fbx15 (red arrows). Dephosphorylated Fbx15 can be integrated into less active nuclear SCF complexes and leads to less nuclear SsnF. This can be caused by a reduced nuclear import/enhanced export of the co-repressor and/or a change in SsnF stability. Reduced nuclear SsnF results in derepression of stress response and secondary metabolite genes.

Our data demonstrate that Fbx15 plays a crucial role for adaptive responses to environmental changes and general stress response mechanisms in *A*. *fumigatus* whereas during non-stress conditions *fbx15* is dispensable for normal growth. This correlates with the expression patterns of *fbx15* gene transcription and translation, which demonstrated that Fbx15 becomes only abundant during oxidative stress induction. Whether this behavior is similar for different stressors remains to be shown. The low expression levels of *fbx15* during non-stress conditions are is required for the nuclear localization of the transcriptional co-repressor subunit SsnF (Fig 7). The best known example of this repressor complex, conserved in eukaryotes, is yeast Ssn6(Cyc8)-Tup1. It affects expression of at least 334 genes during normal growth conditions, which include developmental, metabolic or stress response pathways [31]. Ssn6 acts as an adaptor between a tetramer of Tup1 and additional DNA-binding proteins which mediate sequence specificity [33,35]. Tup1 alone is able to promote transcriptional repression in yeast, which might explain the ability of the *A*. *fumigatus* Δ*fbx15* mutant to grow under normal conditions, where nuclear SsnF is compromised.

The oxidative stress response gene *cat1*, which encodes a mycelial catalase, represents a target gene, which is repressed in a Fbx15-dependent way [32,43]. Expression of *cat1* is increased in the absence Fbx15, but this derepression is not sufficient for an appropriate oxidative stress response [5,44,45]. Oxidative stress results in *Aspergillus nidulans* in genome-wide transcriptional changes, rather than a specific response of distinct gene groups [7,8,46]. Derepression of *cat1* as well as of several gliotoxin biosynthetic genes correlates with the mislocalization of the nuclear repressor subunit SsnF in the cytoplasm in the absence of Fbx15. The control of nuclear SsnF localization by the phosphorylation status of Fbx15 contributes but is not the only cause of an appropriate oxidative stress response.

The F-box domain of Fbx15 is essential for an appropriate oxidative stress response. This domain serves as binding site for the assembly into SCF ubiquitin ligases. This indicates additional SCF-dependent functions for Fbx15 during oxidative stress. Disassembly of those E3 SCF ubiquitin ligases, which are not binding target substrates for ubiquitination, requires their inactivation by the COP9 signalosome CSN [3,47]. The *csn*-deficient mutant strains of *A*. *nidulans* are impaired in this control of cellular ubiquitin ligase activities and show a severe growth phenotype in the presence of oxidative stress [11,25,48,49]. Impaired CSN activity in *csn*-deficient mutant strains resulted in the enrichment, isolation and identification of SCF-Fbx15 complexes in *A*. *nidulans* [17]. This suggests that the F-box mediated assembly of SCF-Fbx15 as well as the disassembly by CSN are crucial for the function of Fbx15 during oxidative stress.

Yeast Tup1 corresponds to Aspergillus RcoA and to Groucho/TLE of higher eukaryotes. Multiple repression mechanisms are associated to these complexes such as histone deacetylation, chromatin rearrangements, modification of RNA polymerase II activity and competition with transcriptional activators [14–19,50–52]. Repression can even be changed to activation as shown for the transcriptional repressor Sko1, which inhibits hyperosmotic stress response genes in conjunction with Ssn6-Tup1. Osmotic stress leads to the phosphorylation of Sko1, which turns it into a transcriptional activator that in conjunction with Tup1 recruits chromatin-remodeling complexes such as SAGA and SWI/SNF to the respective promoter sites. ATP-driven chromatin remodeling mediated by the SWI/SNF complex is further required for cell cycle progression and activation of DNA damage repair pathways [53–55]. The lack of appropriate molecular derepression or activation of stress response genes during stress contributes to the observed serious defects in fungal growth in the *A*. *fumigatus* Δ*fbx15* mutant. There seems to be an additional posttranscriptional layer of control, which does not depend on the phosphorylation status of Fbx15. A block of nuclear SsnF in the presence of unphosphorylated Fbx15[S468|9A] results in increased transcription of *gli* genes but not increased gliotoxin production and has only a partial contribution for the resistance towards oxidative stress.

Rapid Fbx15 dephosphorylation during oxidative stress could be triggered by the essential protein phosphatase 2A catalytic subunit GlcA/BimG (Fig 7). GlcA belongs to the serine/threonine phosphatases and shares homology with the yeast protein phosphatase 1 (PP1) catalytic subunit Glc7. *GLC7* is essential for yeast as well, but conditional mutant alleles of *GLC7* could be connected to defects in adaptive functions like temperature tolerance, glucose repression, amino acid starvation, cell morphology and DNA damage repair, which are reminiscent to the growth defects of the Δ*fbx15* mutant on the respective conditions [4,22,56–59]. Dephosphorylation of Fbx15 results in nuclear clearance of SsnF. This could be due to nuclear export combined with reduced import of SsnF and/or ubiquitin dependent or even independent degradation of SsnF. Nuclear trafficking control is supported by the observation that SsnF is blocked at NPCs in an Fbx15-dependent manner upon stress. In this context a potential ubiquitinating function of Fbx15 towards the nuclear pore complex (NPC) subunit Nic96, which was co-purified during our TAP-tag pull-downs, was a reasonable function for SCF^Fbx15^-ligase complexes to promote nuclear transport control. And although the localization of SsnF-GFP in Δ*fbx15* background and the localization of Nic96-GFP shared some similarities, we could not observe an Fbx15-specific or oxidative stress-dependent ubiquitination pattern for Nic96 (Fig 5A, S8 Fig). However, the nuclear pore complex is a massive multi-protein complex composed of 30 different NPC-proteins, which are arranged in multiples and finally reach a molecular mass between 66 and 125 MDa [2,60]. In 2012 Hayakawa *et al*. showed that approximately half of the NPC-proteins in yeasts are ubiquitinated, but not necessarily targeted for proteasomal degradation [4,61]. It might be possible that Fbx15 plays a role in NPC-protein ubiquitination, which targets NPC-proteins in close proximity to Npc96 and thereby promotes a more general nuclear transport control.

Nuclear clearance of SsnF may also be triggered by selective ubiquitin-independent degradation of nuclear SsnF populations. Previous studies have shown that some E3-ubiquitin ligases directly interact with the regulatory particle of the proteasome and thus are able to transfer target proteins to the proteasome for degradation [24,25,62]. A similar scenario could be responsible for selective nuclear degradation of SsnF, where dephosphorylated Fbx15 incorporates into inactive SCF-core complexes, which have the potential to carry specific target substrates such as SsnF directly to the proteasome.

Phosphorylated or dephosphorylated Fbx15 interacts with SsnF at different cellular localizations. However, Fbx15 and SsnF are not present in stoichiometric amounts, which argues against stable Fbx15-SsnF complexes. Phosphorylated Fbx15 interacts with SsnF under non-stress conditions predominantly in the cytoplasm, suggesting a cargo function for Fbx15, which facilitates the nuclear import of Fbx15-SsnF heterodimers. Fbx15 protein levels are increased during stress and Fbx15 is dephosphorylated. Unmodified Fbx15 interacts with SsnF primarily in the nucleus and might compete with Tup1 interaction. In addition, Fbx15 possibly exhibits a nuclear export function by acting as a cargo receptor for SsnF export.

Similar to the interaction with SsnF, Fbx15 interaction with the adaptor protein SkpA, which bridges Fbx15 into SCF complexes was not disturbed due to the dephosphorylation of Fbx15. However, the interaction was shifted from cytoplasm to the nucleus. The Fbx15 phosphorylation site Ser468/469 is located between two NLSs. Therefore phosphorylation/dephosphorylation events on Fbx15 Ser468/469 might determine nuclear Fbx15 import or export by rearranging the NLS availability.

Fbx15 phosphorylation affects SsnF location. Disturbed localization patterns of SsnF in constantly unphosphorylated *fbx15* mutants resulted in moderate phenotypes, whereas the additional deletion of the F-box domain of Fbx15 led to impaired oxidative stress resistance similar to the complete *fbx15* deletion mutant. This suggests that Fbx15 as subunit of the SCF^Fbx15^ complex is an important player in the stress tolerance mechanism of *A*. *fumigatus*. This additional SCF-dependent function for Fbx15, apart from SsnF localization control, is also supported by the fact that only complete *fbx15* deletions led to increased gliotoxin levels in the mutant, whereas constantly unphosphorylated *fbx15* mutants showed increased *gli-*gene expression, which did not lead to increased gliotoxin levels. The formation of active SCF^Fbx15^ complexes was especially promoted in *fbx15* mutants, which mimic a constant phosphorylation, indicating an ubiquitinating function of phosphorylated Fbx15-carrying SCF ligases during non-stress conditions. Fbx15 abundance under non-stress conditions is very low and overall ubiquitin-patterns of the cellular pool of proteins were not significantly changed between wild type and Δ*fbx15* mutants. This suggests that putative target(s) of SCF^Fbx15^ are highly specific.

The role of F-box proteins in virulence might vary in different pathogenic fungi. *A*. *nidulans* Δ*grrA* mutants are unable to produce mature ascospores due to a block in meiosis [3,26]. We showed here that the deletion of *grrA* in *A*. *fumigatus* did not affect virulence. GrrA shares structural similarity to Fbp1 of the opportunistic human pathogen *Cryptococcus neoformans* and deletion of *fbp1* led to a loss of virulence [10,12,63]. In contrast to Fbx15, Fbp1 is not involved in a broad range of stress responses but plays a more specific role in cell membrane integrity where the loss of *fbp1* results in a block in meiosis, which finally leads to an impaired sexual sporulation, similar as GrrA in *A*. *nidulans*.

Fbx15 is a developmental regulator in the model organism *A*. *nidulans*, where the deletion of *fbx15* results in a complete block in sexual and asexual development [4,26]. The deletion of the Tup1 homolog *rcoA* in *A*. *nidulans* leads to a phenotype very similar to *A*. *nidulans* Δ*fbx15* mutant strains, which are blocked in developmental pathways and secondary metabolism [4,64]. Thus Fbx15 mediated localization control of SsnF might be a conserved mechanism in filamentous fungi. However, *fbx15* deletion mutants in the opportunistic pathogen *A*. *fumigatus* did not display a developmental defect, but Fbx15 emerges as key regulator for stress response and virulence. Several virulence factors of *A*. *fumigatus*, like mycotoxin production, oxidative stress resistance and nutritional versatility are linked to developmental control mechanisms, which were identified in the apathogenic model organism *A*. *nidulans*. This example of a connection between developmental regulators of non-pathogenic fungi and their role for virulence in fungal pathogens might be an interesting paradigm for future approaches to identify novel so far unknown virulence determinants.

*A*. *fumigatus* Fbx15 is specific to filamentous fungi, which provide the opportunity for drug design. The treatment of invasive aspergillosis is still primarily based on aggressive and toxic antifungal drugs. This disadvantage is aggravated by increasing numbers of *A*. *fumigatus* species, with resistances against commonly used medical triazoles [4,65–67]. Fbx15 might be a potential drug target, excluding the risk of cross-reactions with human proteins. In contrast to novel drugs, which target the general ubiquitin proteasomal machinery by inhibiting their core components, such as Nedd8-activating enzymes, the SCF-adaptor Skp1 or the proteasome, and thus providing therapeutic chances for cancer, neurodegenerative diseases and immune deficiencies, a drug against Fbx15 would not affect the ubiquitin-proteasome system itself, but instead offer a highly specific inhibitor for fungal dissemination during life threatening aspergillosis [5,26,68,69]. So far promising drugs, targeting specific F-box proteins have been identified for human F-box proteins, which are connected to a diverse set of cancers [70]. The possibility to treat fungal diseases with F-box specific inhibitors is supported by Lobo et al., who discovered the plant defensin *Ps*d1 from *Pisum sativum* that interacts with the nuclear F-box protein cyclin F of *N*. *crassa* and exhibits antifungal activity against several *Aspergillus* species [71]. A putative drug against Fbx15 might have a highly specific fungal target spectrum, because the function of Fbx15 varies between a stress response regulator in *A*. *fumigatus* and a developmental regulator in *A*. *nidulans*. Fbx15 might bear the potential to identify new virulence determining factors, which can be used for advanced drug design. Our identification of further Fbx15 interaction partners provides a promising base for the characterization of other novel virulence traits in *A*. *fumigatus*.

Taken together, Fbx15 is a crucial regulator for stress response and virulence in *A*. *fumigatus*, which provides the known function of an F-box protein, interacting with the ubiquitin SCF E3 ligase machinery. As a second function it controls the nuclear localization of the transcriptional co-repressor SsnF, which is part of a broad transcriptional network, including histone modifications. The broad impact of Fbx15 on stress responses and the fact that it is specific for the fungal kingdom makes this protein an interesting target for drug development.

## Materials and Methods

### Strains, media and general DNA manipulations

The generation of deletion-, complementation- and tagged strains was carried out in the Δ*akuA*-strain AfS35 a derivative of WT-strain D141, which provides high levels of homologous recombination [30,72,73]. BiFC was done in *pyrG1*-strain Af293.1 (FGSC#1137), obtained from the Fungal Genetics Stock Center [32,74]. *A*. *fumigatus* strains were cultivated in minimal media (MM) with appropriate supplements. For cloning techniques *E*. *coli* strains DH5α and MACH-1 (Invitrogen) were applied. Fungal and bacterial transformations were carried out as described [34,75]. Plasmids and *A*. *fumigatus* strains are given in Tables S4/S6 and S1 Text.

### Real-time-PCR

Total RNAs were extracted with “RNeasy plant mini kit” (Qiagen). 0.8 μg RNA was transcribed into cDNA using “QuantiTect reverse transcription kit” (Qiagen). Gene expressions were measured with quantitative real-time PCR using either a Light Cycler 2.0 System (Roche) with “RealMasterMix SYBR ROX 2.5x” (5Prime) or a CFX Connect Real-Time System (Bio-Rad) with “MESA GREEN qPCR MasterMix Plus for SYBR Assay” (Eurogentec). Histone (*h2A*) and Glyceraldehyde-3-phosphate dehydrogenase (*gpdA*) expression were used as reference for relative quantification. Details about used cDNA concentrations and primer pairs are given in S5 Table and S1 Text.

### Immunoblotting

For Immunoblotting experiments 100–150 μg crude protein extract was separated by SDS-PAGE and transferred to a nitrocellulose membrane by electro blotting as described previously [36,76]. Antibodies used for detection of fusion proteins are described in S1 Text. Signals were detected by enhanced chemiluminescence technique with either an Amersham Hyperfilm-P (GE Healthcare Limited) or with the Fusion SL7 system (Peqlab). For signal quantification Bio 1D imaging software (Peqlab) was used. Protein stability was measured by the addition of 25 μg/ml cycloheximide prior to protein extraction.

### Tandem affinity purification (TAP)

Co-purification with TAP-tagged Fbx15, Fbx15(P12S), SconB and SconB(P200S) was performed with a modified version of the Tandem Affinity Purification protocol as described in S1 Text.

### GFP-/ RFP-trap

Immunoprecipitation of GFP- or RFP-tagged proteins were performed with “GFP-trap_A and “RFP-trap_A (chromotek). Proteins were extracted from 5 ml frozen pulverized mycelium. 5 ml of protein crude extracts were incubated with 40 μl of GFP-Trap_A or RFP-Trap_A agarose, which was previously equilibrated to the B300-buffer. After two hours of incubation agarose was washed twice with B300 buffer. The agarose was boiled in 100 μl 3x SDS-sample buffer to elute the bound proteins. The extracted proteins were used directly for SDS-PAGE followed by immunoblotting or coomassie-staining and tryptic digestion for LC-MS/MS analysis.

### TMT-labeling

Fbx15-GFP was purified from cultures before and after treatment with 3 mM H_2_O_2_ and run on an SDS-PAGE. After coomassie-staining the proteins were in-gel digested with trypsin. The purified peptides were labeled with an isobaric mass tag using the “TMTduplex Isobaric Mass Tagging Kit” (Thermo Scientific), where Fbx15-GFP before H_2_O_2_ treatment was labeled with heavy TMT-127 and all time points after H_2_O_2_ induction were labeled with TMT-126 (for details see S1 Text).

### LC-MS/MS Protein Identification

Proteins were digested with “Sequencing Grade Modified Trypsin” (Promega). Digested peptides were extracted from polyacrylamide gel and separated using reversed-phase liquid chromatography with an *RSLCnano Ultimate 3000* system (Thermo Scientific) followed by mass identification with a *Orbitrap Velos Pro* mass spectrometer (Thermo Scientific). Details about mass spectrometry and data analysis are given in S1 Text.

### Murine virulence tests

The virulence of *A*. *fumigatus* Δ*fbx* mutants and the corresponding complemented strains was tested in an established murine model for IPA [37,38,77]. In brief, female CD-1 mice were immunosuppressed with cortisone acetate (25 mg/mouse intraperitoneally; Sigma-Aldrich) on days -3 and 0. Mice were anesthetized and intranasally infected with 20 μl of a fresh suspension containing 10^6^ conidia. A control group was mock infected with PBS to monitor the influence of the immunosuppression. The health status was monitored at least twice daily for 14 days and moribund animals (defined by severe dyspnoea and/or severe lethargy) were sacrificed. Infections were performed with a group of 10 mice for each tested strain. Lungs from euthanized animals were removed, and fixed in formalin and paraffin-embedded for histopathological analyses according to standard protocols [39,78].

### Ethics statement

Mice were cared for in accordance with the principles outlined by the European Convention for the Protection of Vertebrate Animals Used for Experimental and Other Scientific Purposes (European Treaty Series, no. 123; http://conventions.coe.int/Treaty/en/Treaties/Html/123). All animal experiments were in compliance with the German animal protection law and were approved by the responsible Federal State authority “Thüringer Landesamt für Verbraucherschutz” and ethics committee “Beratende Komission nach § 15 Abs. 1 Tierschutzgesetz” with the permit Reg.-Nr. 03-001/12.

## Supporting Information

S1 Fig Growth tests of *A*. *fumigatus* Δ*fbx* deletion strains under various stress conditions. (A) Overexpression of *fbx15* does not affect oxidative stress response. (B) Growth tests of the Δ*fbx15* mutant in comparison to AfS35 wild type and complemented strains on thiol oxidizing diamide or superoxide producing menadione. 5 x 10^3^ conidia of AfS35 wild type, the Δ*fbx15*-strain and the corresponding reconstructed strain were spotted on minimal medium (MM) plate, supplemented with 1 mM diamide or 7 μM Menadione sodium bisulfite as indicated and grown for four days at 37°C. (C) Δ*fbx*-strains were tested for their viability under different stress conditions. 5 x 10^3^ conidia of AfS35 wild type, the Δ*fbx*-strain and the corresponding reconstructed strain were spotted on minimal medium (MM) plate, supplemented with different stresses as indicated and grown for three days at 37°C. To investigate the effect of the particular F-box proteins on heat response plates were incubated at 42°C. (D) An increased Fbx15 protein level was observed after 40–60 min of H_2_O_2_ exposure for the complemented strain, whereas no Fbx15 could be detected in the Δ*fbx15* mutant.
(TIF)

S2 Fig Both F-box proteins Fbx15 and the essential SconB are primarily localized to the nucleus. (A) Heterokaryon rescue assay for primary Δ*sconB* transformants. Conidia of slow growing primary transformants were plated equally on non-selective MM and selective MM containing pyrithiamine. AfS35 (WT) and all transformants except for Δ*sconB-1* did not grow on selective plates indicating a spontaneously generated heterokaryon. (B) The Southern hybridization for Δ*sconB-1* mutant. On the left, a scheme for AfS35 (WT) and Δ*sconB* genomic loci with restriction enzyme sites *Nsi*I and *BspE*I used for Southern hybridization is given. On the right Southern-hybridization autoradiography for AfS35 (WT) and Δ*sconB-1* is shown. The Δ*sconB-1* mutant strain showed two bands, confirming an ectopic integration of the Δ*sconB*-cassette. (C) Phenotypical comparison of AfS35 (WT) with strains, which constitutively express GFP-fusion proteins of F-box proteins Fbx15 (AfGB32) and SconB (AfGB34), under normal and oxidative stress conditions. The expression of GFP-fusion proteins showed no altered colony morphology compared to wild type, thus confirming their functionality. (D) Fluorescence microscopy of strains expressing GFP-fusions of either Fbx15 or SconB under constitutive promoter revealed a predominantly nuclear localization for both F-box proteins. Nuclei were visualized with DAPI.
(TIF)

S3 Fig Deletion of the phosphatase encoding gene *glcA* is lethal for *A*. *fumigatus*. (A) Putative phosphorylation sites inside the primary amino acid sequence of Fbx15. Phosphorylation sites on serine, threonine and tyrosine residues were determined with NetPhos 2.0 (http://www.cbs.dtu.dk/services/NetPhos) [34,40,41]. Phosphorylation probability was provided with score-values from 0–1, whereas the cutoff value for potential phosphosites was set to 0.5. The experimental verified phosphosites at Ser469 and Ser468 with their respective score values are indicated. (B) Coomassie-stained SDS-gel of purified Fbx15-GFP before and after oxidative stress. The phosphatase GlcA was identified with LC-MS/MS for all stages of oxidative stress induction. Identified peptides for GlcA are shown in the table corresponding to the respective rectangles in the Coomassie-gel. (C) BiFC of cYFP-Fbx15 and nYFP-GlcA fusion proteins in strain AfGB123. A reconstituted YFP signal could only be observed in hyphae after treatment with H_2_O_2_. (D) Heterokaryon rescue assay for primary transformants of Δ*glcA*. From 12 primary transformants only one was able to propagate on selective medium containing hygromycin G. (E) Southern hybridization of Δ*glcA-7* mutant. In addition to the WT band for *glcA*, which complies with the restriction map shown at the left, the Δ*glcA-7* mutant showed an additional band confirming an ectopic integration of the marker cassette (indicated by an arrow).
(TIF)

S4 Fig Fbx15 interacts with SkpA primarily in the cytoplasm but is not required for overall cellular protein stability or ubiquitination. (A) BiFC of nYFP-SconB fusion proteins with cYFP-SkpA in strain AfGB45. Nuclear SconB-SkpA interaction is dominant, whereas Fbx15 and SkpA interacted more in cytoplasm than in the nucleus, which was shown by quantification of YFP-intensities according to their subcellular localization in 10 hyphae for each F-box protein. Whereas almost all interaction between SconB and SkpA took place in the nucleus (>90%), the interaction site of Fbx15 with SkpA was observed primarily in the cytoplasm (74%) with smaller fractions in the nucleus (26%). (B) 50 μg protein crude extract from AfS35 (WT), Δ*fbx15* and Δ*fbx15*::*fbx15* ^+^ before and after incubation with 3 mM H_2_O_2_, blotted to a Ponceau S-stained nitrocellulose-membrane showed no major differences in the cellular protein pattern. (C) Immunoblot of 50 µg protein crude extract from AfS35 (WT), Δ*fbx15* and Δ*fbx15*::*fbx15* ^+^ before and after induction with 3 mM H_2_O_2_ incubated with anti-ubiquitin antibody. The cellular ubiquitination-pattern was not significantly altered in the Δ*fbx15* mutant compared to AfS35 (WT) or complemented strain. Anti-tubulin antibody was used as loading control.
(TIF)

S5 Fig Fbx15 is a stable F-box protein compared to SconB. (A) Protein stability assays of GFP-tagged Fbx15 and SconB. Structure of the GFP-fusion proteins of Fbx15 and SconB are shown with their respective domains and their predicted molecular weight of 102.4 and 105 kDa. The 48 amino acid sequences of their respective F-box domains with the characteristic proline residue at position seven are highlighted. Respective strains AfGB32 and AfGB34 were incubated in MM for 18 hours and then shifted to 25 μg/ml cycloheximide containing MM for two hours. Crude protein extracts were prepared from cultures every 20 min. Immunoblottings were prepared using GFP and tubulin antibody as control. Protein stability was determined by signal quantification relatively to the tubulin-signal. Fbx15-GFP showed a higher stability compared to SconB-GFP. (B) Protein stability assay for GFP-fusions of Fbx15 (AfGB40) and SconB (AfGB42) after replacement of the conserved proline residues at position 7 of the respective F-box domains by serines. The protein stability of Fbx15 was not affected by the exchange of the conserved proline residue inside the F-box domain. In contrast, the protein stability of SconB[P200S] was drastically increased compared to wild type SconB. (C) Quantifications of protein levels from Fbx15-GFP and SconB-GFP in comparison to their proline mutant versions. Fbx15 showed high protein stability with a half-life of 90 min, which was independent of the presence or absence of the proline-residue in the F-box domain. In contrast SconB is a short-lived protein with a half-life of approximately 40 min. SconB stability was substantially increased after exchange of its conserved proline residue at position 200, leading to a half-life of more than two hours.
(TIF)

S6 Fig Fbx15 interacting proteins NimX and SsnF are essential for *A*. *fumigatus*. (A) BiFC of cYFP-Fbx15 and nYFP-NimX fusion proteins in strain AfGB124. A reconstituted YFP signal could be observed primarily in the cytoplasm. (B) Heterokaryon rescue assay for 12 primary Δ*nimX* transformants. Conidia of primary transformants were plated equally on non-selective MM and selective minimal medium containing hygromycin B. Only Δ*nimX* mutant 2 was able to grow on the selective medium, indicating that *nimX* is essential for *A*. *fumigatus*. (C) The Southern hybridization for Δ*nimX-2* mutant in comparison with the wild type verified an ectopic integration of the hygromycin B resistance cassette in addition to the wild type locus of *nimX* (indicated with an arrow). (D) Heterokaryon rescue assay for primary transformants of Δ*ssnF*. Primary transformants were equally plated on MM and selective medium containing pyrithiamin. Transformants Δ*ssnF 4–6* were still growing on selective medium and further analyzed by Southern hybridization. (E) Restriction map of AfS35 (WT) and Δ*ssnF* genomic loci with cutting sites for *EcoR*I used for Southern hybridization. Southern hybridization of Δ*ssnF* mutants 4–6 showed in addition to the wild type band at 11.5 kb a band at 5.3 kb indicating an ectopic integration of the Δ*ssnF* deletion cassette (indicated with an arrow). (F) Immunoblotting of SsnF-GFP in either wild type or Δ*fbx15* background before and after incubation with 3 mM H_2_O_2_. SsnF amount was not influenced by either treatment with H_2_O_2_ or in an Fbx15 dependent manner. (G) SsnF-GFP was purified from cultures with GFP-trap from either wild type or Δ*fbx15* background before and after H_2_O_2_-stress induction. Immunoblotting of the purified SsnF-GFP with anti ubiquitin antibody showed no Fbx15 specific ubiquitination of SsnF.
(TIF)

S7 Fig Growth tests of *A*. *fumigatus fbx15* phosphomutant strains with and without F-box domain under oxidative stress conditions. Growth tests of *A*. *fumigatus* strains expressing either wild type *fbx15*::*rfp* (AfGB98), *rfp* tagged phosphomutant versions of *fbx15* (AfGB101 and AfGB102) or corresponding *rfp*-tagged versions of *fbx15* that lack the F-box domain (AfGB125, AfGB126 and AfGB127) under oxidative stress mediated by menadione or diamide. 5 x 10^3^ conidia of the corresponding strains were spotted on minimal medium (MM) plate, supplemented with either 7 μM menadione sodium bisulfite or 1 mM diamide and grown for four days at 37°C.
(TIF)

S8 Fig Nuclear pore protein Nic96 is not influenced by Fbx15. (A) Fluorescence microscopy of Nic96-GFP in either WT or Δ*fbx15* background. Nuclei were stained with DAPI. Nic96-GFP could be detected at periphery of the nuclei independent of the presence or absence of Fbx15. (B) GFP-trap pull-down of Nic96-GFP in either WT or Δ*fbx15* background before and after H_2_O_2_-treatment followed by immunoblotting. Purified Nic96-GFP of respective conditions was incubated with anti-ubiquitin antibody, but no Fbx15 dependent ubiquitination pattern could be detected. Blotted membranes were subsequently incubated with anti-GFP antibody indicating equal amounts of purified Nic96-GFP.
(TIF)

S1 Table NCBI-accession numbers for *A*. *fumigatus* Fbx-proteins and their homologues in other species identified by NCBI-BLAST. Query coverage describes the percentage of the alignment, which covers the primary amino acid sequence of *A*. *fumigatus* F-box proteins. Identity shows the degree of similarity between the identified F-box protein compared to the respective F-box protein of *A*. *fumigatus*, which was calculated with ClustalW alignment.
(DOCX)

S2 Table Fbx15 motifs determined by comparison of *A*. *fumigatus* Fbx15 homologs. Primary amino acid sequences were compared with multiple EM for motif elicitation (MEME). Most conserved amino-acids are marked bold.
(DOCX)

S3 Table Putative interacting proteins for Fbx15 and SconB. Putative interaction partners of *A*. *fumigatus* Fbx15 and SconB, which were co-purified with TAP-tagged versions of Fbx15 and SconB in either wild type or mutated form Fbx15[P12S]/SconB[P200S]. Criteria for interacting proteins was that they had to appear at least twice in two independent purifications for each F-box protein. An exception is CulA which was found only once for SconB, indicated with an asterisk. Amount of independent co-purifications are given. Proteins, which were identified for both F-box proteins are bold.
(DOCX)

S4 Table *Aspergillus fumigatus* strains used in this study. (DOCX)

S5 Table Oligonucleotides used during this study. (DOCX)

S6 Table Plasmids constructed and used during this study. (DOCX)

S1 Text Additional Material and Methods. (DOCX)
