# Supplementary figures and images for: SCF Ubiquitin Ligase F-box Protein Fbx15 Controls Nuclear Co-repressor Localization, Stress Response and Virulence of the Human Pathogen Aspergillus fumigatus
Source: PLoS Pathog. 2016 Sep 20;12(9):e1005899. doi: 10.1371/journal.ppat.1005899 (PMC5029927; doi:10.1371/journal.ppat.1005899)

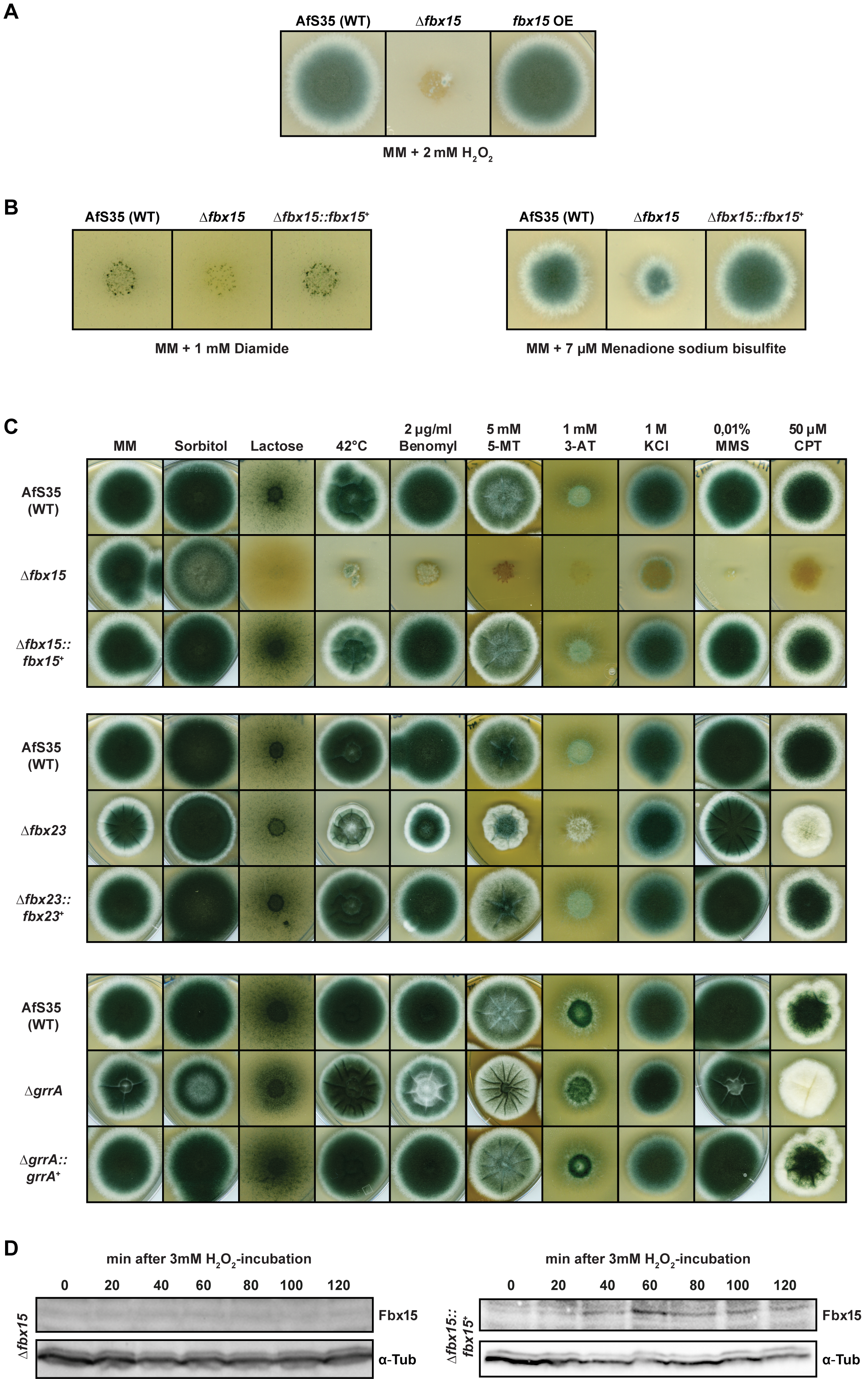

Supplement: S1 Fig — (A) Overexpression of fbx15 does not affect oxidative stress response. (B) Growth tests of the Δfbx15 mutant in comparison to AfS35 wild type and complemented strains on thiol oxidizing diamide or superoxide producing menadione. 5 x 10^3^ conidia of AfS35 wild type, the Δfbx15-strain and the corresponding reconstructed strain were spotted on minimal medium (MM) plate, supplemented with 1 mM diamide or 7 μM Menadione sodium bisulfite as indicated and grown for four days at 37°C. (C) Δfbx-strains were tested for their viability under different stress conditions. 5 x 10^3^ conidia of AfS35 wild type, the Δfbx-strain and the corresponding reconstructed strain were spotted on minimal medium (MM) plate, supplemented with different stresses as indicated and grown for three days at 37°C. To investigate the effect of the particular F-box proteins on heat response plates were incubated at 42°C. (D) An increased Fbx15 protein level was observed after 40–60 min of H2O2 exposure for the complemented strain, whereas no Fbx15 could be detected in the Δfbx15 mutant. (TIF) [file ppat.1005899.s001.tif]

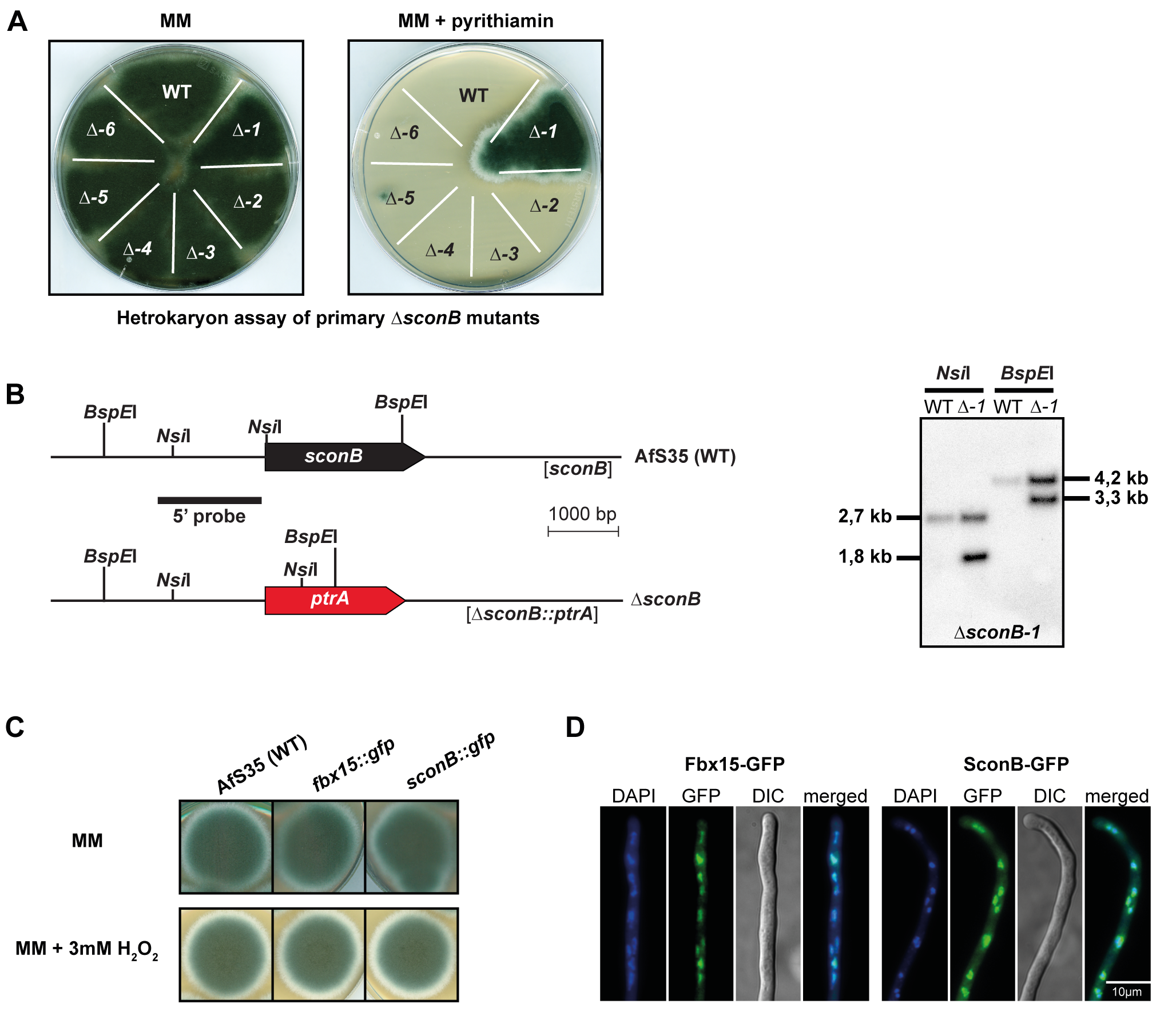

Supplement: S2 Fig — (A) Heterokaryon rescue assay for primary ΔsconB transformants. Conidia of slow growing primary transformants were plated equally on non-selective MM and selective MM containing pyrithiamine. AfS35 (WT) and all transformants except for ΔsconB-1 did not grow on selective plates indicating a spontaneously generated heterokaryon. (B) The Southern hybridization for ΔsconB-1 mutant. On the left, a scheme for AfS35 (WT) and ΔsconB genomic loci with restriction enzyme sites NsiI and BspEI used for Southern hybridization is given. On the right Southern-hybridization autoradiography for AfS35 (WT) and ΔsconB-1 is shown. The ΔsconB-1 mutant strain showed two bands, confirming an ectopic integration of the ΔsconB-cassette. (C) Phenotypical comparison of AfS35 (WT) with strains, which constitutively express GFP-fusion proteins of F-box proteins Fbx15 (AfGB32) and SconB (AfGB34), under normal and oxidative stress conditions. The expression of GFP-fusion proteins showed no altered colony morphology compared to wild type, thus confirming their functionality. (D) Fluorescence microscopy of strains expressing GFP-fusions of either Fbx15 or SconB under constitutive promoter revealed a predominantly nuclear localization for both F-box proteins. Nuclei were visualized with DAPI. (TIF) [file ppat.1005899.s002.tif]

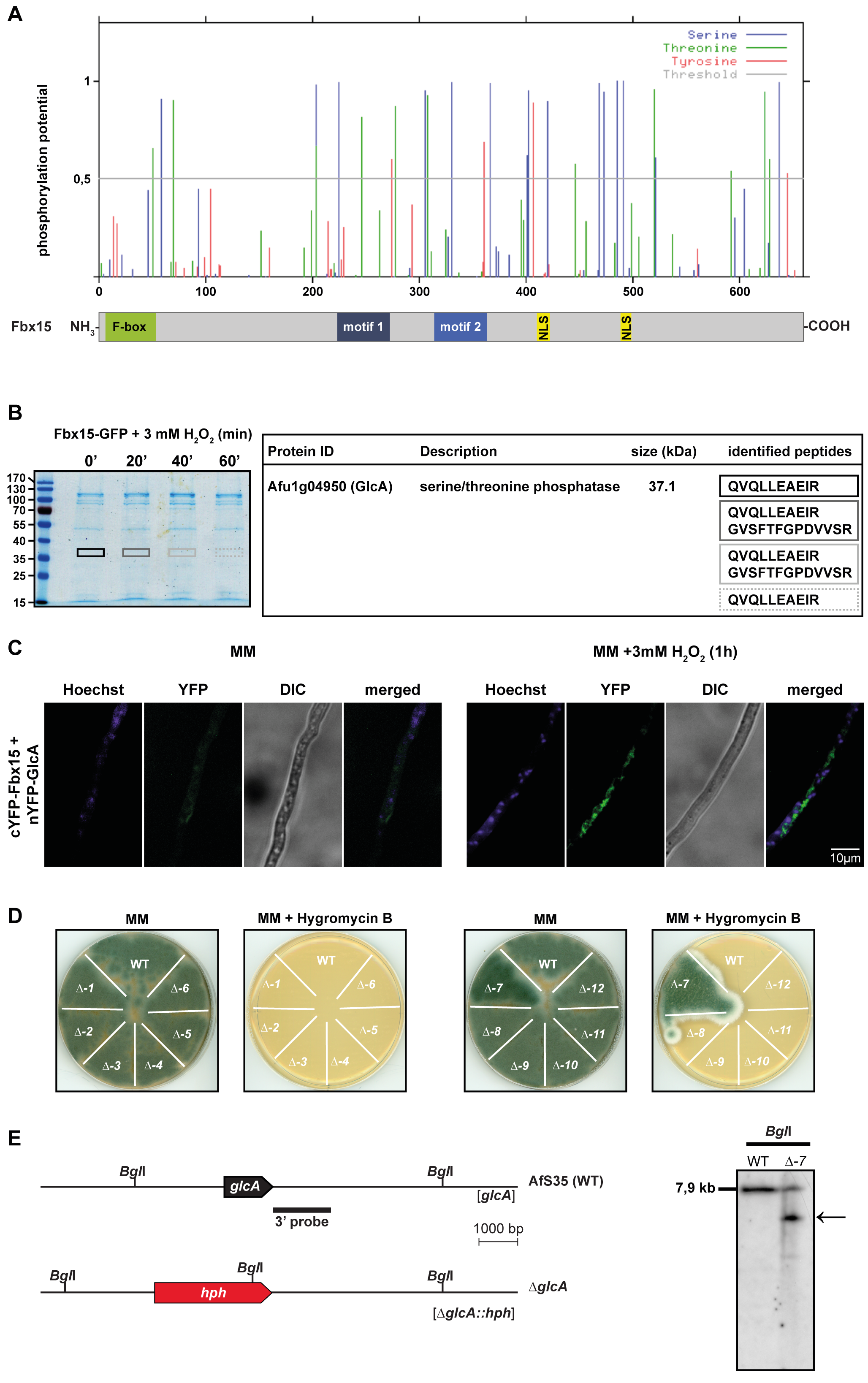

Supplement: S3 Fig — (A) Putative phosphorylation sites inside the primary amino acid sequence of Fbx15. Phosphorylation sites on serine, threonine and tyrosine residues were determined with NetPhos 2.0 (http://www.cbs.dtu.dk/services/NetPhos) [34,40,41]. Phosphorylation probability was provided with score-values from 0–1, whereas the cutoff value for potential phosphosites was set to 0.5. The experimental verified phosphosites at Ser469 and Ser468 with their respective score values are indicated. (B) Coomassie-stained SDS-gel of purified Fbx15-GFP before and after oxidative stress. The phosphatase GlcA was identified with LC-MS/MS for all stages of oxidative stress induction. Identified peptides for GlcA are shown in the table corresponding to the respective rectangles in the Coomassie-gel. (C) BiFC of cYFP-Fbx15 and nYFP-GlcA fusion proteins in strain AfGB123. A reconstituted YFP signal could only be observed in hyphae after treatment with H2O2. (D) Heterokaryon rescue assay for primary transformants of ΔglcA. From 12 primary transformants only one was able to propagate on selective medium containing hygromycin G. (E) Southern hybridization of ΔglcA-7 mutant. In addition to the WT band for glcA, which complies with the restriction map shown at the left, the ΔglcA-7 mutant showed an additional band confirming an ectopic integration of the marker cassette (indicated by an arrow). (TIF) [file ppat.1005899.s003.tif]

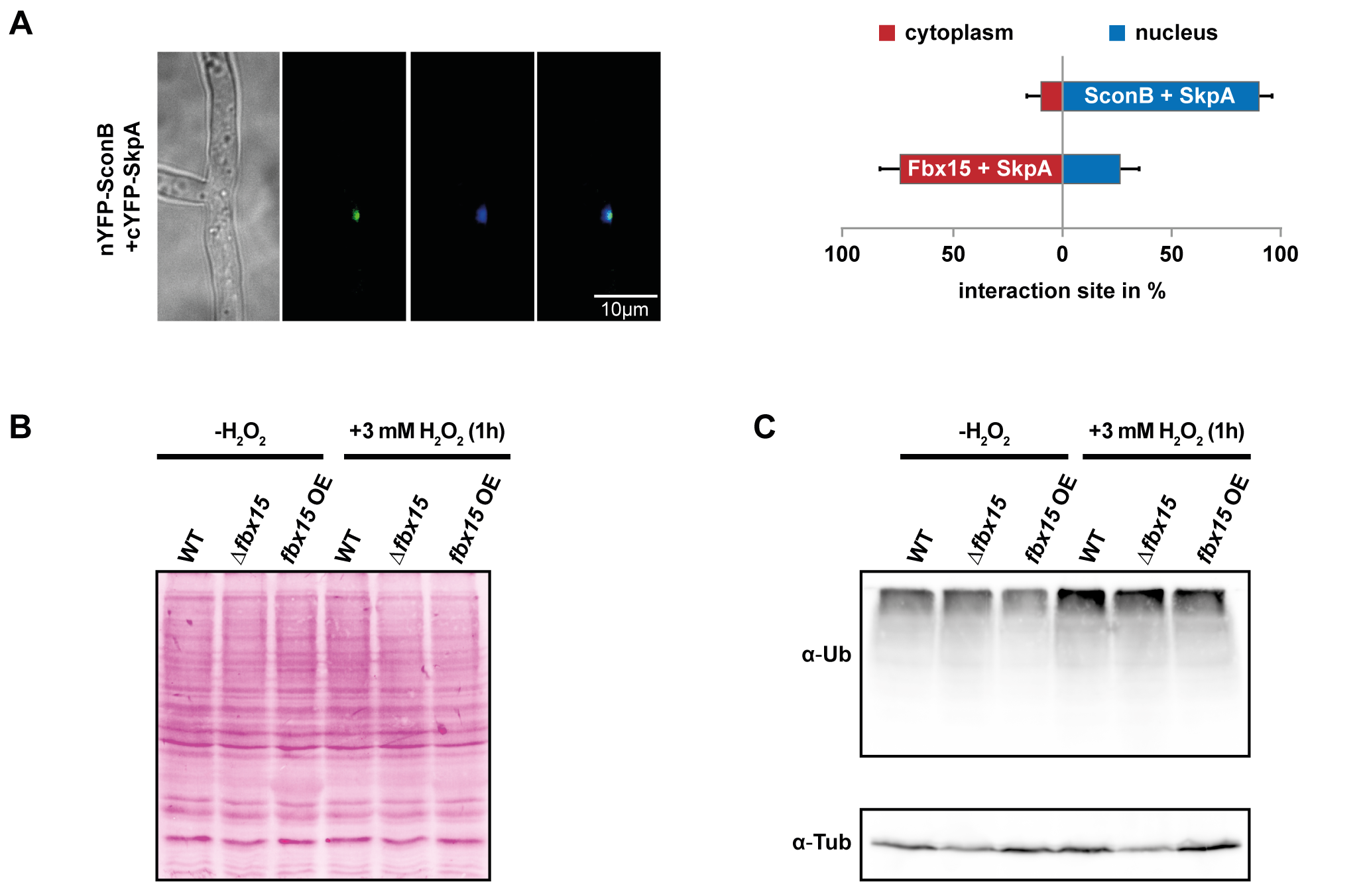

Supplement: S4 Fig — (A) BiFC of nYFP-SconB fusion proteins with cYFP-SkpA in strain AfGB45. Nuclear SconB-SkpA interaction is dominant, whereas Fbx15 and SkpA interacted more in cytoplasm than in the nucleus, which was shown by quantification of YFP-intensities according to their subcellular localization in 10 hyphae for each F-box protein. Whereas almost all interaction between SconB and SkpA took place in the nucleus (>90%), the interaction site of Fbx15 with SkpA was observed primarily in the cytoplasm (74%) with smaller fractions in the nucleus (26%). (B) 50 μg protein crude extract from AfS35 (WT), Δfbx15 and Δfbx15::fbx15 + before and after incubation with 3 mM H2O2, blotted to a Ponceau S-stained nitrocellulose-membrane showed no major differences in the cellular protein pattern. (C) Immunoblot of 50 µg protein crude extract from AfS35 (WT), Δfbx15 and Δfbx15::fbx15 + before and after induction with 3 mM H2O2 incubated with anti-ubiquitin antibody. The cellular ubiquitination-pattern was not significantly altered in the Δfbx15 mutant compared to AfS35 (WT) or complemented strain. Anti-tubulin antibody was used as loading control. (TIF) [file ppat.1005899.s004.tif]

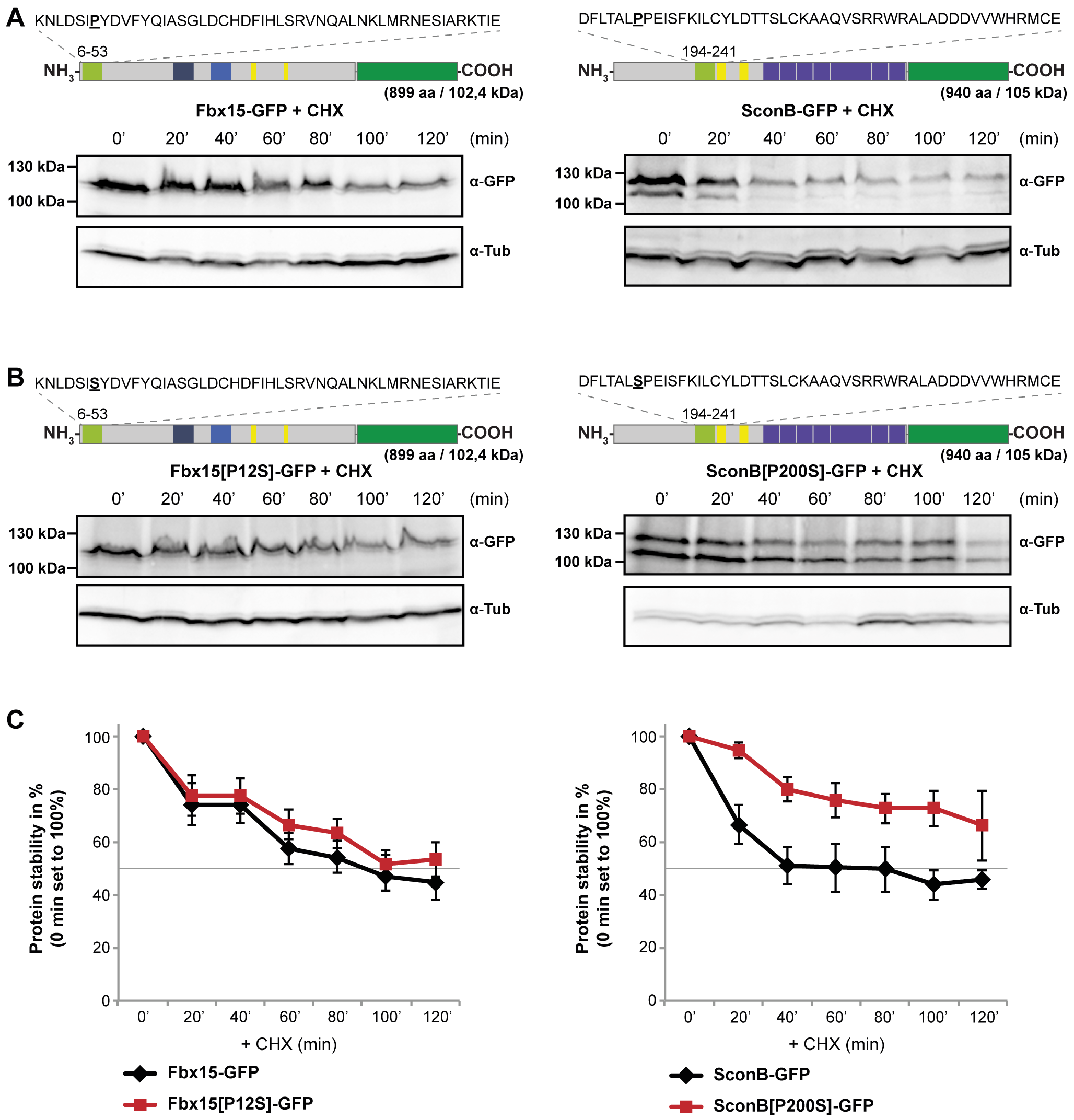

Supplement: S5 Fig — (A) Protein stability assays of GFP-tagged Fbx15 and SconB. Structure of the GFP-fusion proteins of Fbx15 and SconB are shown with their respective domains and their predicted molecular weight of 102.4 and 105 kDa. The 48 amino acid sequences of their respective F-box domains with the characteristic proline residue at position seven are highlighted. Respective strains AfGB32 and AfGB34 were incubated in MM for 18 hours and then shifted to 25 μg/ml cycloheximide containing MM for two hours. Crude protein extracts were prepared from cultures every 20 min. Immunoblottings were prepared using GFP and tubulin antibody as control. Protein stability was determined by signal quantification relatively to the tubulin-signal. Fbx15-GFP showed a higher stability compared to SconB-GFP. (B) Protein stability assay for GFP-fusions of Fbx15 (AfGB40) and SconB (AfGB42) after replacement of the conserved proline residues at position 7 of the respective F-box domains by serines. The protein stability of Fbx15 was not affected by the exchange of the conserved proline residue inside the F-box domain. In contrast, the protein stability of SconB[P200S] was drastically increased compared to wild type SconB. (C) Quantifications of protein levels from Fbx15-GFP and SconB-GFP in comparison to their proline mutant versions. Fbx15 showed high protein stability with a half-life of 90 min, which was independent of the presence or absence of the proline-residue in the F-box domain. In contrast SconB is a short-lived protein with a half-life of approximately 40 min. SconB stability was substantially increased after exchange of its conserved proline residue at position 200, leading to a half-life of more than two hours. (TIF) [file ppat.1005899.s005.tif]

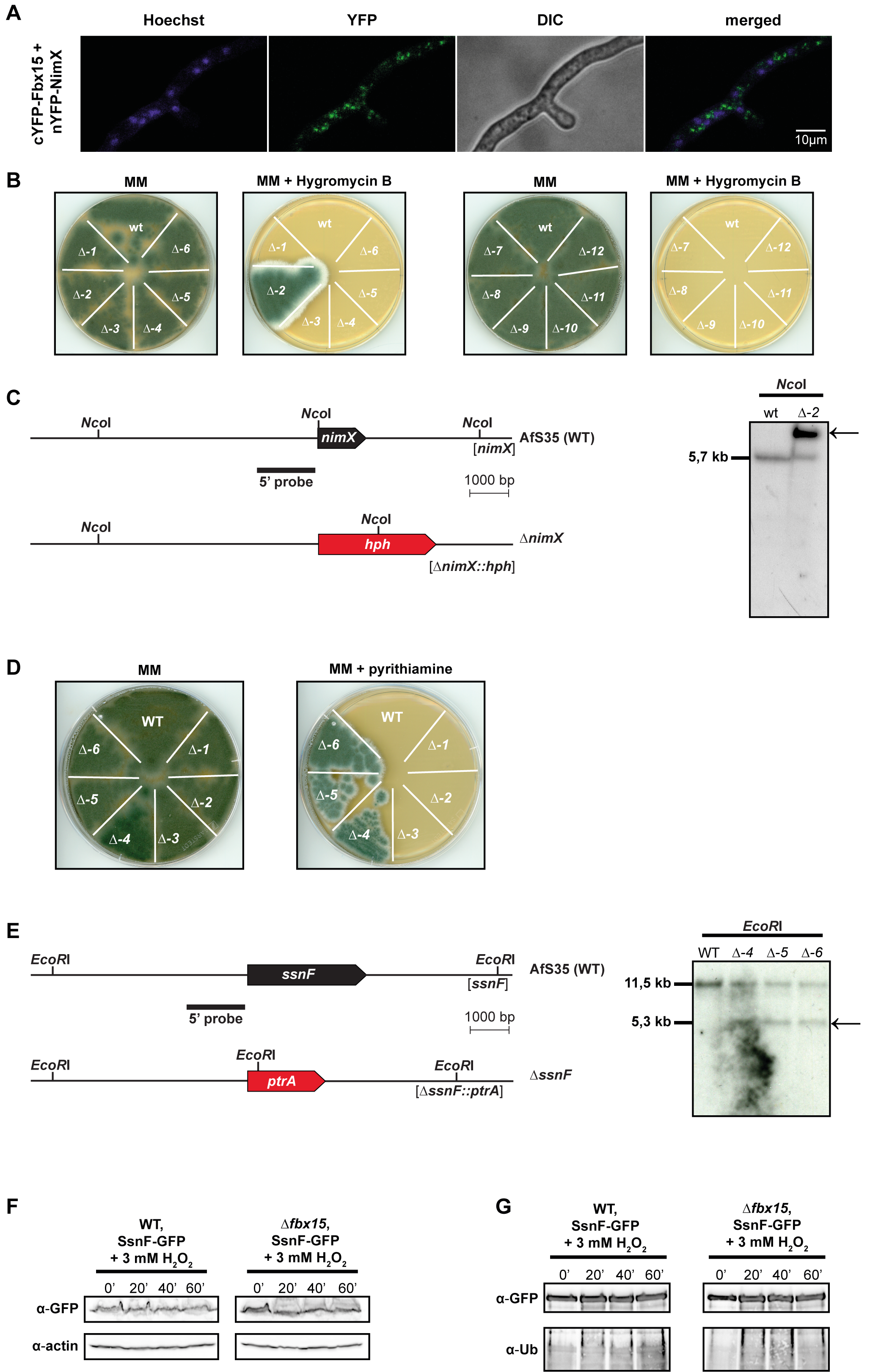

Supplement: S6 Fig — (A) BiFC of cYFP-Fbx15 and nYFP-NimX fusion proteins in strain AfGB124. A reconstituted YFP signal could be observed primarily in the cytoplasm. (B) Heterokaryon rescue assay for 12 primary ΔnimX transformants. Conidia of primary transformants were plated equally on non-selective MM and selective minimal medium containing hygromycin B. Only ΔnimX mutant 2 was able to grow on the selective medium, indicating that nimX is essential for A. fumigatus. (C) The Southern hybridization for ΔnimX-2 mutant in comparison with the wild type verified an ectopic integration of the hygromycin B resistance cassette in addition to the wild type locus of nimX (indicated with an arrow). (D) Heterokaryon rescue assay for primary transformants of ΔssnF. Primary transformants were equally plated on MM and selective medium containing pyrithiamin. Transformants ΔssnF 4–6 were still growing on selective medium and further analyzed by Southern hybridization. (E) Restriction map of AfS35 (WT) and ΔssnF genomic loci with cutting sites for EcoRI used for Southern hybridization. Southern hybridization of ΔssnF mutants 4–6 showed in addition to the wild type band at 11.5 kb a band at 5.3 kb indicating an ectopic integration of the ΔssnF deletion cassette (indicated with an arrow). (F) Immunoblotting of SsnF-GFP in either wild type or Δfbx15 background before and after incubation with 3 mM H2O2. SsnF amount was not influenced by either treatment with H2O2 or in an Fbx15 dependent manner. (G) SsnF-GFP was purified from cultures with GFP-trap from either wild type or Δfbx15 background before and after H2O2-stress induction. Immunoblotting of the purified SsnF-GFP with anti ubiquitin antibody showed no Fbx15 specific ubiquitination of SsnF. (TIF) [file ppat.1005899.s006.tif]

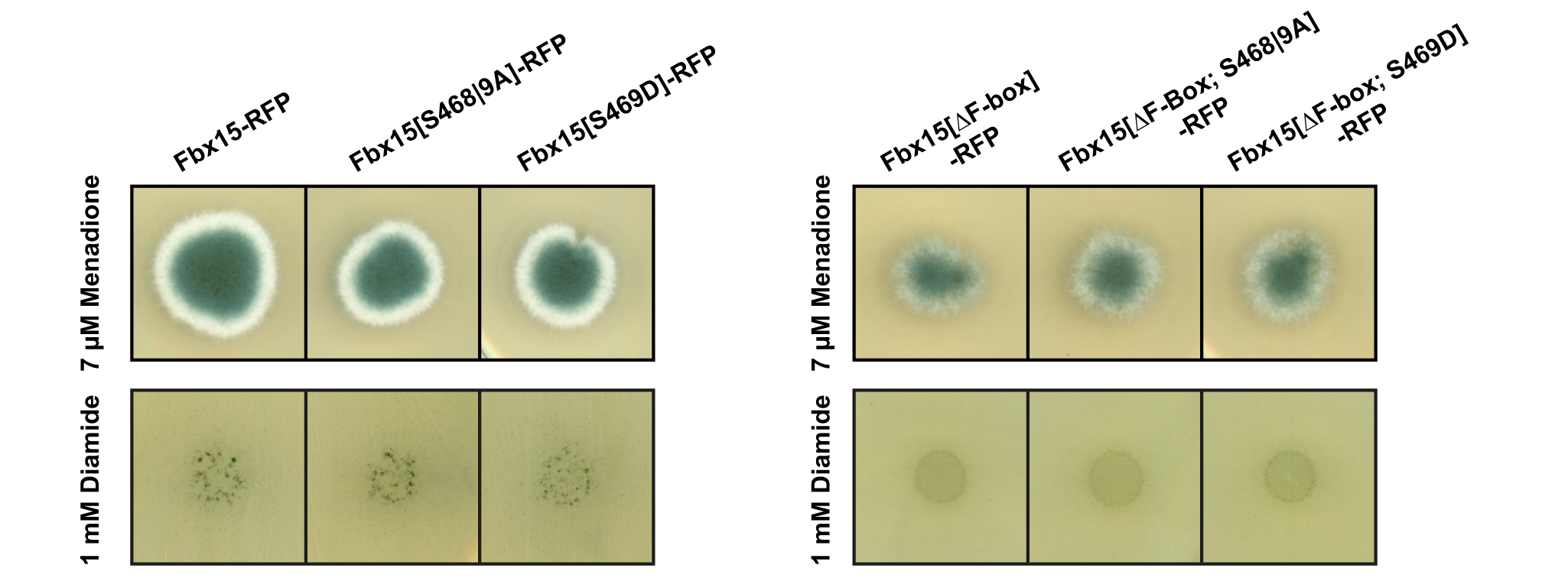

Supplement: S7 Fig — Growth tests of A. fumigatus strains expressing either wild type fbx15::rfp (AfGB98), rfp tagged phosphomutant versions of fbx15 (AfGB101 and AfGB102) or corresponding rfp-tagged versions of fbx15 that lack the F-box domain (AfGB125, AfGB126 and AfGB127) under oxidative stress mediated by menadione or diamide. 5 x 10^3^ conidia of the corresponding strains were spotted on minimal medium (MM) plate, supplemented with either 7 μM menadione sodium bisulfite or 1 mM diamide and grown for four days at 37°C. (TIF) [file ppat.1005899.s007.tif]

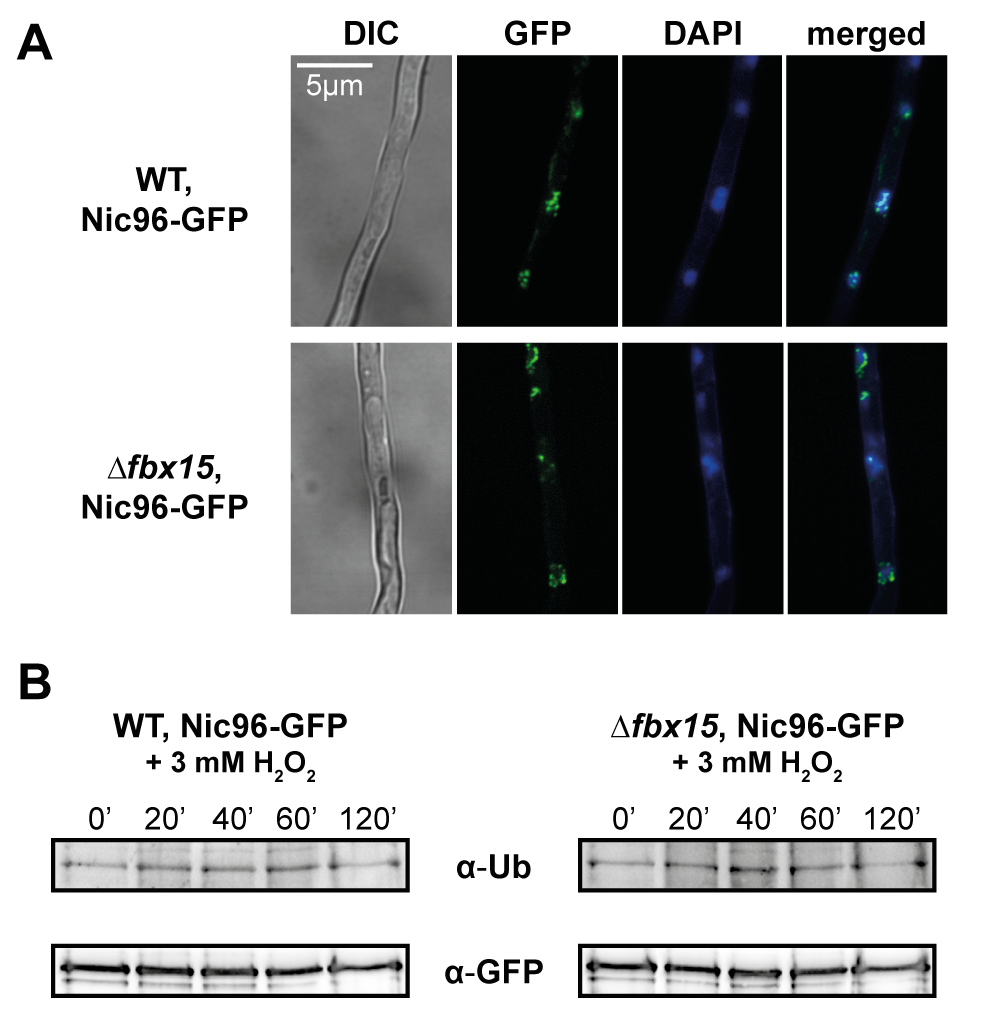

Supplement: S8 Fig — (A) Fluorescence microscopy of Nic96-GFP in either WT or Δfbx15 background. Nuclei were stained with DAPI. Nic96-GFP could be detected at periphery of the nuclei independent of the presence or absence of Fbx15. (B) GFP-trap pull-down of Nic96-GFP in either WT or Δfbx15 background before and after H2O2-treatment followed by immunoblotting. Purified Nic96-GFP of respective conditions was incubated with anti-ubiquitin antibody, but no Fbx15 dependent ubiquitination pattern could be detected. Blotted membranes were subsequently incubated with anti-GFP antibody indicating equal amounts of purified Nic96-GFP. (TIF) [file ppat.1005899.s008.tif]
