# Supplementary material for: SCF Ubiquitin Ligase F-box Protein Fbx15 Controls Nuclear Co-repressor Localization, Stress Response and Virulence of the Human Pathogen Aspergillus fumigatus
Source: PLoS Pathog. 2016 Sep 20;12(9):e1005899. doi: 10.1371/journal.ppat.1005899 (PMC5029927; doi:10.1371/journal.ppat.1005899)
Supplement: S1 Table — Query coverage describes the percentage of the alignment, which covers the primary amino acid sequence of A. fumigatus F-box proteins. Identity shows the degree of similarity between the identified F-box protein compared to the respective F-box protein of A. fumigatus, which was calculated with ClustalW alignment. (DOCX) [file ppat.1005899.s009.docx]

**Table S1. NCBI-accession numbers for *A. fumigatus* Fbx-proteins and their homologues in other species identified by NCBI-BLAST.** Query coverage describes the percentage of the alignment, which covers the primary amino acid sequence of *A. fumigatus* F-box proteins. Identity shows the degree of similarity between the identified F-box protein compared to the respective F-box protein of *A. fumigatus*, which was calculated with ClustalW alignment.

| **F-box protein** | **Organism** | **Accession#** | **Query coverage** | **Identity** |
| --- | --- | --- | --- | --- |
|  |  |  |  |  |
| **Fbx15** | ***A. fumigatus* Af293** | XP_754210.1 | **100%** | **100%** |
|  | ***A. clavatus* NRRL 1** | XP_001271331.1 | **99%** | **72.8%** |
|  | ***A. terreus* NIH2624** | XP_001210996.1 | **100%** | **69.8%** |
|  | ***A. flavus* NRRL3357** | XP_002375930.1 | **100%** | **64.5%** |
|  | ***A. oryzae* RIB40** | XP_001727634.1 | **100%** | **64.5%** |
|  | ***A. niger* CBS 513.88** | XP_001393757.1 | **99%** | **64.1%** |
|  | ***A. nidulans* FGSC A4** | XP_660109.1 | **88%** | **59.8%** |
|  | ***P. chrysogenum* Wisconsin 54-1255** | XP_002559957.1 | **99%** | **43.6%** |
|  | ***N. crassa* OR74A** | XP_958675.2 | **88%** | **24.7%** |
|  |  |  |  |  |
| **Fbx23** | ***A. fumigatus* Af293** | XP_751686.1 | **100%** | **100%** |
|  | ***A. clavatus* NRRL 1** | XP_001272005.1 | **100%** | **75.6%** |
|  | ***A. niger* CBS 513.88** | XP_001401882.2 | **79%** | **65.5%** |
|  | ***A. nidulans* FGSC A4** | XP_663197.1 | **100%** | **65.1%** |
|  | ***A. terreus* NIH2624** | XP_001213115.1 | **87%** | **64.1%** |
|  | ***A. oryzae* RIB40** | XP_001820179.2 | **69%** | **63.7%** |
|  | ***A. flavus* NRRL3357** | XP_002374252.1 | **82%** | **62.0%** |
|  | ***P. chrysogenum* Wisconsin 54-1255** | XP_002564622.1 | **90%** | **58.1%** |
|  | ***M. musculus*** | XP_006526676.1 | **47%** | **29.7%** |
|  | ***H. sapiens*** | XP_011538622.1 | **47%** | **29.7%** |
|  |  |  |  |  |
| **GrrA** | ***A. fumigatus* Af293** | XP_750347.1 | **100%** | **100%** |
|  | ***A. clavatus* NRRL 1** | XP_001269564.1 | **100%** | **89.1%** |
|  | ***A. flavus* NRRL3357** | XP_002383125.1 | **97%** | **83.0%** |
|  | ***A. oryzae* RIB40** | XP_001816943.2 | **97%** | **82.3%** |
|  | ***A. niger* CBS 513.88** | XP_001398838.2 | **100%** | **80.5%** |
|  | ***A. terreus* NIH2624** | XP_001212207.1 | **99%** | **80.3%** |
|  | ***A. nidulans* FGSC A4** | ABC25061.1 | **100%** | **78.3%** |
|  | ***P. chrysogenum* Wisconsin 54-1255** | XP_002558634.1 | **99%** | **76.6%** |
|  | ***N. crassa* OR74A** | XP_961582.3 | **86%** | **52.9%** |
|  | ***S. cerevisiae* S288c** | NP_012623.1 | **81%** | **37.0%** |
|  | ***H. sapiens*** | XP_011523694.1 | **77%** | **26.6%** |
|  | ***M. musculus*** | XP_006534361.1 | **77%** | **25.6%** |
|  |  |  |  |  |
| **SconB** | ***A. fumigatus* Af293** | XP_755744.1 | **100%** | **100%** |
|  | ***A. clavatus* NRRL 1** | XP_001275741.1 | **100%** | **83.2%** |
|  | ***A. terreus* NIH2624** | XP_001208767.1 | **94%** | **80.9%** |
|  | ***A. niger* CBS 513.88** | XP_001399618.2 | **94%** | **79.5%** |
|  | ***A. flavus* NRRL3357** | XP_002379815.1 | **100%** | **78.8%** |
|  | ***A. oryzae* RIB40** | XP_001821629.1 | **93%** | **78.7%** |
|  | ***A. nidulans* FGSC A4** | XP_663963.1 | **99%** | **77.6%** |
|  | ***P. chrysogenum* Wisconsin 54-1255** | XP_002558093.1 | **89%** | **62.3%** |
|  | ***N. crassa* OR74A** | XP_962825.2 | **91%** | **50.4%** |
|  | ***S. cerevisiae* S288c** | NP_012218.1 | **84%** | **42.2%** |
|  | ***M. musculus*** | XP_006501721.1 | **78%** | **29.2%** |
|  | ***H. sapiens*** | XP_011530388.1 | **78%** | **27.6%** |
