# Supplementary material for: SCF Ubiquitin Ligase F-box Protein Fbx15 Controls Nuclear Co-repressor Localization, Stress Response and Virulence of the Human Pathogen Aspergillus fumigatus
Source: PLoS Pathog. 2016 Sep 20;12(9):e1005899. doi: 10.1371/journal.ppat.1005899 (PMC5029927; doi:10.1371/journal.ppat.1005899)
Supplement: S2 Table — Primary amino acid sequences were compared with multiple EM for motif elicitation (MEME). Most conserved amino-acids are marked bold. (DOCX) [file ppat.1005899.s010.docx]

**Table S2. Fbx15 motifs determined by comparison of *A. fumigatus* Fbx15 homologs.** Primary amino acid sequences were compared with multiple EM for motif elicitation (MEME). Most conserved amino-acids are marked bold.

| **Fbx15 motif** | **Organism** | **Accession#** | **p-value** | **amino acid sequence (conserved residues marked bold)** |
| --- | --- | --- | --- | --- |
|  |  |  |  |  |
| **Fbx15 motif 1** | ***A. fumigatus*** | XP_754210 | 8.31e-59 | **A**LSHY**GY**P**QWAV**H**CV**N**LATG**Q**H**I**TEKPV**E**L**HN**F**V**GNEIGQTVCFEVHQDH** |
|  | ***A. oryzae*** | XP_001727634 | 2.53e-57 | GA**G**Y**HGY**K**QW**I**V**N**C**FE**LATG**KQTN**EKPV**Q**L**HD**FAGNEIGQTVCF**D**VHQDH** |
|  | ***A. flavus*** | XP_002375930 | 2.53e-57 | GA**G**Y**HGY**K**QW**I**V**N**C**FE**LATG**KQTN**EKPV**Q**L**HD**FAGNEIGQTVCF**D**VHQDH** |
|  | ***A. terreus*** | XP_001210996 | 1.45e-56 | **A**L**G**Y**HGY**P**QWAV**N**CV**D**L**T**TG**Q**H**L**T**D**KPV**E**L**EN**FAG**Y**EIGQTVCFEVH**E**DH** |
|  | ***A. clavatus*** | XP_001271331 | 1.38e-54 | **A**LSH**HGY**P**QWA**IH**CV**D**LATG**R**H**V**T**D**KPV**E**L**EKLV**GNEIGQTVCFEV**Y**QDH** |
|  | ***A. niger*** | XP_0013937575 | 3.07e-52 | **A**M**G**DR**GY**S**QWA**IH**CV**D**L**K**T**SQ**H**M**TEKP**TV**L**EN**FAG**S**EIGQTVCFEVHQDH** |
|  |  |  |  |  |
| **Fbx15 motif**  **2** | ***A. nidulans*** | XP_660109 | 1.19e-61 | **WRRQHREGPIN**DT**WSD**LSL**R**E**DEAT**GQLM**ILECRREW**RD**G**G**S**E**N**C**RTYY**V |
|  | ***A. fumigatus*** | XP_754210 | 6.08e-60 | **WRRQHREGPIN**DT**WSD**LSL**R**E**DEAT**NQLL**ILECRREW**RN**G**G**S**D**N**C**RTYY**M |
|  | ***A. terreus*** | XP_001210996 | 2.89e-59 | **WRRQHREGPIN**DT**WSD**LSL**R**H**DEAT**KRLM**ILECRREW**LD**G**G**S**D**N**C**RTYY**T |
|  | ***A. oryzae*** | XP_001727634 | 3.31e-59 | **WRRQHREGPIN**DS**WSD**ISL**R**H**DEAT**KQLI**ILECRREW**HK**G**G**S**E**N**F**RTYY**V |
|  | ***A. flavus*** | XP_002375930 | 3.31e-59 | **WRRQHREGPIN**DS**WSD**ISL**R**H**DEAT**KQLI**ILECRREW**HK**G**G**S**E**N**F**RTYY**V |
|  | ***A. clavatus*** | XP_001271331 | 3.79e-59 | **WRRQHREGPIN**ES**WSD**LSL**R**V**DEAT**NQLM**ILECRREW**RN**G**G**S**D**N**C**RTYY**M |
|  | ***A. niger*** | XP_0013937575 | 7.98e-57 | **WRRQHREGPIN**DT**WSD**LTL**R**Q**DEAT**KRLM**ILECRREW**RD**G**R**S**E**N**A**RTYY**M |
|  | ***P. chrysogenum*** | XP_002559957 | 8.94e-48 | **WRR**E**H**Q**EGPIN**EM**W**T**D**LSI**R**K**DE**T**T**GRPV**ILECRREW**RD**G**K**S**E**N**H**RTYY**T |
|  | ***N. crassa*** | XP_958675 | 2.33e-32 | L**RR**N**H**LD**GP**MDHR**W**TFMKMFK**DEAT**GEIKVV**E**S**RREW**IK**G**S**S**STT**RTYY**T |
