# Supplementary material for: SCF Ubiquitin Ligase F-box Protein Fbx15 Controls Nuclear Co-repressor Localization, Stress Response and Virulence of the Human Pathogen Aspergillus fumigatus
Source: PLoS Pathog. 2016 Sep 20;12(9):e1005899. doi: 10.1371/journal.ppat.1005899 (PMC5029927; doi:10.1371/journal.ppat.1005899)
Supplement: S3 Table — Putative interaction partners of A. fumigatus Fbx15 and SconB, which were co-purified with TAP-tagged versions of Fbx15 and SconB in either wild type or mutated form Fbx15[P12S]/SconB[P200S]. Criteria for interacting proteins was that they had to appear at least twice in two independent purifications for each F-box protein. An exception is CulA which was found only once for SconB, indicated with an asterisk. Amount of independent co-purifications are given. Proteins, which were identified for both F-box proteins are bold. (DOCX) [file ppat.1005899.s011.docx]

**Table S3. Putative interacting proteins for Fbx15 and SconB.** Putative interaction partners of *A. fumigatus* Fbx15 and SconB, which were co-purified with TAP-tagged versions of Fbx15 and SconB in either wild type or mutated form Fbx15[P12S]/SconB[P200S]. Criteria for interacting proteins was that they had to appear at least twice in two independent purifications for each F-box protein. An exception is CulA which was found only once for SconB, indicated with an asterisk. Amount of independent co-purifications are given. Proteins, which were identified for both F-box proteins are bold.

| ***A. fumigatus*** | **Protein description** | **Co-purified with:** | | | |
| --- | --- | --- | --- | --- | --- |
|  |  | **Fbx15** | **Fbx15 [P12S]** | **SconB** | **SconB [P200S]** |
| **SCF-subunits & related proteins** | |  |  |  |  |
| **AFUA_1G12960 (CulA)** | **SCF ubiquitin ligase subunit** | **3** | **3** | **1*** | **-** |
| **AFUA_5G06060 (SkpA)** | **SCF ubiquitin ligase subunit** | **3** | **2** | **2** | **2** |
| **AFUA_4G10350 (UbiD)** | **Polyubiquitin** | **1** | **2** | **1** | **1** |
| **AFUA_4G10780 (Tom1)** | **ubiquitin-protein ligase** | **1** | **1** | **1** | **1** |
| AFUA_8G05500 (CsnD) | COP9 signalosome subunit | 3 | - | - | - |
| AFUA_5G07260 (CsnF) | COP9 signalosome subunit | 2 | - | - | - |
| AFUA_4G12630 (CsnG) | COP9 signalosome subunit | 3 | - | - | - |
| **Transcription factors & nuclear proteins** | |  |  |  |  |
| AFUA_3G09670 (OefC) | C6 transcription factor | 1 | 1 | - | - |
| AFUA_4G03460 (SrbB) | bHLH transcription factor, involved in hypoxia and virulence | 1 | 1 | - | - |
| AFUA_4G04720 (Nic96) | nuclear pore protein | 0 | 2 | - | - |
| AFUA_4G08930 | Putative nucleolar GTPase (Nog2p) | 1 | 1 | - | - |
| AFUA_2G11840 (SsnF) | Transcriptional corepressor (Ssn6p) | 1 | 1 | - | - |
| **AFUA_6G05150 (RcoA)** | **Transcriptional corepressor (Tup1p)** | **1** | **1** | **-** | **2** |
| **AFUA_5G11390** | **APSES transcription factor, putative** | **2** | **3** | **1** | **2** |
| **AFUA_2G06140** | **uracil DNA N-glycosylase activity, DNA repair** | **1** | **1** | **1** | **1** |
| **AFUA_5G07890** | **single-stranded DNA binding protein (Rim1p)** | **3** | **1** | **1** | **1** |
| AFUA_4G06530 (MetR) | bZIP transcription factor (Met4p) | - | - | - | 2 |
| AFUA_6G09930 (Yap1) | bZIP transcription factor, putative (Yap1p) | - | - | 1 | 2 |
| **RNA processing** | |  |  |  |  |
| AFUA_3G06440 | Splicing factor with U2 snRNP localization (Prp21p) | 1 | 2 | - | - |
| AFUA_5G04420 | Splicing factor with U2 snRNP localization (Cus1p) | 1 | 1 | - | - |
| AFUA_6G08610 | RNA trimethylguanosine synthase, role in 7-methylguanosine cap hypermethylation (Tgs1p) | 2 | - | - | - |
| AFUA_5G09670 | RNase III domain protein | 1 | 1 | - | - |
| AFUA_7G05810 | Putative ribonucleoprotein, nucleic acid binding (Mrd1p) | 1 | 1 | - | - |
| AFUA_2G04940 | mRNA binding, role in telomere maintenance (Pbp2p) | - | - | 1 | 1 |
| AFUA_6G04330 | RNA helicase, maturation of SSU-rRNA (Ecm16p) | - | - | 1 | 1 |
| **Ribosomal proteins** | |  |  |  |  |
| AFUA_1G05990 | 60S ribosomal protein (Rpl16Ap) | 2 | 1 | - | - |
| AFUA_2G04130 | 40S ribosomal protein (Rps11A) | 2 | 1 | - | - |
| AFUA_2G01830 | Protein with putative ribosomal activity | 1 | 1 | - | - |
| **Signal-transduction** | |  |  |  |  |
| AFUA_1G11730 (ArfA) | GTPase activity, role in ER/Golgi transport | 1 | 1 | - | - |
| AFUA_2G07600 | GTP binding, signal recognition activity | 1 | 1 | - | - |
| AFUA_6G07980 (NimX) | Cyclin-dependent serine/threonine kinase | 1 | 1 | - | - |
| **AFUA_6G06750** | **14-3-3 family protein; predicted gene pair with ArtA** | **2** | **1** | **-** | **2** |
| AFUA_2G03290 (ArtA) | 14-3-3 family protein; predicted gene pair with AFUA_6G06750 | - | - | 1 | 2 |
| AFUA_3G09550 (CmkB) | Calcium/calmodulin dependent protein kinase | - | - | - | 2 |
| AFUA_5G04130 (PhoA) | Putative cyclin-dependent protein kinase | - | - | 1 | 2 |
| **Metabolic enzymes** | |  |  |  |  |
| AFUA_1G12800 | Putative NADPH isocitrate dehydrogenase (Idh2p) | 1 | 1 | - | - |
| AFUA_2G04520 | Protein with metal ion binding domains, oxidoreductase activity (Adh4p) | 1 | 1 | - | - |
| AFUA_2G10920 (EchA) | Putative enoyl-CoA hydratase/isomerase family protein, role in beta oxidation of fatty acids | 1 | 1 | - | - |
| AFUA_3G08660 (IdpA) | Putative isocitrate dehydrogenase | 1 | 1 | - | - |
| AFUA_6G10660 (AclA) | Putative ATP citrate lyase subunit | 2 | - | - | - |
| **AFUA_3G11070 (PdaC)** | **Putative pyruvate decarboxylase** | **1** | **1** | **1** | **1** |
| AFUA_1G06960 | Pyruvate dehydrogenase complex subunit alpha (Pda1p) | - | - | 1 | 1 |
| AFUA_1G15640 | Domains with predicted carbon-sulfur lyase activity | - | - | 1 | 1 |
| **Fungal morphology** | |  |  |  |  |
| AFUA_4G08770 | Protein with putative microtubule binding activity | 2 | 1 | - | - |
| **AFUA_5G03080 (AspC)** | **Septin, role in cell polarity and hyphal growth** | **2** | **2** | **1** | **1** |
| AFUA_1G08850 (AspD) | Septin, localizes to long tubular structures within hyphae and to newly formed septa | - | - | 1 | 2 |
| **Unknown function** | |  |  |  |  |
| AFUA_1G09610 | Conserved hypothetical protein | 2 | 1 | - | - |
| AFUA_3G13930 | Conserved hypothetical protein | 2 | 1 | - | - |
| AFUA_2G10860 | Domains with predicted zinc ion binding activity | - | - | 1 | 1 |
