# Supplementary material for: SCF Ubiquitin Ligase F-box Protein Fbx15 Controls Nuclear Co-repressor Localization, Stress Response and Virulence of the Human Pathogen Aspergillus fumigatus
Source: PLoS Pathog. 2016 Sep 20;12(9):e1005899. doi: 10.1371/journal.ppat.1005899 (PMC5029927; doi:10.1371/journal.ppat.1005899)
Supplement: S4 Table — (DOCX) [file ppat.1005899.s012.docx]

**Table S4. *Aspergillus fumigatus* strains used in this study.**

| **Strain** | **Genotype** | **Reference** |
| --- | --- | --- |
|  |  |  |
| D141 | Clinical isolate, wild type | [1] |
| AfS35 (FGSC# 1159) | *akuA::loxP* | [2] |
| AfGB5 | ∆*akuA*, ∆*fbx15::ptrA* | This study |
| AfGB8 | ∆*akuA*, ∆*fbx23::ptrA* | This study |
| AfGB10 | ∆*akuA*, ∆*grrA::ptrA* | This study |
| AfGB15 | ∆*akuA*, ∆*fbx15::ptrA*, *fbx15*, *hph* | This study |
| AfGB18 | ∆*akuA*, ∆*fbx23::ptrA*, *fbx23*, *hph* | This study |
| AfGB20 | ∆*akuA*, ∆*grrA::ptrA*, *grrA*, *hph* | This study |
| AfGB32 | ∆*akuA*, ∆*fbx15::ptrA::^p^gpdA::fbx15::gfp* | This study |
| AfGB33 | ∆*akuA*, ∆*fbx15::ptrA::^p^gpdA::fbx15::tap* | This study |
| AfGB34 | ∆*akuA*, ∆*sconB::ptrA::^p^gpdA::sconB::gfp* | This study |
| AfGB35 | ∆*akuA*, ∆*sconB::ptrA::^p^gpdA::sconB::tap* | This study |
| AfGB40 | ∆*akuA*, ∆*fbx15::ptrA::^p^gpdA::fbx15*[P12S]*::gfp* | This study |
| AfGB41 | ∆*akuA*, ∆*fbx15::ptrA::^p^gpdA::fbx15*[P12S]*::tap* | This study |
| AfGB42 | ∆*akuA*, ∆*sconB::ptrA::^p^gpdA::sconB*[P200S]*::gfp* | This study |
| AfGB43 | ∆*akuA*, ∆*sconB::ptrA::^p^gpdA::sconB*[P200S]*::tap* | This study |
| AfGB57 | ∆*akuA*, *ptrA*, *^p^gpdA::fbx15::^t^his2A* | This study |
| AfGB64 | ∆*akuA*, ∆*ssnF::hph::ssnF::gfp* | This study |
| AfGB65 | ∆*akuA*, ∆*fbx15::ptrA*, ∆*ssnF::hph::ssnF::gfp* | This study |
| AfGB66 | ∆*akuA*, *ptrA*, *^p^gpdA::fbx15::^t^his2A*, ∆*ssnF::hph::ssnF::gfp* | This study |
| AfGB67 | ∆*akuA*, ∆*nic96::hph::nic96::gfp* | This study |
| AfGB68 | ∆*akuA*, ∆*fbx15::ptrA*, ∆*nic96::hph::nic96::gfp* | This study |
| AfGB69 | ∆*akuA*, *ptrA*, *^p^gpdA::fbx15::^t^his2A*, ∆*nic96::hph::nic96::gfp* | This study |
| AfGB70 | ∆*akuA*, *ptrA*, *^p^gpdA::gfp::^t^his2A* | This study |
| AfGB98 | *∆akuA, fbx15::rfp::^t^trpC::ptrA, ∆ssnF::ssnF::gfp::hph* | This study |
| AfGB101 | *∆akuA, fbx15*[S468A; S469A]*::rfp::^t^trpC::ptrA, ∆ssnF::ssnF::gfp::hph* | This study |
| AfGB102 | *∆akuA, fbx15*[S469D]*::rfp::^t^trpC::ptrA, ∆ssnF::ssnF::gfp::hph* | This study |
| AfGB125 | *∆akuA, fbx15*[∆F-box]*::rfp::^t^trpC::ptrA* | This study |
| AfGB126 | *∆akuA, fbx15*[∆F-box; S468A; S469A]*::rfp::^t^trpC::ptrA* | This study |
| AfGB127 | *∆akuA, fbx15*[∆F-box; S469D]*::rfp::^t^trpC::ptrA* | This study |
| AfGB128 | *∆akuA*, ∆*fbx15* | This study |
| **Bimolecular fluorescence complementation strains** | | |
| Af293.1  (FGSC# 1137) | *pyrG1* | Fungal Genetics Stock Center, Kansas City, Missouri USA [3] |
| AfGB44 | *pyrG1*, *pyrG*, *^p^niiA::cyfp::skpA::^t^niiA*, *^p^niaA::nyfp::fbx15::^t^niaA*, (pME4056 in Af293.1) | This study |
| AfGB45 | *pyrG1*, *pyrG*, *^p^niiA::cyfp::skpA::^t^niiA*, *^p^niaA::nyfp::sconB::^t^niaA*, (pME4058 in Af293.1) | This study |
| AfGB93 | *pyrG1*, *pyrG*, *^p^niiA::cyfp::fbx15::^t^niiA*, *^p^niaA::nyfp::ssnF::^t^niaA* (pME4302 in Af293.1) | This study |
| AfGB120 | *pyrG1*, *pyrG*, *^p^niiA::cyfp::skpA::^t^niiA*, *^p^niaA::nyfp::fbx15*[S468A; S469A]*::^t^niaA* (pME4434 in Af293.1) | This study |
| AfGB121 | *pyrG1*, *pyrG*, *^p^niiA::cyfp::ssnF::^t^niiA*, *^p^niaA::nyfp::fbx15*[S468A; S469A]*::^t^niaA* (pME4433 in Af293.1) | This study |
| AfGB123 | *pyrG1*, *pyrG*, *^p^niiA::cyfp::fbx15::^t^niiA*, *^p^niaA::nyfp::glcA::^t^niaA*, (pME4468 in Af293.1) | This study |
| AfGB124 | *pyrG1*, *pyrG*, *^p^niiA::cyfp::fbx15::^t^niiA*, *^p^niaA::nyfp::nimX::^t^niaA*, (pME4469 in Af293.1) | This study |

### **References**

1. Reichard U, Büttner S, Eiffert H, Staib F, Rüchel R. Purification and characterisation of an extracellular serine proteinase from *Aspergillus fumigatus* and its detection in tissue. J Med Microbiol. 1990;33: 243–251.

2. Krappmann S, Sasse C, Braus GH. Gene Targeting in *Aspergillus fumigatus* by Homologous Recombination Is Facilitated in a Nonhomologous End- Joining-Deficient Genetic Background. Eukaryot Cell. 2006;5: 212–215. doi:10.1128/EC.5.1.212-215.2006

3. McCluskey K, Wiest A, Plamann M. The Fungal Genetics Stock Center: a repository for 50 years of fungal genetics research. J Biosci. Springer; 2010;35: 119–126. doi:10.1007/s12038-010-0014-6
