# Supplementary material for: SCF Ubiquitin Ligase F-box Protein Fbx15 Controls Nuclear Co-repressor Localization, Stress Response and Virulence of the Human Pathogen Aspergillus fumigatus
Source: PLoS Pathog. 2016 Sep 20;12(9):e1005899. doi: 10.1371/journal.ppat.1005899 (PMC5029927; doi:10.1371/journal.ppat.1005899)
Supplement: S5 Table — (DOCX) [file ppat.1005899.s013.docx]

**Table S5 Oligonucleotides used during this study.**

| **Name** | **size** | **Sequence in 5’ – 3’ direction** |
| --- | --- | --- |
| OZG-(ptrA 5) | 28-mer | AATTGATTACGGGATCCCATTGGTAACG |
| OZG-(ptrA 3) | 29-mer | TCTTGCATCTTTGTTTGTATTATACTGTC |
| OZG73 | 21-mer | ATGGTGAGCAAGGGCGAGGAG |
| OZG387 | 25-mer | CGTGGCGATGGAGCGCATGATATAG |
| OZG75 | 21-mer | ATGGCCGACAAGCAGAAGAAC |
| OZG388 | 27-mer | GTGGTTCATGACCTTCTGTTTCAGGTC |
| OZG207 | 24-mer | GGTGGTAGCGGTGGTATGGTGAGC |
| OZG209 | 43-mer | GGTGGTAGCGGTGGTAAGAGAAGATGGAAAAAGAATTTCATAG |
| HO1 | 21-mer | CTCTTCGAAGGCTGGACTTGC |
| HO2 | 22-mer | GGAGATGGCGAGGAATGATACG |
| Kt330 | 20-mer | AACATCATTCCCAGCTCGAC |
| Kt331 | 20-mer | ACGTTGGAGGTAGGAACACG |
| GliZf | 20-mer | ACGACGATGAGGAATCGAAC |
| GliZr | 20-mer | TCCAGAAAAGGGAGTCGTTG |
| GliPf | 20-mer | AAACCCCTGTGAATGCAGAC |
| GliPr | 20-mer | CCCCTTGAGATGAAAGGTGA |
| GliKf | 20-mer | CTCACGGCATACAGCGACTA |
| GliKr | 20-mer | ATAATCCAACCGAGCCACTG |
| GliTf | 20-mer | ACTCCACCATCCAGTTCCAG |
| GliTr | 20-mer | TCCGAGTATCCCTCGATGTC |
| AA1 | 41-mer | CTGCAGGAATTCGATGTTTAAACAACGGAGAATTGAGGCTC |
| AA2 | 45-mer | CTATAGGCCTGAGTGATTTAAATGTCCAAACTGAGAGGAAGAGAT |
| AA3 | 35-mer | ATAATATGGCCATCTCGATCCGTCCTCTCTAGTTG |
| AA4 | 41-mer | ATCGATAAGCTTGATGTTTAAACGGCTTGTGCTATTTGCAG |
| Afbox15 del A | 24-mer | CGTGGCTCTTTAGTCGGTCATTTG |
| Afbox15 del B | 24-mer | GGATACGATGATGATGGAACC |
| Afbox15 del C | 56-mer | CATTTCGTTACCAATGGGATCCCGTAATCAATTGTCCAAACTGAGAGGAAGAGATG |
| Afbox15 del D | 56-mer | GAAAGACAGTATAATACAAACAAAGATGCAAGACGATCCGTCCTCTCTAGTTGCAG |
| Afbox15 del E | 22-mer | GTGTAATGCTGCGTGCTCTACG |
| Afbox15 del F | 23-mer | GTCCGCCTATCAAACGGCTTGTG |
| Afbox23 del A | 21-mer | CTCTTGTCAGCTCCTCGGCAG |
| Afbox23 del B | 25-mer | CACCTAAACACTCAAGTGACAAGTG |
| Afbox23 del C | 58-mer | CATTTCGTTACCAATGGGATCCCGTAATCAATTGTGAAAAATCTTCGTCCGATAACAG |
| Afbox23 del D | 56-mer | GAAAGACAGTATAATACAAACAAAGATGCAAGAATAGTTTAACGACCTTCGTTCCC |
| Afbox23 del E | 24-mer | CTCTAACCGTCCGAATGAATATAG |
| Afbox23 del F | 24-mer | GGTACAAGCTTGGTCAGACCACTC |
| Afbox25 del A | 24-mer | CCTCGATTGGCGTTCTAATTGACC |
| Afbox25 del B | 23-mer | GCATCTGCGTGTTTTCGAGGATG |
| Afbox25 del C | 55-mer | CATTTCGTTACCAATGGGATCCCGTAATCAATTAGCGGCACTCAACCACTCATCT |
| Afbox25 del D | 58-mer | GAAAGACAGTATAATACAAACAAAGATGCAAGAAAGCTCCTTTGAGTTTGCTTTTTCT |
| Afbox25 del E | 25-mer | GATAGCATCAAAGAAAGAAACCAGG |
| Afbox25 del F | 24-mer | GGTCTTAGTTGAGATGCTGTCCTG |
| AfgrrA del A | 24-mer | GCATCCCAAGTATTGGATCATCTC |
| AfgrrA del B | 22-mer | GCTGCCTTCAGTAAGGCCAATC |
| AfgrrA del C | 54-mer | CATTTCGTTACCAATGGGATCCCGTAATCAATTTCTGGATAGCGGGTGTGAAGC |
| AfgrrA del D | 56-mer | GAAAGACAGTATAATACAAACAAAGATGCAAGATACCCCTCTGCTATTCCTTCGAG |
| AfgrrA del E | 24-mer | GTTCGACTGCGTATCGTCAAAGAC |
| AfgrrA del F | 25-mer | CGGATATACACCATGATATTGGTAC |
| BJ1 | 33-mer | AAAGGTACCGGATACGATGATGATGGAACCTTG |
| BJ2 | 38-mer | TTGTTAACTCTTGCATCTTTGTTTGTATTATACTGTCT |
| BJ3 | 24-mer | GATCTTTGCCCGGTGTATGAAACC |
| BJ4 | 21-mer | GGTGATGTCTGCTCAAGCGGG |
| BJ5 | 53-mer | AACAGCTACCCCGCTTGAGCAGACATCACCATGACCGACATGAGCAAGAACCT |
| BJ6 | 55-mer | GCCCTTGCTCACCATACCACCGCTACCACCCCGAAGCCAATATCCATGATTAATG |
| BJ7 | 52-mer | ACTCACGGCATGGACGAACTGTACAAGTAGCGATCCGTCCTCTCTAGTTGCA |
| BJ8 | 60-mer | TATGAAATTCTTTTTCCATCTTCTCTTACCACCGCTACCACCCCGAAGCCAATATCCATG |
| BJ9 | 52-mer | GACGCGAATTCCGCGGGGAAGTCAACCTGACGATCCGTCCTCTCTAGTTGCA |
| BJ10 | 32-mer | AAAGCCGGCGCATCTGCGTGTTTTCGAGGATG |
| BJ11 | 52-mer | AACAGCTACCCCGCTTGAGCAGACATCACCATGGATGCCCATGAACTGTCGT |
| BJ12 | 52-mer | GCCCTTGCTCACCATACCACCGCTACCACCGGTCTGGAAACTGTACATGCGC |
| BJ13 | 56-mer | ACTCACGGCATGGACGAACTGTACAAGTAGAAGCTCCTTTGAGTTTGCTTTTTCTT |
| BJ14 | 34-mer | TTTGCCGGCGATAGCATCAAAGAAAGAAACCAGG |
| BJ15 | 52-mer | TTTCCATCTTCTCTTACCACCGCTACCACCGGTCTGGAAACTGTACATGCGC |
| BJ16 | 56-mer | GACGCGAATTCCGCGGGGAAGTCAACCTGAAAGCTCCTTTGAGTTTGCTTTTTCTT |
| BJ17 | 25-mer | GCTGATGGAGTCCAGGTTCTTGCTC |
| BJ18 | 55-mer | GACATGAGCAAGAACCTGGACTCCATCAGCTATGATGTGTTCTACCAGATCGCTT |
| BJ19 | 23-mer | GCTGAGAGCCGTGAGGAAGTCGA |
| BJ20 | 60-mer | ATTCGTATCGACTTCCTCACGGCTCTCAGCCCAGAAATCTCTTTCAAAATTCTATGTTAT |
| BJ21 | 23-mer | ATGGATGCCCATGAACTGTCGTT |
| BJ24 | 23-mer | TCAGGTCTGGAAACTGTACATGC |
| BJ37 | 23-mer | CTACTTGTACAGTTCGTCCATGC |
| BJ38 | 20-mer | TCAGGTTGACTTCCCCGCGG |
| BJ63 | 22-mer | ATGACCGACATGAGCAAGAACC |
| BJ64 | 22-mer | GTCACCGAAGCCAATATCCATG |
| BJ65 | 52-mer | AACGACCTGAAACAGAAGGTCATGAACCACATGACCGACATGAGCAAGAACC |
| BJ66 | 52-mer | AACGTCTATATCATGCGCTCCATCGCCACGATGACCGACATGAGCAAGAACC |
| BJ67 | 53-mer | AACGACCTGAAACAGAAGGTCATGAACCACATGGATGCCCATGAACTGTCGTT |
| BJ68 | 53-mer | AACGTCTATATCATGCGCTCCATCGCCACGATGGATGCCCATGAACTGTCGTT |
| BJ69 | 55-mer | AACGACCTGAAACAGAAGGTCATGAACCACATGACTACTGTTACTCTCACAAGCT |
| BJ70 | 55-mer | AACGTCTATATCATGCGCTCCATCGCCACGATGACTACTGTTACTCTCACAAGCT |
| BJ71 | 21-mer | TTACTCCTCGGCCCACTCGTT |
| BJ162 | 22-mer | CTACAACGACCATCAAAGTCGT |
| BJ163 | 20-mer | CTATTCCTTTGCCCTCGGAC |
| BJ164 | 37-mer | CACATCTCCACTCGACTACAACGACCATCAAAGTCGT |
| BJ165 | 35-mer | ATCGATAAGCTTGATCTATTCCTTTGCCCTCGGAC |
| BJ166 | 18-mer | AGTAGATGCCGACCGCGG |
| BJ167 | 20-mer | TCGAGTGGAGATGTGGAGTG |
| BJ168 | 34-mer | CTGCAGGAATTCGATGGTGGTAGCGGTGGTATGG |
| BJ169 | 37-mer | CGGTCGGCATCTACTCTACTTGTACAGTTCGTCCATG |
| BJ174 | 45-mer | CTGCAGGAATTCGATGTTTAAACATACTAGCTACTAAGCCTCCTA |
| BJ175 | 33-mer | ACCACCGCTACCACCGGCCGACTCCTGTTTCGA |
| BJ176 | 35-mer | AGGGCAAAGGAATAGGCACATGATCCTCTCTCGTC |
| BJ177 | 40-mer | ATCGATAAGCTTGATGTTTAAACCGCTCAGGTGCTCAGCC |
| BJ234 | 29-mer | ACTCGAGATGACCGACATGAGCAAGAACC |
| BJ235 | 29-mer | TCTCGAGGTCACCGAAGCCAATATCCATG |
| BJ244 | 25-mer | AGAATTCATGGCTCACACTCAGCCG |
| BJ245 | 26-mer | TGAATTCTCAGGCCGACTCCTGTTTC |
| BJ256 | 21-mer | GGACGGGAAGCGGATAGAACT |
| BJ257 | 22-mer | CAGAGGGAGCCTTGGTCATACA |
| BJ266 | 49-mer | CTGCAGGAATTCGATGTTTAAACTGTATTAGAGGACATCATGCTAATTT |
| BJ267 | 34-mer | ACCACCGCTACCACCGTAAGCCCCGATATCCGCT |
| BJ268 | 38-mer | AGGGCAAAGGAATAGTGCTTATCATCTGGAGTATCTGG |
| BJ269 | 47-mer | ATCGATAAGCTTGATGTTTAAACACCTCCAGCTTCAATCAATTTTTC |
| BJ290 | 46-mer | CTGCAGGAATTCGATGTTTAAACATACTAGCTACTAAGCCTCCTAT |
| BJ291 | 32-mer | ATCCCGTAATCAATTTCTGCTGGCGGGAGGAC |
| BJ292 | 35-mer | AACAAAGATGCAAGAGCACATGATCCTCTCTCGTC |
| BJ293 | 40-mer | ATCGATAAGCTTGATGTTTAAACCGCTCAGGTGCTCAGCC |
| BJ295 | 48-mer | AACGTCTATATCATGCGCTCCATCGCCACGGCTCACACTCAGCCGTCG |
| BJ304 | 19-mer | CTGGGTGGGGGTCGTTGAC |
| BJ306 | 37-mer | ACGACCCCCACCCAGCAGCACCAGGAGGCAGCCACAT |
| BJ309 | 39-mer | CTGCAGGAATTCGATGATATCAGTAGATGCCGACCGCGG |
| BJ312 | 46-mer | ATCGATAAGCTTGATGTTTAAACACCTAGTTCAGAGATATGTCCTG |
| BJ313 | 45-mer | CTGCAGGAATTCGATGATGTTTAAACTGCATGCTGTCCAGTTGGC |
| BJ316 | 35-mer | ATCCCGTAATCAATTTCGAGTGGAGATGTGGAGTG |
| BJ317 | 35-mer | AACAAAGATGCAAGACGATCCGTCCTCTCTAGTTG |
| BJ318 | 24-mer | CCGAAGCCAATATCCATGATTAAT |
| BJ323 | 43-mer | CTGCAGGAATTCGATATTTAAATTGGGAAGGTTAGATAGGGGC |
| BJ324 | 35-mer | TGATGGTCGTTGTAGGACGTGCTGATTCTCGAGAC |
| BJ325 | 39-mer | CACATCTCCACTCGAGAAATCATACGAGCTTTTCCATGT |
| BJ326 | 42-mer | ATCGATAAGCTTGATATTTAAATCCCGAAGTCAGCGGGATTG |
| BJ327 | 42-mer | CTGCAGGAATTCGATATTTAAATAGCTGTGCGCAGCAGCTTG |
| BJ328 | 32-mer | TGATGGTCGTTGTAGGGTGGCGGTCTCCTTGC |
| BJ329 | 39-mer | CACATCTCCACTCGACTGAAAACTTGTCACGACTTTTAC |
| BJ330 | 42-mer | ATCGATAAGCTTGATATTTAAATACCGGGGCACTTGGTACTG |
| BJ336 | 22-mer | GTCTGATGGGTGGGGGTCGTTG |
| BJ337 | 33-mer | CCCCACCCATCAGACCCAGGAGGCAGCCACATC |
| BJ338 | 21-mer | GTCTGGGTGGGGGTCGTTGAC |
| BJ340 | 36-mer | GACCCCCACCCAGACGACCCAGGAGGCAGCCACATC |
| BJ344 | 34-mer | CATACTCTCACATTTATGGTGAGCAAGGGCGAGG |
| BJ371 | 21-mer | GTCCAAACTGAGAGGAAGAGA |
| BJ372 | 38-mer | CCTCTCAGTTTGGACATGAACCACCTGCTTCATTCCAA |
| BJ375 | 22-mer | AGCTTTGAGGATGTCTGGTCTC |
| BJ376 | 22-mer | GTTCTTCACCACGGGAGATTTA |
| BJ377 | 36-mer | CGCTCCATCGCCACGATGGCTGATCAAGAAGTGGAT |
| BJ378 | 36-mer | TGCGAACCCGTATTTTTACTTCTTCTTTGGTTTGCG |
| BJ379 | 39-mer | CGCTCCATCGCCACGATGGAGAATTACCAGAAAATTGAG |
| BJ380 | 37-mer | TGCGAACCCGTATTTTTAGTGGTATCCATTTCTCCGA |
