# Supplementary material for: SCF Ubiquitin Ligase F-box Protein Fbx15 Controls Nuclear Co-repressor Localization, Stress Response and Virulence of the Human Pathogen Aspergillus fumigatus
Source: PLoS Pathog. 2016 Sep 20;12(9):e1005899. doi: 10.1371/journal.ppat.1005899 (PMC5029927; doi:10.1371/journal.ppat.1005899)
Supplement: S6 Table — (DOCX) [file ppat.1005899.s014.docx]

**Table S6. Plasmids constructed and used during this study.**

| **Plasmid** | **Description** | **Reference** |
| --- | --- | --- |
| pAN7-1 | hygromycin B resistance (hph) vector | [1] |
| pJET1.2 | blunt Cloning Vector: CloneJET^TM^, *amp^R^* | Fermentas GmbH |
| pBluescript II KS+ | Cloning Vector | Fermentas GmbH |
| pME3024 | pyrithiamine resistance (*ptrA*) vector | [2] |
| pME3160 | Expression module *^t^niiA-^p^niiA* / *^p^niaD-^t^niaD*, *pyrG*, *amp^R^* | [3] |
| pSK379 | pyrithiamine resistance (*ptrA*) vector including *gpdA* promoter for high expression | [4] |
| pSK485 | self-excising β-rec/*six* *ptrA* marker cassette containing vector | [5] |
| pME3701 | *fbx15* with 1,5 kb flanking sites in pAN7-1 | This study |
| pME3704 | *fbx23* with 1,5 kb flanking sites in pAN7-1 | This study |
| pME3706 | *grrA* with 1,5 kb flanking sites in pAN7-1 | This study |
| pME4042 | *fbx15 5'UTR::ptrA::3’UTR* in pJET1.2 | This study |
| pME4043 | *sconB 5'UTR::ptrA::3’UTR* in pJET1.2 | This study |
| pME4044 | *fbx15 5'UTR::ptrA::^p^gpdA::fbx15::gfp::3'UTR* in pJET1.2 | This study |
| pME4045 | *fbx15 5'UTR::ptrA::^p^gpdA::fbx15::tap::3'UTR* in pJET1.2 | This study |
| pME4046 | *sconB 5'UTR::ptrA::^p^gpdA::sconB::gfp::3'UTR* in pJET1.2 | This study |
| pME4047 | *sconB 5'UTR::ptrA::^p^gpdA::sconB::tap::3'UTR* in pJET1.2 | This study |
| pME4048 | *fbx15 5'UTR::ptrA::^p^gpdA::fbx15*[P12S]*::gfp::3'UTR* in pJET1.2 | This study |
| pME4049 | *fbx15 5'UTR::ptrA::^p^gpdA::fbx15*[P12S]*::tap::3'UTR* in pJET1.2 | This study |
| pME4050 | *sconB 5'UTR::ptrA::^p^gpdA::sconB*[P200S]*::gfp::3'UTR* in pJET1.2 | This study |
| pME4051 | *sconB 5'UTR::ptrA::^p^gpdA::sconB*[P200S]*::tap::3'UTR* in pJET1.2 | This study |
| pME4052 | *cYFP::skpA* in *Pme*I-site of pME3160 | This study |
| pME4056 | *nYFP::fbx15* in *Swa*I-site of pME4052 | This study |
| pME4058 | *nYFP::sconB* in *Swa*I-site of pME4052 | This study |
| pME4284 | *^p^gpdA::fbx15* in *Stu*I-site of pAN7-1 | This study |
| pME4285 | *gfp::^t^trpC::^p^gpdA::hph* in pBluescript II KS+ | This study |
| pME4286 | *ssnF 5'UTR::ssnF::gfp:: ^t^trpC:: ^p^gpdA::hph::3'UTR* in pBluescript II KS+ | This study |
| pME4289 | *fbx15* in *Mss*I-site of pSK379 | This study |
| pME4291 | *nic96 5'UTR::nic96::gfp:: ^t^trpC:: ^p^gpdA::hph::3'UTR* in pBluescript II KS+ | This study |
| pME4292 | *gfp* in *Mss*I-site of pSK379 | This study |
| pME4294 | *ssnF 5'UTR::ptrA::3'UTR* in pBluescript II KS+ | This study |
| pME4298 | *fbx15* cDNA in pBluescript II KS+ | This study |
| pME4300 | *ssnF* cDNA in pBluescript II KS+ | This study |
| pME4301 | *cYFP::fbx15* (cDNA) in *Mss*I-site of pME3160 | This study |
| pME4302 | *nYFP::ssnF* (cDNA) in *Smi*I-site of pME4301 | This study |
| pME4341 | (*EcoR*V-site)*::^t^trpC::ptrA::fbx15* 3’UTR in *EcoR*V-site of pBluescript II KS+ | This study |
| pME4342 | *fbx15* 5’UTR*::fbx15::rfp* in *EcoR*V-site of pME4341 | This study |
| pME4345 | *fbx15* 5’UTR*::fbx15* [S468A; S469A]*::rfp* in *EcoR*V-site of pME4341 | This study |
| pME4346 | *glcA 5'UTR::^p^gpdA::hph::^t^trpC::3'UTR* in *EcoR*V-site of pBluescript II KS+ | This study |
| pME4347 | *nimX 5'UTR::^p^gpdA::hph::^t^trpC::3'UTR* in *EcoR*V-site of pBluescript II KS+ | This study |
| pME4348 | *fbx15* 5’UTR*::fbx15* [S469D]*::rfp* in *EcoR*V-site of pME4341 | This study |
| pME4432 | *nYFP::fbx15* [S468A; S469A] in *Swa*I-site of pME3160 | This study |
| pME4433 | *cYFP::ssnF* in *Pme*I-site of pME4432 | This study |
| pME4434 | *nYFP::fbx15* [S468A; S469A] in *Swa*I-site of pME4052 | This study |
| pME4468 | *nYFP::glcA* (cDNA) in *Smi*I-site of pME4301 | This study |
| pME4469 | *nYFP::nimX* (cDNA) in *Smi*I-site of pME4301 | This study |
| pME4475 | *fbx15* 5’UTR*::fbx15* [-F-box]*::rfp* in *EcoR*V-site of pME4341 | This study |
| pME4476 | *fbx15* 5’UTR*::fbx15* [-F-box; S468A; S469A]*::rfp* in *EcoR*V-site of pME4341 | This study |
| pME4477 | *fbx15* 5’UTR*::fbx15* [-F-box; S469D]*::rfp* in *EcoR*V-site of pME4341 | This study |
| pME4538 | *fbx15 5'UTR::six-*site*::^p^xylP::*β*-rec::^t^trpC::ptrA::six-*site*::3'UTR* in *EcoR*V-site of pBluescript II KS+ | This study |

### **References**

1. Punt PJ, Oliver RP, Dingemanse MA, Pouwels PH, van den Hondel CA. Transformation of *Aspergillus* based on the hygromycin B resistance marker from *Escherichia coli*. Gene. 1987;56: 117–124.

2. Krappmann S, Jung N, Medic B, Busch S, Prade RA, Braus GH. The *Aspergillus nidulans* F-box protein GrrA links SCF activity to meiosis. Mol Microbiol. 2006;61: 76–88. doi:10.1111/j.1365-2958.2006.05215.x

3. Bayram Ö, Krappmann S, Ni M, Bok JW, Helmstaedt K, Valerius O, et al. VelB/VeA/LaeA Complex Coordinates Light Signal with Fungal Development and Secondary Metabolism. Science. 2008;320: 1504–1506. doi:10.1126/science.1155888

4. Wagener J, Echtenacher B, Rohde M, Kotz A, Krappmann S, Heesemann J, et al. The Putative α-1,2-Mannosyltransferase AfMnt1 of the Opportunistic Fungal Pathogen *Aspergillus fumigatus* Is Required for Cell Wall Stability and Full Virulence. Eukaryot Cell. 2008;7: 1661–1673. doi:10.1128/EC.00221-08

5. Hartmann T, Dümig M, Jaber BM, Szewczyk E, Olbermann P, Morschhäuser J, et al. Validation of a Self-Excising Marker in the Human Pathogen *Aspergillus fumigatus* by Employing the β-Rec/*six* Site-Specific Recombination System. Appl Environ Microbiol. 2010;76: 6313–6317. doi:10.1128/AEM.00882-10
