# Supplementary material for: SCF Ubiquitin Ligase F-box Protein Fbx15 Controls Nuclear Co-repressor Localization, Stress Response and Virulence of the Human Pathogen Aspergillus fumigatus
Source: PLoS Pathog. 2016 Sep 20;12(9):e1005899. doi: 10.1371/journal.ppat.1005899 (PMC5029927; doi:10.1371/journal.ppat.1005899)
Supplement: S1 Text — (DOCX) [file ppat.1005899.s015.docx]

## Supplemental Text S1

### Bioinformatics

F-box domains of *A. fumigatus* Fbx15, SconB and GrrA were identified by amino‑acid sequence analysis for Pfam-domain matches (<http://pfam.sanger.ac.uk>).

Homologues F-box proteins for *A. fumigatus* Fbx15, SconB and GrrA in other species were identified by NCBI-BLAST with blastp-algorithm (<http://blast.ncbi.nlm.nih.gov/Blast.cgi>) and are shown in Table S1.

Additional Fbx15 specific motifs were identified by comparing protein sequences of Fbx15 homologs with MEME: <http://meme.nbcr.net/meme/> [1] and are shown in Table S2.

Nuclear localization sequences (NLS) were determined using the cNLS Mapper program: <http://nls-mapper.iab.keio.ac.jp/cgi-bin/NLS_Mapper_form.cgi> [2].

Putative phosphorylation sites of Fbx15 primary amino acid sequence were analyzed with NetPhos 2.0 (<http://www.cbs.dtu.dk/services/NetPhos>) [3].

Analysis of the MS2‑spectra for the identification of specific phopho-sites inside identified phosphopeptides was done with the phosphoRS software [4].

### Growth conditions

All *A. fumigatus* strains used in this study were grown at 37°C in aspergillus minimal medium (MM: 1 % D-glucose, 1x AspA (70 mM NaNO_3_, 7 mM KCl, 11.2 mM KH_2_PO_4_, pH 5.5), 2 mM MgSO_4_, 1x trace elements (76 µM ZnSO_4_, 178 µM H_3_BO_3_, 25 µM MnCl_2_, 18 µM FeSO_4_, 7.1 µM CoCl_2_, 6.4 µM CuSO_4_, 6.2 µM Na_2_MoO_4_, 174 µM EDTA) if not otherwise stated. For selective media either pyrithiamine (100 ng/ml) or hygromycin G (150 µg/ml) were added. Stress-inducing agents, which were applied during growth tests for ∆*fbx* mutant strains (refers to Figure S1C) included alternate carbon sources Sorbitol and Lactose (instead of Glucose), 2 µg/ml Benomyl for microtubule-stress, 5 mM 5-Methyl-DL-tryptophan (5-MT) and 1 mM 3‑Amino‑1.2.4‑triazol (3‑AT) for amino-acid starvation, 1 M KCl for inducing osmotic stress, 0.01 % methyl methanesulfonate (MMS) and 50 µM camptothecin (CPT) providing mutagenic stress and 3 mM H_2_O_2_ for oxidative stress. For the detection of gliotoxin produced by the mutant strains as well as for determination of *gli-*gene expression cultures were grown in Czapek-Dox medium (3 % sucrose, 1x AspA (70 mM NaNO_3_, 7 mM KCl, 11.2 mM KH_2_PO_4_, pH 5.5), 2 mM MgSO_4_, 36 µM FeSO_4_) at 28°C for five days. No significant differences in mycelial mass between wild type and mutant strains could be observed.

### Recombinant DNA-techniques and analysis of nucleic acids

Generation of linear and circular DNA molecules were based on standard recombinant DNA technology protocols as described previously [5]. Oligonucleotides and plasmids used in this study are listed in tables S5 and S6 respectively. For polymerase chain reaction (PCR) either Taq- (Fermentas) or Phusion-polymerase (Finnzymes) were used. Fusion of PCR-products was achieved by using fusion PCR protocol which is described in detail [6] or the GENEART® Seamless Cloning and Assembly Kit (Invitrogen). Genomic DNA from *A. fumigatus* strains was extracted as described [5]. Southern hybridization was carried out due to standard protocols reported previously [5]. DNA-Probes were amplified from D141 genomic DNA with primers listed in Table S5 and either radioactive labeled with α-^32^P-dATP (Hartmann Analytic GmbH) using the „HexaLabel™ DNA Labeling-Kit“ (MBI Fermentas) or chemiluminescent labeled with “Amersham Gene Images AlkPhos Direct Labelling and Detection System” (GE Healthcare).

### Construction of ∆*fbx*- and complemented strains

The construction of ∆*fbx* strains was achieved by the replacement of the respective *fbx*-gene with a pyrithiamine resistance marker cassette (*ptrA*) via homologues recombination. *A. fumigatus* transformation was done by polyethylene glycol mediated protoplast fusion as described [7].

For the *fbx15*, *fbx23*, *sconB* and *grrA* knockout cassettes 1.5 kb 5’ and 3’ UTR flanking regions of the loci Afu3g14150 (*fbx15*), Afu4g11440 (*fbx23*), Afu2g14110 (*sconB*) and Afu1g05970n (*grrA*) were amplified from *A. fumigatus* D141 genomic DNA with: (*fbx15*) Afbox15 del A/C and Afbox15 del D/F; (*fbx23*) Afbox23 del A/C and Afbox23 del D/F; (*sconB*) Afbox25 del A/C and Afbox25 del D/F; (*grrA*) AfgrrA del A/C and AfgrrA del D/F respectively. The flanking regions were fused to *ptrA*, which was amplified from pME3024 with OZG-(ptrA 5)/OZG-(ptrA 3). Fusion of the flanking sites with *ptrA* was done using fusion PCR with: (*fbx15*) Afbox15 del B/D; (*fbx23*) Afbox23 del B/D; (*sconB*) Afbox25 del B/D; (*grrA*) AfgrrA del B/D as described in detail [6]. The resulting knockout cassettes of 4.8 kb were transformed into AfS35, generating AfGB5, AfGB8 and AfGB10 respectively. For the generation of the complementation plasmids the genomic loci of Afu3g14150 (*fbx15*), Afu4g11440 (*fbx23*) and Afu1g05970n (*grrA*) including 1.5 kb 5’ and 3’ UTR flanking sites were amplified with: (*fbx15*) Afbox15 del A/F, (*fbx23*) Afbox23 del A/F and (*grrA*) AfgrrA del A/F respectively and subcloned into *Stu*I-site of pAN7-1 carrying a hygromycin resistance marker. The resulting complementation plasmids pME3701 (*fbx15*), pME3704 (*fbx23*) and pME3706 (*grrA*) were transformed into corresponding ∆*fbx*-strains generating reconstructed *fbx*-strains AfGB15 (*fbx15^+^*), AfGB18 (*fbx23^+^*) and AfGB20 (*grrA^+^*). Deletion and complementation of *fbx*-genes were verified by Southern hybridization.

### Construction of overexpressed GFP- and TAP-tagged Fbx15 and SconB strains

To achive *sgfp*- or *ctap*-tagged overexpression constructs of *fbx15* and *sconB* which were suitable for the substitution of the original loci Afu3g14150 (*fbx15*) and Afu2g14110 (*sconB*) 5’ UTR flanking sites for *fbx15* and *sconB* were amplified with BJ1/Afbox15 del C and BJ10/Afbox25 del C respectively and fused to *ptrA* amplified with OZG-(ptrA 5)/BJ2 from pME3024. The resulting 5’ UTR::*ptrA* modules containing a *Kpn*I-site (before *fbx15* 5’ UTR) or a *Nae*I-site (before *sconB* 5’ UTR) and a *Hpa*I-site (in 3’ direction after *ptrA*) *were* subcloned to pJET1.2 resulting in pME4042 and pME4043. Next, different modules were amplified: *^p^gpdA* promoter was amplified from pME3024 with BJ3/BJ4; *fbx15* was amplified from D141 genomic DNA with BJ5/BJ6 (for *sgfp* tagged version) or BJ5/BJ8 (for *ctap*-tagged version); *sconB* was amplified from D141 genomic DNA with BJ11/BJ12 (for *sgfp*-tagged version) or BJ11/BJ15 (for *ctap*-tagged version); *sgfp* was amplified from pME3167 with OZG207/BJ37; *ctap* was amplified from pME3154 with OZG209/BJ38; *fbx15* 3’ UTR flanking region was amplified from D141 genomic DNA with BJ7/Afbox15 del F (for *sgfp*-tagged version) or BJ9/Afbox15 del F (for *ctap*-tagged version); *sconB* 3’ UTR flanking region was amplified from D141 genomic DNA with BJ13/Afbox25 del F (for *sgfp*-tagged version) or BJ16/Afbox25 del F (for *ctap*-tagged version). For the *sgfp*-tagged version of *fbx15* the modules *^p^gpdA*, BJ5/BJ6, *sgfp* and BJ7/Afbox15 del F were fused by fusion PCR with BJ41/Afbox15 del E. For the *ctap*-tagged version of *fbx15* the modules *^p^gpdA*, BJ5/BJ8, *ctap* and BJ9/Afbox15 del F were fused by fusion PCR with BJ41/Afbox15 del E. The fusion PCR products were cloned into the *Hpa*I-site of pME4042 resulting in pME4044 and pME4045. For the *sgfp*-tagged version of *sconB* the modules *^p^gpdA*, BJ11/BJ12, *sgfp* and BJ13/Afbox25 del F were fused by fusion PCR with BJ41/BJ14. For the *ctap*-tagged version of *sconB* the modules *^p^gpdA*, BJ11/BJ15, *ctap* and BJ16/Afbox25 del F were fused by fusion PCR with BJ41/BJ14. The fusion PCR products were cloned into the *Hpa*I-site of pME4043 resulting in pME4046 and pME4047.

For the construction of overexpressed *fbx15::sgfp* and *fbx15::ctap* strains the corresponding constitutive constructs were excised from pBJ12 and pBJ13 by digestion with *Kpn*I and transformed into AfS35 resulting in:

AfGB32 (*∆akuA, fbx15∆::ptrA::^p^gpdA::fbx15::sgfp*)

AfGB33 (*∆akuA, fbx15∆::ptrA::^p^gpdA::fbx15::ctap*).

The construction of constitutively expressed *sconB::sgfp* and *sconB::ctap* strains was done in the same fashion. Corresponding constructs were taken from pBJ14 and pBJ15 by digestion with *Nae*I and transformed into AfS35 resulting in:

AfGB34 (*∆akuA, ∆sconB::ptrA::^p^gpdA::sconB::sgfp*) and

AfGB35 (*∆akuA, ∆sconB::ptrA::^p^gpdA::sconB::ctap*).

Replacement of the original locus was verified by Southern hybridization.

For the generation of constitutively expressed GFP- and TAP-tagged Fbx15(P12S) strains two parts from pME4044 or pME4045 respectively were amplified with BJ41/BJ17 and BJ20/Afbox15 del E, introducing a point mutation which results in an exchange of proline to serine at position 12 of the Fbx15 amino acid sequence. These PCR fragments were fused together with BJ41/Afbox15 del E and subsequently cloned into *Hpa*I-site of pME4042, resulting in pME4048 and pME4049. The generation of constitutively expressed GFP- and TAP-tagged SconB(P200S) strains was done in the same way. First corresponding parts from pME4046 or pME4047 were amplified with BJ41/BJ19 and BJ20/BJ14 before fusing them together with BJ41/BJ14, which introduces a point mutation in the *sconB* sequence resulting in an exchange of proline to serine at position 200 of the SconB aa-sequence. The fused products were then cloned into pME4043 resulting in pME4050 and pME4051. Restriction of pME4048 – pME4051 and subsequent transformation of the tagged constructs of *fbx15* and *sconB* with the introduced point mutations was done as described above, resulting in strains:

AfGB40 (*∆akuA, fbx15∆::ptrA::^p^gpdA::fbx15*(P12S)*::sgfp*)

AfGB41 (*∆akuA, fbx15∆::ptrA::^p^gpdA::*fbx15(P12S)*::ctap*)

AfGB42 (*∆akuA, ∆sconB::ptrA::^p^gpdA::sconB*(P200S)*::sgfp*)

AfGB43 (*∆akuA, ∆sconB::ptrA::^p^gpdA::sconB*(P200S)*::ctap*)

Correct integration of the fusion constructs was proved with Southern hybridization.

### Construction of *fbx15* and *gfp* overexpression strains

Overexpression of *fbx15* and *gfp* was achieved by cloning *fbx15* or *gfp* into a plasmid with constitutive *gpdA^P^* promoter, which was ectopically integrated into AfS35 (WT) strain. Therefore *fbx15* was amplified from D141 gDNA with primers BJ63/BJ64 and subsequently cloned into *Mss*I-site of pSK379, resulting in pME4289. Similar, *sgfp* was amplified from pME3167 with OZG207/BJ37 and cloned into *Mss*I-site of pSK379 to achieve pME4292. Both plasmids were transformed into AfS35 (WT) strain resulting in Aspergillus strains AfGB57 (*fbx15* overexpression) and AfGB70 (*sgfp* overexpression).

### Construction of ∆*nimX*, ∆*ssnF* and ∆*glcA* mutants

The deletion cassettes for *ssnF* (Afu2g11840), *nimX* (Afu5g04130) and *glcA* (Afu1g04950) were constructed using the “GENEART Seamless Cloning and Assembly Kit” (invitrogen). The *nimX* deletion cassette was constructed using the hygromycin B resistance marker *hph*, which was amplified from pAN7-1 with primers BJ162/BJ167. *nimX* 5’- and 3’-UTR flanking sites were amplified from D141 genomic DNA using primer pairs BJ327/BJ328 and BJ329/BJ330, which introduced two 15 bp overhangs on each fragment, complementary to either the recipient vector pBluescript II KS+ or the *hph*-cassette. All fragments were fused into the pBluescript II KS+ vector in a seamless cloning reaction. The *nimX* deletion cassette was excised from the resulting plasmid pME4347 and transformed into AfGB32.

For *ssnF* deletion cassette, first 5’- and 3’UTR flanking sites of *ssnF* were amplified from D141 gDNA with primer pairs BJ290/BJ291 and BJ292/BJ293 respectively. Subsequently they were fused to *ptrA* resistance marker and pBluescript II KS+ in a seamless cloning reaction, producing pME4294. The deletion cassette was cut from the plasmid with *Mss*I and transformed into AfS35 (WT). The final deletion of either *nimX* or *ssnF* was not possible due to its essential function for *A. fumigatus*, which was proved by Heterokaryon-recue and Southern hybridization (Figure S6).

For the *glcA* deletion cassette, 5’- and 3’UTR flanking sites of *glcA* were amplified from WT gDNA with primer pairs BJ323/BJ324 and BJ325/BJ326 respectively. These oligonucleotides introduced a *Swa*I-restriction site followed by a 15 bp complementary overhang for pBluescript II KS+ at the 5’-end of the 5’UTR and at the 3’-end of the 3’UTR fragment. They further added a 15 bp overhang complementary to the hygromycin resistance marker cassette, which was amplified from pAN7-1 with primers BJ162/BJ167. The flanking sites and the hygromycin resistance marker were fused into *EcoR*V-site of pBluescript II KS+ in a seamless cloning reaction, leading to pME4346. The *glcA* deletion cassette was excised using *Swa*I and subsequently transformed into AfGB32 (Fbx15‑GFP overexpression). Like for ∆*ssnF*, Heterokaryon-rescue and Southern hybridization showed that *glcA* is essential for *A. fumigatus* (Figure S3C/D).

### Generation of GFP-tagged fusions of SsnF and Nic96

GFP-tagged fusion of SsnF and Nic96 were constructed with “GENEART Seamless Cloning and Assembly Kit” (invitrogen). First we constructed a fusion cassette containing *sgfp* followed by a *^t^trpC* terminator sequence, which was fused to *hph* hygromycin resistance marker. For this approach we amplified *sgfp* with oligonucleotides BJ168/BJ169, introducing a 15 bp overhang complementary to pBluescript II KS+ and a 15 bp overhang complementary to the *^t^trpC* terminator sequence. The *^t^trpC* sequence was amplified with primers BJ166/BJ167 from pAN7‑1. The hygromycin resistance marker was amplified from pAN7‑1 as well with primers BJ164/BJ165, which inserted a 15 bp *^t^trpC* overhang at the 3’end and a 15 bp pBluescript II KS+ overhang at the 5’end. All fragments were fused into pBluescript II KS+ in a seamless cloning reaction, producing pME4285. The *gfp::^t^trpC::hph* cassette was subsequently amplified from pME4285 with oligonucleotides OZG207/BJ163 and used for further fusion constructs.

To construct the GFP-fusions of SsnF and Nic96, we amplified each 5’UTR region together with the respective ORF with primer pairs BJ174/BJ175 and BJ266/BJ267 for *ssnF* and *nic96* respectively. These primer pairs introduced an *Mss*I-site with a 15 bp overhang complementary to pBluescript II KS+ on the 5’end and a 15 bp *sgfp* overhang on the 3’end, while the stop codon was eliminated. Furthermore the 3’UTR flanking regions of the respective genes were amplified with primer pairs BJ176/BJ177 and BJ268/BJ269, which introduced a 15 bp overhang for *hph* hygromycin resistance marker at the 5’end and an *Mss*I-site accompanied by a 15 bp overhang of pBluescript II KS+ at the 3’end. Afterwards the single fragments including 5’UTR::ORF, *sgfp* and 3’UTR were fused into the *Mss*I-site of pBluescript II KS+ in a seamless cloning reaction, resulting in plasmids pME4286 and pME4291. The *sgfp*-fusions of *ssnF* and *nic96* were excised from their respective plasmids and transformed into AfS35 (WT) strain, ∆*fbx15* mutant (AfGB5) or *fbx15* overexpression strain (AfGB57). We obtained the strains AfGB64, AfGB65 and AfGB66 for *ssnF::sgfp* fusions in different *fbx15* backgrounds; and AfGB67, AfGB68 and AfGB69 for *nic96::sgfp* in different *fbx15* backgrounds. Successful replacement of the original gene locus by the fusion construct was verified by Southern hybridization.

### Generation of RFP-tagged Fbx15 wild type and phosphomutant fusions

For the construction of *rfp*-tagged variants of *fbx15*, we started with the assembly of a plasmid containing *^t^trpC::ptrA::fbx15* 3’UTR. Therefore, we amplified the *^t^trpC* terminator from pAN7-1 with primers BJ309/BJ316, which introduced a *EcoR*V-site followed by an 15 bp pBluescript II KS+ overhang at the 5’end and a 15 bp *ptrA* overhang at the 3’end. *ptrA* was amplified with oligos OZG-(ptrA 5)/OZG-(ptrA 3) from pME3024. The *fbx15* 3’UTR flanking region was amplified from D141 genomic DNA with primer pair BJ317/BJ312, introducing a 15 bp overhang for *ptrA* at the 3’end and a 15 bp overhang for pBluescript II KS+ at the 5’end. All fragments were fused together into the *EcoR*V-site of pBluescript II KS+ by using the “GENEART Seamless Cloning and Assembly Kit” (invitrogen), resulting into plasmid pME4341.

For the fusion of *fbx15* with *rfp* we amplified *fbx15* together with a 1.5 kb 5’UTR flanking region comprising the native promoter of *fbx15* with oligos BJ313/BJ318, which adds a 15 bp overhang complementary to pBluescript II KS+ at the 5’end and in addition removes the stop-codon from the *fbx15* ORF. *rfp* was amplified from pChS4 (Dr. Christoph Sasse, personal communication) with primers BJ321/BJ322, introducing 15 bp overhangs complementary to *fbx15* and *^t^trpC*. Both fragments were fused into the *EcoR*V-site of pME4341, within a seamless cloning reaction. This resulted in plasmid pME4342 which comprises the *fbx15::rfp* fusion cassette *fbx15* 5’UTR*::fbx15::rfp::^t^trpC::ptrA::fbx15* 3’UTR, which is flanked by two *Mss*I-sites.

For the introduction of the mutated phospho-sites, which mimic a constant dephosphorylated state of Fbx15 at positions 468 and 469, we amplified a *fbx15* 5’UTR*::fbx15* part from pME4342 with oligos BJ313/BJ304. The second primer introduced a mutation in the *fbx15* ORF, which leads to the S468A exchange. As a second part we amplified the rest of the *fbx15* ORF together with the *rfp::^t^trpC::fbx15* 3’UTR with primers BJ306/BJ312. Here the first oligo adds a 15 bp overhang complementary to the first part of the *fbx15* ORF and in addition adds two mutations, which will lead to an exchange of S468A and S469A. Now both parts were fused again into *EcoR*V-site of pBluescript II KS+ with a seamless cloning reaction, resulting in pME4345. The exchange the serine residue at position 469 to aspartate to mimic a constant phosphorylation was achieved in the same way. First the 5’ part of *fbx15* 5’UTR*::fbx15* was amplified from pME4342 with BJ313/BJ336 leading to S469D. The second part was amplified with BJ337/BJ312 leading to *fbx15* [S469D]. The fragments were fused into pBluescript II KS+ with the “GENEART Seamless Cloning and Assembly Kit” (invitrogen), resulting into pME4348 [S469D].

The *fbx15::rfp* fusion cassettes were excised from their plasmids with *Mss*I and transformed into *A. fumigatus* strain AfGB64 which carries the *ssnF::gfp* fusion. The following strains were generated:

AfGB98 (*fbx15::rfp*)

AfGB101 (*fbx15*[S468|9A]*::rfp*)

AfGB102 (*fbx15*[S469D]*::rfp*)

Successful replacement of the original *fbx15*-locus with the *rfp*-fusion constructs was verified with Southern hybridization and in case of the phosphomutant versions of *fbx15::rfp* also with sequencing.

### Generation of RFP-tagged Fbx15 wild type and phosphomutant fusions that lack the F-box domain

The *fbx15* mutant AfGB128 was constructed by using the self-excising β-rec/*six* *ptrA* marker from psk485 including a controllable *^p^xylP* promoter

[8]. The *fbx15* 5’UTR flanking region was amplified from pME4342 with primer pair AA1/AA2, introducing a *Mss*I-site and 15 bp overhang for pBluescript II KS+ at the 5’end and 15 bp overhang for the self-excising β-rec/*six* *ptrA* marker at the 3’end. The 3’UTR flanking region was amplified from pME4342 with primer pair AA3/AA4, introducing a 15 bp overhang for the self-excising β-rec/*six* *ptrA* marker at the 5’end and a *Mss*I-site followed by a 15 bp overhang for pBluescript II KS+ at the 3’end. The self-excising β-rec/*six* *ptrA* marker was excised from psk485 with *Sfi*I and fused with the *fbx15* 5-UTR and *fbx15* 3’UTR flanking region into the *EcoRV*-site of pBluescript II KS+ within a seamless cloning reaction resulting in pME4538. The ∆*fbx15* marker cassette was excised from pME4538 with *Mss*I and transformed into strain AfS35. The *ptrA* marker was recycled from the created strain by growth on xylose-containing minimal medium resulting in *A. fumigatus* strain AfGB128.

For the construction of *rfp*-tagged variants of *fbx15* that lack the F-box domain, we amplified the 5’UTR flanking region of *fbx15* from pME4342 with primer pair BJ313/BJ371, introducing a 15 bp overhang for pBluescript II KS+ at the 5’end. A *fbx15*[∆F-box]*::rfp::^t^trpC* fragment was amplified from pME4342 with primer pair BJ372/BJ316, which introduced a start codon for *fbx15* instead of the F‑box domain followed by a 15 bp overhang for the *fbx15* 5’UTR flanking region. At the 3’end a 15 bp overhang for the *ptrA* marker was introduced. To include the phosphomutant versions of Fbx15 into our analysis the respective PCR-fragments *fbx15*[∆F-box, S468|9A]*::rfp::^t^trpC* and *fbx15*[∆F-box, S469D]*::rfp::^t^trpC* were amplified form plasmids pME4345 and pME4348 in the same way. A PCR-fragment containing the *ptrA* marker cassette followed by the *fbx15* 3’UTR flanking region, which harbors a 15 bp flanking region for pBluescript II KS+ at the 5’end, was amplified from pME4342 with primer pair BJ47/BJ312. The respective *fbx15* variants were fused with the 5’UTR and the *ptrA::*3’UTR fragments into the *EcoR*V-site of pBluescript II KS+, within a seamless cloning reaction. This resulted in plasmids pME4475, pME4476 and pME4477, which comprise the *fbx15*[∆F-box]*::rfp* fusion cassettes:

5’UTR*::fbx15*[∆F-box]*::rfp::^t^trpC::ptrA::*3’UTR, 5’UTR*::fbx15*[∆F‑box, S468|9A]*::rfp::^t^trpC::ptrA::*3’UTR and 5’UTR*::fbx15*[∆F‑box, S469D]*::rfp::^t^trpC::ptrA::*3’UTR respectively that are flanked by two *Mss*I-sites.

The *fbx15*[∆F-box]*::rfp* fusion cassettes were excised from their plasmids with *Mss*I and transformed into *A. fumigatus* strain AfGB128, which carries a *fbx15* deletion. The following strains were generated:

AfGB125 (*fbx15*[∆F-box]*::rfp*)

AfGB126 (*fbx15*[∆F-box, S468|9A]*::rfp*)

AfGB127 (*fbx15*[∆F-box, S469D]*::rfp*)

### Generation of strains for bimolecular fluorescence complementation

For the construction of BiFC-plasmids *A. fumigatus skpA* (Afu5g06060) was amplified from D141 genomic DNA with BJ69/BJ71 and fused to *cyfp* with OZG75/BJ71. The resulting module *cyfp::skpA* was cloned into *Pme*I-site of pME3160 [5] containing a bidirectional *niiA*/*niaD* nitrate inducible promoter system, resulting in pME4052. To test Fbx15/SkpA interaction *fbx15* was amplified from D141 genomic DNA with BJ66/BJ64 and subsequently fused to *nyfp* with OZG73/BJ64. The resulting module was cloned into *Swa*I-site of pME4052 resulting in pME4056. To test SconB/SkpA interaction *sconB* was amplified from D141 genomic DNA with BJ68/BJ24 and fused to *nyfp* with OZG73/BJ24. *nyfp::sconB* was then cloned into *Swa*I-site of pME4052 resulting in pME4058.

To examine the interaction Fbx15 and SsnF, *fbx15* was amplified from cDNA with BJ234/BJ235 and cloned into pBluescript II KS+ resulting in pME4298. The *fbx15* cDNA was amplified from pME4298 with BJ65/BJ66, fused to *cyfp* with OZG75/BJ64 and finally cloned into *Pme*I-site of pME3160 ending with pME4301. *ssnF* was amplified from cDNA with primer-pair BJ244/BJ245 and cloned into pBluescript II KS+ resulting in pME4300. *ssnF* cDNA was then amplified from the mentioned plasmid with BJ295/BJ245 and fused to *nyfp* with OZG73/BJ245. *nyfp::ssnF* was then cloned into *Swa*I-site of pME4301 resulting in pME4302.

The interaction between Fbx15 and GlcA or NimX was determined by generating PCR-fragments of *glcA* and *nimX* from cDNA with primer pairs BJ377/BJ378 and BJ379/BJ380 respectively, which introduce a 15 bp overhang for *nyfp* at the 5’end and a 15 bp overhang for the *^t^niaD* terminator sequence of pME4301. *nyfp* was amplified from pME4302 with primer pair BJ344/OZG387, which adds a 15 bp overhang for the *^p^niaD* promoter sequence of pME4301 at its 5’start. *glcA* and *nimX* were fused with *nyfp* into the SwaI-site of pME4301 in a seamless cloning reaction, resulting in pME4468 and pME4469.

pME4056, pME4058, pME4302, pME4468 and pME4469 were transformed into *A. fumigatus* strain Af293.1 containing a *pyrG1* mutation. Transformants AfGB44, AfGB45, AfGB93, AfGB123 and AfGB124 were incubated in London media (1 % (w/v) D‑glucose, 2 % salt solution (26 g/L KCl, 26 g/L MgSO_4_, 76 g/L KH_2_PO_4_, 50 ml/L trace element solution (40 mg/L Na_2_B_4_O_7_ * 10 H_2_O, 400 mg/L CuCl_2_ * 5 H_2_O, 800 mg/L FeCl_3_ * H_2_O, 800 mg/L MnSO_4_ * 4 H_2_O, 800 mg/L Na_2_MoO_4_ * 2 H_2_O, 8 g/L ZnSO_4_ * 7 H_2_O) pH6.5) containing ammonium as N-source to repress the nitrate promoter system or nitrate to induce the promoter.

### Heterokaryon rescue

The heterokaryon rescue for the deletion of *sconB*, *glcA*, *nimX* and *ssnF* was carried out as described [9]. First conidia of primary transformants were picked and equally plated on non-selective MM-medium and on selective MM-medium plates. The plates were incubated at 37°C for 3 days. From the transformants, which were still growing on the selective medium genomic DNA was extracted and a Southern hybridization was performed.

### Fluorescence microscopy

*A. fumigatus* strains were inoculated on sterile cover slips covered with 400 µl of liquid MM with supplements as shown. Cover slips were mounted on glass slides with nail polish. For BiFC microscopy respective strains were inoculated in 8-well borosilicate cover glass system (Thermo Scientific) containing London-medium supplemented with either (inducing) nitrate or (repressing) ammonium as stated above. For staining of nuclei grown hyphae were incubated with either 0.1 % 4’,6’-diamidino-2-phenylindole, DAPI (Roth) or 0.1 % Hoechst 33258 pentahydrate (Invitrogen), 20 min prior to microscopy. Fluorescence pictures were obtained from an Axiovert Observer Z1 (Zeiss) microscope equipped with a CoolSNAP ES2 (Photometrics) digital camera. All microscopy pictures were made with the SlideBook 5.0 or SlideBook 6.0 software package (Intelligent Imaging Innovations).

For BiFC signals at least 10 hyphae of two biological replicates were evaluated. Quantifications of YFP-signal intensities of interaction signals were done with the SlideBook 6.0 software. For quantifications the complete YFP-signal intensities of 10 hyphae with each 30 µM length incorporating 1-2 nuclei were compared to YFP-signal intensities, which were obtained from nuclear regions stained with Hoechst.

### Real-time-PCR

The expression of *fbx15* upon oxidative stress was measured with quantitative real-time PCR using a Light Cycler 2.0 System (Roche). The RNA from AfS35 (WT) cultures treated with 3 mM H_2_O_2_ for indicated time points was extracted with the “RNeasy plant mini kit” (Qiagen). 0.8 µg RNA of each sample was transcribed into cDNA using “QuantiTect reverse transcription kit” (Qiagen). The gene expression of *fbx15* was measured from 1:10 dilutions of the cDNA samples with primer pair BJ256/BJ257 and “RealMasterMix SYBR ROX 2.5x” (5Prime). Histone *h2A*- and Glyceraldehyde-3-phosphate dehydrogenase *gpdA*-expression was measured with primer pairs HO1/HO2 and Kt330/Kt331 respectively and used as reference. In total we made 3 independent runs for each time point with each reaction run in duplicates.

The expression of *cat1* was measured from cultures that were grown in minimal medium and treated with 3 mM H_2_O_2_ for indicated time points. For the expression of *gli-*genes, cultures were grown in Czapek-Dox medium at 28°C for 5 days. RNA-extraction and cDNA transcription were done as described above. The expression of *cat1* was determined from 1:10 diluted cDNA samples with primer pair BJ375/BJ376, whereas Real-Time PCR samples for *gli*-expression were prepared from 1:5 dilutions of the cDNA-samples using primer pairs GliZf/GliZr (*gliZ*), GliPf/GliPr (*gliP*), GliKf/GliKr (*gliK*) and GliTf/GliTr (*gliT*), which were previously described by Gardiner et al, 2005 [10] with “MESA GREEN qPCR MasterMix Plus for SYBR^®^ Assay” (Eurogentec). Gene-expressions were measured with a CFX Connect™ Real-Time System (Bio-Rad) and compared to *h2A* (HO1/HO2) and *gpdA* (Kt330/Kt331) expression. The real-time PCR data was analyzed with 2-∆∆C_T_ method for relative quantification of gene expression [11].

### Protein extraction

Crude protein extracts from vegetative mycelia were obtained by extraction from ground mycelia with B300 buffer (300 mM NaCl, 100 mM Tris-HCl pH 7.5, 10 % glycerol, 1 mM EDTA, 0.1 % NP‑40) supplemented with 1.5 ml/L 1 M DTT, Complete Protease Inhibitor Cocktail EDTA-free (Roche), 3 ml/L 0.5 M Benzamidine, 10 ml/L phosphatase inhibitors (100 mM NaF, 50 mM NaVanadate, 800 mM β‑glycerolephosphate) and 10 ml/L 100 mM PMSF.

### Antibodies used in this study

For the detection of GFP-signals α-GFP mouse antibody (SantaCruz) was used in 1:1000 dilution in TBS, containing 5 % (w/v) non-fat dry milk powder. TAP-signals were obtained using α-Calmodulin rabbit antibody (Millipore) in 1:2000 dilution in TBS 5 % dry milk. RFP-signals were detected with mouse monoclonal RFP [3F5] antibody (Chromotek) diluted 1:1000 in TBS 5 % dry milk. Ubiquitinated proteins were detected with a custom made polyclonal Anti-UbiA rabbit antibody (GenScript), which was used in 1:2000 dilution in TBST (0.05 % Tween‑20) including 5 % dry milk. Actin or tubulin used as loading control were detected with Anti-Actin rabbit antibody and α-tubulin mouse antibody (Sigma-Aldrich) both diluted 1:2000 in TBS 5 % dry milk. For the detection of the native Fbx15 protein from AfS35 (WT) cultures we used a custom made polyclonal Fbx15 rabbit antibody diluted 1:1000 in TBST (0.2 % Tween‑20) dry milk.

Phosphorylated Fbx15‑GFP protein was detected with Anti-Phosphoserine/threonine rabbit antibody (abcam) diluted 1:1000 in TBST (0.05 % Tween‑20) containing 3 % BSA. Membranes with purified Fbx15‑GFP were blocked in TBS containing 3 % BSA instead of 5 % non-fat dry milk powder.

As second antibody peroxidase coupled rabbit anti mouse (Jackson ImmunoResearch) or goat anti rabbit (Invitrogen) in 1:2500 dilution in TBS 5 % dry milk was used.

### Tandem affinity purification (TAP)

Tandem affinity purification of TAP-tagged proteins was performed with a modified version of the TAP-protocol as described previously [12]. In brief, TAP-tagged overexpression strains were grown in liquid MM for 24 hours at 37°C. Mycelia was harvested and washed with 0.96 % NaCl/ 1 % DMSO/ 1 % 100 mM PMSF. Crude extracts were prepared from ground mycelium with B300 buffer as described. Protein extracts were incubated with 300 µl of IgG sepharose 6 Fast Flow (GE Healthcare) for 3 hours at 4°C on a rotary shaker. The suspension was poured onto a Poly-prep chromatography column (BioRad) and washed once with 10 ml of IPP300 (25 mM Tris-HCl pH 8.0, 300 mM NaCl, 0.1 % NP‑40, 2 mM DTT), once with 10 ml IPP150 (25 mM Tris-HCl pH 8.0, 150 mM NaCl, 0.1 % NP‑40, 2 mM DTT), and once with TEV cleavage buffer (TEV-CB: 25 ml Tris-HCl pH 8.0, 150 mM NaCl, 0.1 % NP‑40, 0.5 mM EDTA, 1 mM DTT). The TEV cleavage was performed with 350 U of AcTEV (Invitrogen) in 1 ml of TEV-CB at 4°C for 12 hours. The eluate was poured into a new column containing 6 ml calmodulin binding buffer (CBB: 25 mM Tris-HCl pH 8.0, 150 mM NaCl, 1 mM Mg acetate, 1 mM imidazole, 2 mM CaCl_2_, 10 mM β‑mercaptoethanol), 6 µl 1 M CaCl_2_ and 300 µl of Calmodulin Affinity Resin (Agilent Technologies). The elution was repeated once with 1 ml of TEV‑CB and the eluate was incubated with calmodulin beads for 2 hours at 4°C on a rotary shaker. After incubation the beads were washed twice with CBB containing either 0.1 % NP‑40 or 0.02 % NP‑40. Proteins were eluted twice with 0.5 ml calmodulin elution buffer (CEB: 25 mM Tris-HCl pH 8.0, 150 mM NaCl, 0.02 % NP‑40, 1 mM Mg‑acetate, 1 mM imidazole, 20 mM EGTA, 10 mM β-mercaptoethanol) followed by precipitation with trichloroacetic acid (TCA) with a ratio of 1:4 for 30 minutes on ice and periodic vortexing. Proteins were pelleted by centrifugation and washed with acetone. The final pellet was resuspended in protein loading dye and separated by SDS-PAGE. The protein bands were stained by Coomassie Brilliant Blue G-Colloidal staining (Sigma), cut out from the SDS-polyacrylamide gel and further processed for LC-MS/MS identification.

### LC-MS/MS Protein identification

Proteins in the coomassie stained polyacrylamide pieces were in gel digested with trypsin [13] using “Sequencing Grade Modified Trypsin” (Promega). Digested peptides were extracted from polyacrylamide gel and separated using reversed-phase liquid chromatography with an *RSLCnano Ultimate 3000* system (Thermo Scientific) followed by mass identification with an *Orbitrap Velos Pro* mass spectrometer (Thermo Scientific). Chromatographically seperated peptides were on-line ionized by nano-electrospray (nESI) using the *Nanospray Flex Ion Source* (Thermo Scientific) at 2.4 kV and continuously transferred into the mass spectrometer. Full scans within m/z of 300-1850 were recorded by the Orbitrap-FT analyzer at a resolution of 30.000 (using m/z 445.120025 as lock mass) with parallel data-dependent top 10 MS2-fragmentation in the *LTQ Velos Pro* linear ion trap. LCMS method programming and data acquisition was performed with the software *XCalibur 2.2* (Thermo Scientific) and method/raw data validation with the program *RawMeat 2.1* (Vast Scientific). MS/MS2 data processing for protein analysis and identification was done with either MaxQuant quantitative proteomic software in conjunction with Perseus software for statistical analysis [14] or the *Proteome Discoverer 1.3* (*PD*, Thermo Scientific) and the *Discoverer Deamon Daemon 1.3* (Thermo Scientific) software using the Sequest (and/or Mascot) peptide analysis algorithm(s) and organism-specific taxon-defined protein databases extended by the most common contaminants. As controls either AfS35 (WT) for the TAP purifications or GFP- (AfGB70) and RFP-expressing (AfGB118, C. Sasse, personal communication) strains for GFP- and RFP-traps respectively were used.

### TMT isobaric mass tag labeling

The labeling reaction was done according to manufacturers protocol with slight modifications. Both labeling reagents TMT^2^-126 and TMT^2^-127 (each 0.8 mg) were solved in 41 µl of acetonitrile. Now 13 µl of the TMT^2^-127 labeling solution was added to the peptides from time point zero, while time points 20’ min, 40’ min and 60’ min were each mixed with 13 µl of TMT^2^-126 solution. The reactions were incubated at room temperature for 1 hour. Afterwards the labeling reaction was stopped by adding 8 µl of 5 % hydroxylamine for 20 min. The peptides from time points 20’, 40’ and 60’ min were equally mixed with the peptides from time point zero. The peptide mixtures were concentrated in a speed-vac and dissolved in 95 % H_2_O / 5 % acetonitrile / 0.1 % formic acid. The parameters for fragmentation during mass spectrometry were set to identify only the phosphorylated peptide of Fbx15 or two unmodified Fbx15 peptides. Specific ratios of the heavy labeled phosphopeptide were obtained from time point zero against the light labeled phosphopeptides from the other conditions. These values were quantified against the ratios of two unmodified reference peptides, which represented the overall amount of purified Fbx15.

### Gliotoxin measurement

To determine gliotoxin production levels the *A. fumigatus* strains were cultivated in Czapek-Dox medium at 28°C for 5 days. For quantification of gliotoxin the internal standard 4-nitrocatechol (1.12 µg/ml) similar to Jain et al. 2011 [15] was added to each culture and standard dilution series. Fungal cultures were extracted twice with equal volumes (v/v) ethyl acetate, dried with sodium sulfate and evaporated with a rotatory evaporator. They were then resuspended in 1 ml of methanol and 10 µl was injected onto a LC-MS system consisting of an HPLC, UltiMate 3000 binary RSLC with photo diode array detector (Thermo Fisher Scientific, Dreieich, Germany) and the mass spectrometer (LTQ XL Linear Ion Trap from Thermo Fisher Scientific) with an electrospray ion source. The liquid chromatography method consisted of the following gradient: initial MeCN/0.1 % (v/v) HCOOH (H_2_O) 0/100 and increasing to 80/20 in 15 min, then to 100/0 in 2 min, 2 min 100/0, and then back to 0/100 in 2 min with a flow rate 1 ml/min using an ACCUCORE RP-MS 2.6 µm 150x4.6 mm column (Thermo Fisher Scientific). A gliotoxin standard curve was created using the following concentrations: 200, 100, 50, 25, 5 and 1 ng/ul. The Xcalibur Quan Browser software (Thermo Fisher Scientific) was used to calculate the amounts of gliotoxin.

### Supplemental Text S1 References

1. Bailey TL, Elkan C. Fitting a mixture model by expectation maximization to discover motifs in biopolymers. Proc Int Conf Intell Syst Mol Biol. Department of Computer Science and Engineering, University of California, San Diego; 1994;: 28–36.

2. Kosugi S, Hasebe M, Tomita M, Yanagawa H. Systematic identification of cell cycle-dependent yeast nucleocytoplasmic shuttling proteins by prediction of composite motifs. Proc Natl Acad Sci USA. 2009;106: 10171–10176. doi:10.1073/pnas.0900604106

3. Blom N, Gammeltoft S, Brunak S. Sequence and Structure-Based Prediction of Eukaryotic Protein Phosphorylation Sites. J Mol Biol. 1999;294: 1351–1362. doi:10.1006/jmbi.1999.3310

4. Taus T, Köcher T, Pichler P, Paschke C, Schmidt A, Henrich C, et al. Universal and Confident Phosphorylation Site Localization Using phosphoRS. J Proteome Res. 2011;10: 5354–5362. doi:10.1021/pr200611n

5. Bayram Ö, Krappmann S, Ni M, Bok JW, Helmstaedt K, Valerius O, et al. VelB/VeA/LaeA Complex Coordinates Light Signal with Fungal Development and Secondary Metabolism. Science. 2008;320: 1504–1506. doi:10.1126/science.1155888

6. Szewczyk E, Nayak T, Oakley CE, Edgerton H, Xiong Y, Taheri-Talesh N, et al. Fusion PCR and gene targeting in *Aspergillus nidulans*. Nat Protoc. 2006;1: 3111–3120. doi:10.1038/nprot.2006.405

7. Punt PJ, van den Hondel CA. Transformation of filamentous fungi based on hygromycin B and phleomycin resistance markers. Meth Enzymol. 1992;216: 447–457.

8. Hartmann T, Dümig M, Jaber BM, Szewczyk E, Olbermann P, Morschhäuser J, et al. Validation of a Self-Excising Marker in the Human Pathogen *Aspergillus fumigatus* by Employing the β-Rec/*six* Site-Specific Recombination System. Appl Environ Microbiol. 2010;76: 6313–6317. doi:10.1128/AEM.00882-10

9. Osmani AH, Oakley BR, Osmani SA. Identification and analysis of essential *Aspergillus nidulans* genes using the heterokaryon rescue technique. Nat Protoc. 2006;1: 2517–2526. doi:10.1038/nprot.2006.406

10. Gardiner DM, Howlett BJ. Bioinformatic and expression analysis of the putative gliotoxin biosynthetic gene cluster of *Aspergillus fumigatus*. FEMS Microbiol Lett. 2005;248: 241–248. doi:10.1016/j.femsle.2005.05.046

11. Schmittgen TD, Livak KJ. Analyzing real-time PCR data by the comparative *C*T method. Nat Protoc. 2008;3: 1101–1108. doi:10.1038/nprot.2008.73

12. Bayram Ö, Bayram ÖS, Valerius O, Jöhnk B, Braus GH. Identification of Protein Complexes from Filamentous Fungi with Tandem Affinity Purification. 1st ed. Keller NP, Turner G, editors. Humana Press; 2012. doi:10.1007/978-1-62703-122-6

13. Shevchenko A, Wilm M, Vorm O, Mann M. Mass Spectrometric Sequencing of Proteins from Silver-Stained Polyacrylamide Gels. Anal Chem. 1996;68: 850–858. doi:10.1021/ac950914h

14. Cox J, Mann M. MaxQuant enables high peptide identification rates, individualized p.p.b.-range mass accuracies and proteome-wide protein quantification. Nat Biotechnol. 2008;26: 1367–1372. doi:10.1038/nbt.1511

15. Jain R, Valiante V, Remme N, Docimo T, Heinekamp T, Hertweck C, et al. The MAP kinase MpkA controls cell wall integrity, oxidative stress response, gliotoxin production and iron adaptation in *Aspergillus fumigatus*. Mol Microbiol. 2011;82: 39–53. doi:10.1111/j.1365-2958.2011.07778.x
